# Priority Indicators for Adolescent Health Measurement – Recommendations From the Global Action for Measurement of Adolescent Health (GAMA) Advisory Group

**DOI:** 10.1016/j.jadohealth.2022.04.015

**Published:** 2022-10

**Authors:** Andrew D. Marsh, Ann-Beth Moller, Elizabeth Saewyc, Emmanuel Adebayo, Elsie Akwara, Peter Azzopardi, Mariame Guèye Ba, Valentina Baltag, Krishna Bose, Stephanie Burrows, Liliana Carvajal, Saeed Dastgiri, Lucy Fagan, Jane Ferguson, Howard S. Friedman, Charity Giyava, Ann Hagell, Jo Inchley, Debra Jackson, Anna E. Kågesten, Aveneni Mangombe, Alison Morgan, Holly Newby, Linda Schultz, Marni Sommer, Ilene Speizer, Kun Tang, Regina Guthold

**Affiliations:** a Maternal, Newborn, Child and Adolescent Health and Ageing Department, WHO, Geneva, Switzerland; b Department of Sexual and Reproductive Health and Research, UNDP/UNFPA/UNICEF/WHO/World Bank Special Programme of Research, Development and Research Training in Human Reproduction (HRP), World Health Organization, Geneva, Switzerland; c Stigma and Resilience Among Vulnerable Youth Centre, School of Nursing, University of British Columbia, Vancouver, British Columbia, Canada; d Adolescent Health Unit, Institute of Child Health, College of Medicine, University of Ibadan, Ibadan, Nigeria; e Independent Consultant, Nairobi, Kenya; f Global Adolescent Health group, Burnet Institute, Melbourne, Victoria, Australia; g Adolescent Health and wellbeing program, South Australian Health and Medical Research Institute, Adelaide, South Australia, Australia; h University Cheikh Anta Diop of Dakar, Faculty of Medicine, Pharmacy and Odontology/Gynecology, Dakar, Senegal; i Obstetrics Clinic, University Teaching Hospital A. Le Dantec, Dakar, Senegal; j Bill and Melinda Gates Institute for Population and Reproductive Health, Bloomberg School of Public Health, Johns Hopkins University, Baltimore, Maryland; k Social Determinants of Health Department, WHO, Geneva, Switzerland; l Division of Data, Analytics, Planning and Monitoring, Data and Analytics Section, UNICEF, New York, New York; m Department of Global Public Health, Karolinska Institutet, Stockholm, Sweden; n Tabriz Health Services Management Research Center, Tabriz University of Medical Sciences, Tabriz, Iran; o UN Major Group for Children and Youth, London, United Kingdom; p Imperial College Healthcare NHS Trust, London, United Kingdom; q Independent Consultant, Adolescent Health, Tannay, Switzerland; r Technical Division, United Nations Population Fund, New York, New York; s Women Deliver, Young Leaders Alumni, Harare, Zimbabwe; t Association for Young People's Health, London, United Kingdom; u MRC/CSO Social and Public Health Sciences Unit, University of Glasgow, Glasgow, United Kingdom; v MARCH Centre, London School of Hygiene and Tropical Medicine, London, United Kingdom; w School of Public Health, University of the Western Cape, Cape Town, South Africa; x Zimbabwe LSHTM Research Partnership, Ministry of Health and Child Care, Harare, Zimbabwe; y Global Financing Facility, The World Bank Group, Washington, District of Columbia; z Nossal Institute for Global Health, Melbourne School of Population and Global Health, University of Melbourne, Melbourne, Victoria, Australia; aa Department of Sociomedical Sciences, Mailman School of Public Health, Columbia University, New York, New York; ab Department of Maternal and Child Health, Gillings School of Global Public Health, University of North Carolina at Chapel Hill, Chapel Hill, North Carolina; ac Vanke School of Public Health, Tsinghua University, Beijing, China

**Keywords:** Adolescent, Adolescent health, Youth health, Adolescent health services, Global health, Health status indicators, Social determinants of health, Health behavior, Health risk behavior

## Abstract

**Purpose:**

This article describes the selection of priority indicators for adolescent (10–19 years) health measurement proposed by the Global Action for Measurement of Adolescent health advisory group and partners, building on previous work identifying 33 core measurement areas and mapping 413 indicators across these areas.

**Methods:**

The indicator selection process considered inputs from a broad range of stakeholders through a structured four-step approach: (1) definition of selection criteria and indicator scoring; (2) development of a draft list of indicators with metadata; (3) collection of public feedback through a survey; and (4) review of the feedback and finalization of the indicator list. As a part of the process, measurement gaps were also identified.

**Results:**

Fifty-two priority indicators were identified, including 36 core indicators considered to be most important for measuring the health of all adolescents, one alternative indicator for settings where measuring the core indicator is not feasible, and 15 additional indicators for settings where further detail on a topic would add value. Of these indicators, 17 (33%) measure health behaviors and risks, 16 (31%) health outcomes and conditions, eight (15%) health determinants, five (10%) systems performance and interventions, four (8%) policies, programmes, laws, and two (4%) subjective well-being.

**Discussion:**

A consensus list of priority indicators with metadata covering the most important health issues for adolescents was developed with structured inputs from a broad range of stakeholders. This list will now be pilot tested to assess the feasibility of indicator data collection to inform global, regional, national, and sub-national monitoring.

**Implications and Contribution**

The priority indicators for adolescent health measurement and proposed metadata contribute to focusing data collection on the most important health issues for adolescents and to harmonizing measurement and reporting. This can enable targeted action and ultimately improve adolescent health at the country and global levels.

Adolescence, defined as the period from ages 10 to 19, [1] is a critical stage in life with significant physical, emotional, cognitive, and social development. [2] The protection and promotion of health during this life stage is of great importance, and has been shown to yield benefits not only for adolescents now, but also for their future adult lives and for their future children. [3,4]

Consistent and harmonized data are needed to guide actions toward improving adolescent health and to track progress toward and ultimately achieve global development goals. [5,6] However, measurement of adolescent health across and within countries has been inconsistent in the past. [7] While many global accountability initiatives have included indicators of adolescent health, the selected indicators, their definitions and proposed measurement details often vary. [8] Furthermore, some topics have benefited from greater attention with many indicators being proposed for their monitoring, while other topics have been neglected. [9] This situation has made it difficult to obtain comparable data across countries and over time, resulting in important knowledge and accountability gaps for improving adolescent health at country and global levels.

With the overarching goal to achieve consistent measurement of adolescent health at country and global levels, World Health Organization (WHO), in collaboration with the Joint United Nations (UN) Program on human immunodeficiency virus and Acquired Immune Deficiency Syndrome, the UN Educational, Scientific, and Cultural Organization, the UN Population Fund, the UN Children’s Fund, UN Women, the World Bank Group, and the World Food Programme, established the Global Action for Measurement of Adolescent health (GAMA) advisory group (AG) in 2018. [10] The GAMA AG consists of 16 adolescent health measurement experts, including four young experts, selected through a competitive process following an open call. [10] The GAMA AG serves to coordinate, harmonize, and share evidence on adolescent health measurement among the AG and other stakeholders, and to work towards specific objectives. These objectives include the selection of priority adolescent health indicators and development of harmonized guidance for their measurement, as well as their subsequent promotion of and support for country implementation. [11]

The approach to selecting these indicators includes several stages, the first two of which were completed in 2020. [8,12] First, 33 core areas for adolescent health measurement were systematically identified based on input from youth organizations and country representatives and reviews of country priorities, disease burden, and existing measurement efforts. The core areas were organized under six measurement domains: health determinants; health behaviors and risks; policies, programs, and laws; systems performance and interventions; subjective well-being; and health outcomes and conditions. [12] Second, adolescent health indicators included in 16 previously identified regional and global measurement initiatives and measuring any aspect of the 33 core measurement areas were mapped, resulting in the identification of 413 indicators. [8] Indicators assessing multiple measurement areas were listed in the area of the indicator's primary focus. Substantial overlap was noted among the mapped indicators with many initiatives proposing indicators measuring the same construct but differing in their proposed metadata, resulting in a set of 236 distinct indicators. [8] A list of the 16 measurement initiatives and 33 core measurement areas is available in Appendix A.

This article describes the results from the third stage, the structured selection of priority indicators for adolescent health measurement and their metadata from the 413 previously mapped indicators along with the documentation of measurement gaps. In the fourth stage, initiated in 2022, these priority indicators are being tested in selected countries to evaluate their feasibility for measuring adolescent health at the country and global levels and inform further revisions.

## Methods

### Overview of the indicator selection process

**Figure 1:**
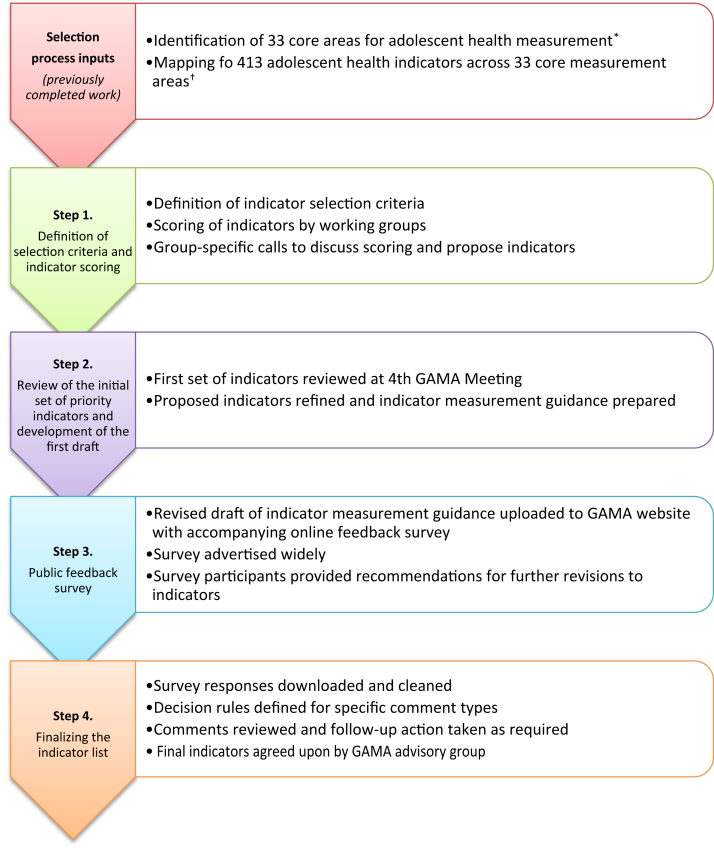
Flow diagram of indicator selection process. ∗ See Guthold et al. (2020) [12]. ^†^ See Newby et al. (2020) [8].

The indicator selection process applied a structured approach to reach consensus on a set of priority indicators for measuring adolescent health at the country and global levels, with inputs collected from a broad range of stakeholders and primary decision-making responsibility resting with the GAMA AG. The process built upon the previous identification of core adolescent health measurement areas [12] and indicator mapping. [8] The process began at the third GAMA Meeting (February 2020, Cape Town), facilitated by WHO and attended by experts of the GAMA AG, representatives of UN agencies and of four African countries (Democratic Republic of the Congo, Ivory Coast, Nigeria and Zimbabwe) participating in the Flagship Programme for Adolescent Health of WHO's African Region, with expressed interest in improving their measurement systems (collectively referred to as the ‘core selection team’ hereafter). The process included four steps: (1) definition of selection criteria and scoring of each of the 413 indicators, resulting in an initial set of indicators; (2) review of this initial set and development of a first draft list of indicators; (3) collection of public feedback on the draft list through an online survey published on the WHO website; and (4) review of the feedback received and finalization of the list of indicators (Figure 1). Measurement gaps were identified and reviewed at each stage. All feedback was considered equally. Information about each step is provided below.

### Step 1: Definition of selection criteria and indicator scoring

At the third GAMA Meeting, the core selection team reviewed and discussed existing indicator guidance to define indicator selection criteria. [7,[13], [14], [15]] Criteria deemed relevant for the indicator scoring was tested with selected indicators in group work sessions during the meeting, resulting in four final criteria: relevance, feasibility, validity, and usefulness (Box 1).

Box 1 **Indicator selection criteria∗**

• Relevance: The indicator measures a specific construct in a priority area of interest and there is a clear, demonstrated relationship between the indicator and adolescent health.
• Feasibility: Data for the indicator can be obtained with reasonable and affordable effort.
• Validity: The indicator provides a robust assessment of the construct of interest, is sensitive to change in that construct, and has been field tested. The method of assessment produces consistent results that are comparable across time periods and settings.
• Usefulness: The indicator captures information that is easily understood and timely. The information is easy to communicate to stakeholders and facilitates investment and action in adolescent health strategies, priorities, or programming.

∗Developed based a review of existing indicator selection guidance [7,[13], [14], [15]].

Following the meeting, the previously mapped indicators were organized by theme (e.g., sexual and reproductive health, violence, and injury) and a working group was formed from among the core selection team for each theme. Group members were provided with the subset of indicators for their theme and asked to score each indicator independently for each criterion on a five-point scale. This exercise considered the complete set of 413 indicators rather than the subset of distinct indicators to allow for consideration of all variants of overlapping indicators, most of which differed in one or more aspect of their formulation. [8] The summary score for each indicator was calculated as the average score across all group members and all criteria, weighting each criterion equally. Indicators were sorted by summary score to show their relative performance and scoring results were circulated to group members.

One video call was held per working group to review indicator scoring results and propose core, additional, and alternate indicators (Box 2). Working groups attempted to propose at least one indicator for each core measurement area. Proposed indicators, which reflected scoring results and the subsequent working group discussions, were compiled across all working groups to form the initial set of priority indicators for adolescent health measurement.

Box 2 **Indicator types**

• Core indicators were considered to be the most important for measuring the health of all adolescents globally.
• An alternative indicator was proposed in one case where the priority indicator may not be feasible or may be too sensitive to measure in all contexts.
• Additional indicators were provided for settings where further detail within a specific area would add value, and resources for data collection and reporting are available.

### Step 2: Review of the initial set of indicators and development of the first draft indicator list

This initial set of indicators were reviewed during the fourth GAMA Meeting (June 2020, virtual). Indicators within each previously defined core measurement area were reviewed through the lens of the four selection criteria, followed by a discussion of the complete set of indicators. Feedback was solicited on the number of indicators proposed, their balance among measurement domains, and the overall coherence of the set of indicators.

The proposed set of indicators was subsequently refined and indicator measurement guidance with information for each indicator was prepared. Indicator names and metadata were populated using information from the indicator mapping, which was in turn based on measurement recommendations from initiatives proposing each indicator. [8] Indicator metadata included name, definition, numerator, denominator, data source, age range, disaggregation, type, user status and were standardized and adjusted based on inputs received during the meeting. The indicator measurement guidance was circulated among the meeting participants for written comments and further revised.

### Step 3: Public feedback survey

The resulting draft was uploaded to the GAMA website [11] and an accompanying online survey was developed in LimeSurvey [16] to collect indicator-specific feedback, feedback on the framing of the indicator measurement guidance, and an overall feedback on the full set of indicators, in addition to participant background details (Appendix B). To encourage broad stakeholder participation, survey was open to the public and was announced through a combination of targeted outreach and public announcements. Distribution was defined based on expert input and a review of the relevant literature and included the Permanent Missions to the United Nations, measurement experts, youth organizations, and other relevant groups. The survey was disseminated to these stakeholders through targeted e-mails to individuals and relevant listservs and recipients were encouraged to further circulate the announcement among their networks. In parallel, the feedback survey was promoted through social media posts and online announcements by relevant organizations, including a post on the main WHO webpage. [17] Both the indicator measurement guidance and feedback survey were available in English, French, and Spanish.

Survey participants selected the indicators for which they wanted to provide feedback. For each indicator, participants were presented with the proposed metadata and asked to indicate their recommendation for the indicator: keep as is, keep with changes, replace with another indicator, or drop. For responses other than “keep as is”, follow-up questions solicited additional details in text fields and the rationale for the suggestions.

### Step 4: Finalizing the indicator list

Survey responses were reviewed by the WHO GAMA Secretariat and high-level recommendations (e.g., “keep with changes”) were further classified according to the specific recommendation (e.g., “modify the population of interest”).

The WHO GAMA Secretariat and AG defined decision rules for specific comment types. Given GAMA's adolescent-specific focus, requests to amend the proposed age range beyond 10–19 years were declined. Requests to include disaggregation beyond sex and five-year age groups at the indicator-specific level were also declined to focus reporting efforts on currently recommended age disaggregation [18] and minimize the additional reporting burden. However, a list of potential characteristics to consider for disaggregation, such as belonging to a vulnerable population, was prepared based on participant responses and appears in the background to the indicator measurement guidance to enable further monitoring of health equity where resources are available. Lastly, recommendations to drop an indicator were considered only when survey participants from at least two WHO regions recommended this action. [19]

All other comments were reviewed individually and classified according to the required follow-up action. Comments requiring further input were shared with the relevant global experts. For example, comments on indicators assessing adolescent heavy episodic drinking were reviewed by members of the GAMA AG with expertise in this field and with members of WHO's Alcohol, Drugs and Addictive Behaviors unit. A final set of comments requiring further discussion was presented for review during the fifth and sixth GAMA Meetings (December 2020 and April 2021, both virtual).

After these meetings and as the last step in the process, GAMA AG members conducted a final review of the proposed indicators. To allow for a more in-depth review, they organized themselves into working groups for each of the six adolescent health measurement domains (Appendix A). Working group members collaboratively reviewed the indicators within their selected domain; one video call was held for each of the groups to address issues where further discussion was required. Based on these final inputs, the proposed list of indicators and accompanying measurement guidance were revised to their final draft.

### Measurement gaps

A parallel process was undertaken to document gaps where no mapped indicator was identified, such as adolescent disability or emergent health issues. At each step, participants were asked to identify measurement gaps and, where available, propose indicators for their measurement. The WHO GAMA Secretariat screened the identified measurement gaps for their relevance to adolescents globally and their alignment with the 33 previously identified core measurement areas with two goals in mind. First, where measurable indicators were proposed to assess measurement gaps, the core selection team reviewed these indicators for inclusion in the final list with consideration given to the four selection criteria and the frequency of the indicator's recommendation. Second, measurement gaps for which no indicator was identified were documented to inform future indicator development.

## Results

Between April 2020 and October 2021, 261 participants from 67 Member States across all WHO regions were involved in the indicator selection process (Appendix C). This included the core selection team (n = 52), additional experts consulted during the process (n = 65), such as adolescent health Regional Advisors of WHO Regional Offices, [20] WHO Headquarters Technical Officers, and staff from other UN agencies, and individuals participating only in the online feedback survey (n = 144). Six members of the core selection team and six additional experts also completed the online survey, resulting in 156 total survey respondents. Participants in the selection process were most commonly employed by international organizations (42%), followed by governments (20%), non-governmental and community-based organizations (17%), and academia (16%) (Table 1). Participant age data were only collected through the online feedback survey. Of the 123 survey respondents providing age data, 12 (10%) were less than 30 years old, 96 (78%) were 30–59 years old, and 15 (12%) were 60 or more years old (Appendix D).

**Table 1:** Summary of participant involvement in indicator selection process

	N (%)
Total participants	261 (100%)
Sector	
International organization	110 (42%)
NGO/CBO	45 (17%)
Government	52 (20%)
Academia	43 (16%)
Other/not specified	11 (4%)

Based on all participant inputs, the GAMA AG selected 52 priority indicators for the measurement of adolescent health, including 36 core, one alternative, and 15 additional indicators (Box 2) across all six adolescent health domains (Table 2, Appendix E). Selected indicators correspond to 32 of the 33 core measurement areas, [12] with no suitable indicator identified to assess disability. [8] Of the 52 selected indicators, 17 (33%) pertain to health behaviors and risks, 16 (31%) to health outcomes and conditions, eight (15%) to health determinants, five (10%) to systems performance and interventions, four (8%) to policies, programs, laws, and two (4%) to subjective well-being.

**Table 2:** Priority indicators recommended by the GAMA AG for adolescent health measurement by domain and indicator type

Indicator name	Initiatives including this indicator	Use status
Domain 1: Social, cultural, economic, educational, environmental determinants of health		
Core indicators^b^		
1.01 Proportion of total population that are adolescents (10–19 years), by age group (10–14, 15–19 years), and sex	EMRO core indicators for adolescent health^a^	In use
1.02 Proportion of young people who have completed primary, lower secondary, and upper secondary school, by level, and sex	Adolescent country tracker; Lancet Commission on Adolescent Health and Wellbeing; SDGs^a^	In use
1.03 Proportion of adolescents (10–19 years) who live below the national poverty line, by age group (10–14, 15–19 years), and sex	INSPIRE; SDGs^a^; UNECE Monitoring Framework (ICPD)	In use, modified by GAMA AG
Alternative indicator for 1.03: 1.03-ALT Proportion of adolescents (10–19 years) who live below the international poverty line, by age group (10–-14, 15–19 years), and sex	Adolescent country tracker; SDGs^a^	In use, modified by GAMA AG
1.04 Proportion of adolescents (10–19 years) who live with moderate or severe food insecurity, based on the Food Insecurity Experience Scale (FIES), by age group (10–14, 15–19 years), and sex	INSPIRE; SDGs^a^	In use, modified by GAMA AG
1.05 Proportion of female adolescents (15–19 years) who make their own informed decisions regarding sexual relations, contraceptive use, and reproductive health care	Global Strategy for WCAH; SDGs^a^; UNECE Monitoring Framework (ICPD)	In use
1.06 Proportion of adolescents (10–19 years) not in education, employment, or training, by age group (10–14, 15–19 years), and sex	Global Strategy for WCAH; Lancet Commission on Adolescent Health and Wellbeing; SDGs^a^; UNECE Monitoring Framework (ICPD); Youth Development Index	In use, modified by GAMA AG
Additional indicators		
A1.01 Proportion of adolescents (10–19 years) at the end of primary and at the end of lower secondary achieving at least a minimum proficiency level in (1) reading and (2) mathematics, by age group (10–14, 15–19 years), and sex	Global Strategy for WCAH; SDGs^a^	In use
Domain 2: Health behaviors and risks		
Core indicators		
2.01 Prevalence of overweight and obesity among adolescents (10–19 years), by weight status (overweight, obese), age group (10–14, 15–19 years), and sex	FRESH; Global Reference List of 100 Core Health Indicators; Global Reference List of Health Indicators for Adolescents^a^; Lancet Commission on Adolescent Health and Wellbeing; UNECE Monitoring Framework (ICPD)	In use
2.02 Prevalence of thinness among adolescents (10–19 years), by age group (10–14, 15–19 years), and sex	EMRO core indicators for adolescent health; Global Reference List of Health Indicators for Adolescents^a^	In use
2.03 Past 30 day prevalence of heavy episodic drinking among adolescents (10–19 years), by age group (10–-14, 15–19 years), and sex	INSPIRE; Lancet Commission on Adolescent Health and Wellbeing; Global Information System on Alcohol and Health^a^	In use, modified by GAMA AG
2.04 Past 30 day prevalence of psychoactive drug use among adolescents (10–19 years), by type of substances, age group (10–-14, 15–19 years), and sex	Adolescent country tracker; EMRO core indicators for adolescent health^a^; Youth Development Index	In use, modified by GAMA AG
2.05 Past 30 day prevalence of tobacco use among adolescents (10–19 years), by type of tobacco used, age group (10–14, 15–19 years), and sex	EMRO core indicators for adolescent health^a^; FRESH; Global Reference List of Health Indicators for Adolescents	In use
2.06 Proportion of adolescents (10–19 years) who consumed at least 5 servings of vegetables and fruits daily during the past 7 days, by age group (10–14, 15–19 years), and sex	EMRO core indicators for adolescent health^a^	In use
2.07 Proportion of adolescents (10–19 years) who accumulated an average of at least 60 minutes per day of moderate to vigorous-intensity physical activity during the past 7 days, by age group (10–14, 15–19 years), and sex	EMRO core indicators for adolescent health; FRESH; Global Reference List of Health Indicators for Adolescents^a^; Global Strategy for WCAH; WHO GPW 13	In use, modified by GAMA AG
2.08 Proportion of adolescents (10–19 years) involved in bullying within the past 12 months, by type of involvement (victim, perpetrator, both), type of bullying (in-person, digital/cyber), age group (10–14, 15–19 years), and sex	Adolescent country tracker; FRESH; INSPIRE; SDGs^a^; UNECE Monitoring Framework (ICPD)	In use, modified by GAMA AG
2.09 Proportion of adolescents (15–19 years) who had their first sexual intercourse before 15 years of age, by sex	FRESH; Global Reference List of Health Indicators for Adolescents; INSPIRE^a^; Measuring the Education Sector Response to HIV and AIDS	In use
2.10 Proportion of adolescents (10–19 years) who used a condom at last sexual intercourse, by age group (10–14, 15–19 years), and sex	FRESH; UNECE Monitoring Framework (ICPD)^a^	In use, modified by GAMA AG
2.11 Proportion of live births to female adolescents (10–19 years) attended by skilled health personnel, by age group (10–14, 15–19 years)	Countdown to 2030^a^; EMRO core indicators for adolescent health; Global Reference List of 100 Core Health Indicators; Global Strategy for WCAH; SDGs; UNECE Monitoring Framework (ICPD); WHO GPW 13	In use, modified by GAMA AG
2.12 Proportion of adolescents (10–19 years) who used a contraceptive (modern method) at last sexual intercourse, by method used, age group (10–14, 15–19 years), and sex	Countdown to 2030^a^; FP2020; Global Reference List of 100 Core Health Indicators; UNECE Monitoring Framework (ICPD)	In use, modified by GAMA AG
2.13 Proportion of adolescents (10–19 years) who have their need for contraception satisfied with modern methods, by age group (10–14, 15–19 years), and sex	Countdown to 2030; FP2020; Global Reference List of 100 Core Health Indicators; Global Reference List of Health Indicators for Adolescents; Global Strategy for WCAH; Lancet Commission on Adolescent Health and Wellbeing; SDGs^a^; UNECE Monitoring Framework (ICPD); WHO GPW 13	In use, modified by GAMA AG
Additional indicators		
A2.01 Past 30 day prevalence of alcohol use among adolescents (10–19 years), by age group (10–14, 15–19 years), and sex	FRESH; Global Reference List of Health Indicators for Adolescents^a^	In use
A2.02 Proportion of adolescents (10–19 years) who drank sugar-sweetened beverages one or more times per day during the past 7 days, by age group (10–14, 15–19 years), and sex	FRESH^a^	In use, modified by GAMA AG
A2.03 Proportion of female adolescents (10–19 years) who were aware of menstruation before menarche, by age group (10–14, 15–19 years)	None	Not currently in use
A2.04 Past 30 day prevalence of electronic cigarette use among adolescents (10–19 years), by age group (10–14, 15–19 years), and sex	Global Youth Tobacco Survey^a^	In use, modified by GAMA AG
Domain 3: Policies, programmes, and laws		
Core indicators		
3.01 Existence of an operational adolescent (10–19 years) health programme with coverage at the national level	Countdown to 2030^a^	In use, modified by GAMA AG
3.02 Existence of national standards for delivery of health services to adolescents (10–19 years)	Countdown to 2030^a^	In use
Additional indicators		
A3.01 Existence of national policy exempting adolescents (10–19 years) from user fees for specified health services in the public sector, by type of service	WHO SRMNCAH Policy Survey^a^	In use
A3.02 Absence of legal age limit for married and unmarried adolescents (10–19 years) to provide consent, without spousal/parental/legal guardian consent, for specified adolescent health services, by marital status, and type of service	WHO SRMNCAH Policy Survey^a^	In use
Domain 4: Systems performance and interventions		
Core indicators		
4.01 Proportion of adolescents (10–19 years) who made a visit to a health facility to receive a health service during the past 12 months, by age group (10–14, 15–19 years) and sex	Global Reference List of Health Indicators for Adolescents^a^	Not currently in use
4.02 Proportion of 15-year-old adolescents covered by human papilloma virus (HPV) vaccine (last dose in schedule), by sex	Global Strategy for WCAH; SDGs^a^	In use
4.03 Existence of age- and sex-disaggregated health data for adolescents (10–19 years) in the national health information system	EMRO core indicators for adolescent health^a^	In use
Additional indicators		
A4.01 Proportion of schools that offer comprehensive school health services	None	Not currently in use
A4.02 Proportion of schools that offer life skills-based HIV and sexuality education during the previous academic year	SDGs^a^; UNECE Monitoring Framework (ICPD)	In use
Domain 5: Subjective well-being		
Core indicators		
5.01 Proportion of adolescents (10–19 years) with someone to talk to when they have a worry or problem, by age group (10–14, 15–19 years) and sex	MMAP^a^	In use
Additional indicators		
A5.01 Proportion of adolescents (10–19 years) with a positive connection with their parent or guardian, by age group (10–14, 15–19 years) and sex	MMAP^a^	In use
Domain 6: Health outcomes and conditions		
Core indicators		
6.01 Adolescent (10–19 years) mortality rate, by age group (10–14, 15–19 years) and sex	Adolescent country tracker; Countdown to 2030; Global Reference List of 100 Core Health Indicators; Global Reference List of Health Indicators for Adolescents; Global Strategy for WCAH^a^; Youth Development Index	In use
6.02 Adolescent (10–19 years) mortality rate, by specified causes of death, age group (10–14, 15–19 years) and sex	Countdown to 2030^a^; Global Strategy for WCAH	In use, modified by GAMA AG
6.03 Number of new cases of HIV infections among adolescents (10–19 years) per 1,000 uninfected adolescent population, by age group (10–14, 15–19 years) and sex	Global Reference List of 100 Core Health Indicators; Global Strategy for WCAH; SDGs^a^; UNECE Monitoring Framework (ICPD); WHO GPW 13	In use
6.04 Number of new cases of sexually transmitted infections (STIs) among adolescents (10–19 years), by age group (10–14, 15–19 years) and sex	Global Reference List of 100 Core Health Indicators^a^	In use, modified by GAMA AG
6.05 Proportion of adolescents (10–19 years) who report a suicide attempt during the past 12 months, by age group (10–14, 15–19 years) and sex	EMRO core indicators for adolescent health; MMAP^a^	In use
6.06 Proportion of adolescents (10–19 years) who report symptoms of depression and/or anxiety, by age group (10–14, 15–19 years) and sex	EMRO core indicators for adolescent health; Global Reference List of Health Indicators for Adolescents; Global Strategy for WCAH; MMAP^a^	Not currently in use
6.07 Number of new cases of specified types of injuries among adolescents (10–19 years) per 100,000 population, type of injury, age group (10–14, 15–19 years) and sex	EMRO core indicators for adolescent health^a^	In use
6.08 Proportion of adolescents (10–19 years) who experienced physical violence during the past 12 months, by perpetrator (parents/caregivers, teachers, other adults, intimate partners, peers), age group (10–14, 15–19 years) and sex	FRESH; INSPIRE^a^	In use, modified by GAMA AG
6.09 Proportion of adolescents (10–19 years) who experienced contact sexual violence during the past 12 months, by perpetrator (parents/caregivers, teachers, other adults, intimate partners, peers), age group (10–14, 15–19 years) and sex	INSPIRE^a^	In use, modified by GAMA AG
6.10 Adolescent (10–19 years) birth rate, by age group (10–14, 15–19 years)	Adolescent country tracker; Countdown to 2030; EMRO core indicators for adolescent health; FP2020; Global Reference List of 100 Core Health Indicators; Global Reference List of Health Indicators for Adolescents^a^; Global Strategy for WCAH; Lancet Commission on Adolescent Health and Wellbeing; SDGs; UNECE Monitoring Framework (ICPD); Youth Development Index	In use, modified by GAMA AG
6.11 Prevalence of anemia among adolescents (10–19 years), by age group (10–14, 15–19 years) and sex	EMRO core indicators for adolescent health^a^; Global Strategy for WCAH; Lancet Commission on Adolescent Health and Wellbeing	In use
Additional indicators		
A6.01 Proportion of adolescents (10–19 years) perpetrating physical violence during the past 12 months, by age group (10–14, 15–19 years) and sex	None	Not currently in use
A6.02 Proportion of young women and men (18–29 years) who experienced sexual violence by age 18, by perpetrator (parents/caregivers, teachers, intimate partners, peers), age at victimization (<10, 10–14, 15–18 years) and sex	Global Reference List of 100 Core Health Indicators; Global Strategy for WCAH; INSPIRE; SDGs^a^; UNECE Monitoring Framework (ICPD)	In use
A6.03 Proportion of adolescents (10–19 years) who report suicidal thoughts during the past 2 weeks, by age group (10–14, 15–19 years) and sex	EMRO core indicators for adolescent health; MMAP^a^	In use, modified by GAMA AG
A6.04 Proportion of adolescents (10–19 years) with symptoms of anxiety or depression who report contact with a health professional or counsellor for their mental health symptoms, by age group (10–14, 15–19 years) and sex	Global Reference List of 100 Core Health Indicators; MMAP^a^	Not currently in use
A6.05 Proportion of female adolescents (10–19 years) who have undergone female genital mutilation/cutting, by age group (10–14, 15–19 years)	Countdown to 2030; Global Reference List of 100 Core Health Indicators; SDGs^a^	In use, modified by GAMA AG

a Metadata for the proposed indicator were derived primarily from this initiative.

b An alternative indicator (1.03-ALT) is listed below the linked core indicator (1.03).

Forty-six (88%) indicators are currently in use by one or more regional or global measurement initiatives with adolescent-specific indicators, of which 24 (52%) indicators are in use with metadata that were an exact match and 22 (48%) are in use with metadata that were a partial match to the indicators proposed here. For example, Indicator 1.02 “Proportion of young people who have completed primary, lower secondary, and upper secondary school, by level and sex” (Table 2) is currently included within the Sustainable Development Goals (indicator 4.1.2). [21] In contrast, Indicator 1.03 “Proportion of adolescents (10–19 years) who live below the national poverty line, by age group (10–14, 15–19 years) and sex” modifies Sustainable Development Goals 1.2.1 [21] to focus specifically on the proportion of adolescents rather than the proportion of the total population falling below the national poverty line.

Of the six indicators not currently in use, two were adopted from the Measurement of Mental Health among Adolescents at the Population Level initiative. [22] While these Measurement of Mental Health among Adolescents at the Population Level indicators are not currently in use, the data systems for their collection are under development and their use is expected from 2022 onwards. Of the remaining four indicators not currently in use, Indicator 4.01 “Proportion of adolescents (10–19 years) who made a visit to a health facility to receive a health service during the past 12 months, by age group (10–14, 15–19 years) and sex” was adapted from an initiative which had noted that the indicator requires further development. [23] Three new indicators were proposed that did not exist in any of the reviewed measurement initiatives, such as the A4.01 “Proportion of schools that offer comprehensive school health services”, the metadata for which was developed to align with the recently published WHO guidelines on school health services. [24]

**Figure 2:**
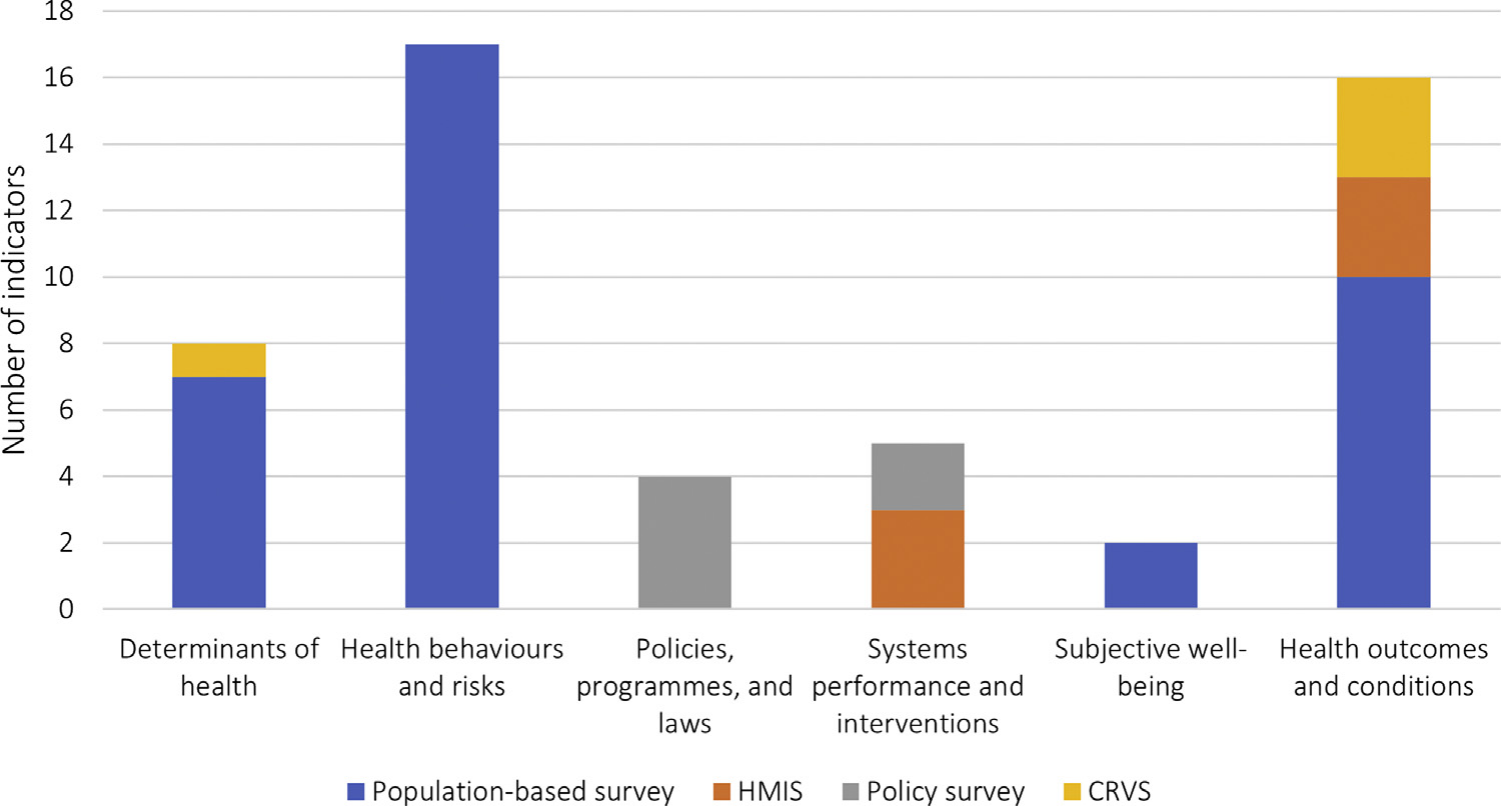
Priority indicators recommended by the GAMA AG by preferred data source and domain. Note: HMIS = health management information systems; CRVS = civil registration and vital statistics.

The preferred and alternative data sources noted for each indicator was identified by the GAMA AG based on the original metadata and participant inputs (Figure 2). Population-based surveys were the preferred data source for 36 indicators (69%), followed by health management information systems and policy surveys with six indicators each (23% combined), and civil registration and vital statistics with four indicators (8%).

During the selection process, measurement gaps were identified and reviewed for each of the six adolescent health domains. Identified measurement gaps in adolescent health were categorized into two types: First, health topics for which indicators are either under development or in use in populations other than adolescents. These indicators were considered for inclusion in the priority indicator list, as was the case, for example, with the indicators measuring menstrual health and hygiene and electronic cigarette use. Second, health topics for which no agreed indicators currently exist, but were deemed relevant for adolescents by participants. This included, for instance, topics in sexual and reproductive health of younger adolescents (e.g., body pride, puberty, and pre-coital sexual activity), online behaviors, and adolescents not in stable housing. A summary of these measurement gaps was compiled and is presented in Box 3.

Box 3 **Measurement gaps by adolescent health domain**

• Domain 1: Social, cultural, economic, educational, environmental determinants of health
  ○ Adolescents not in stable housing
  ○ Climate change and health
  ○ Gender equality
  ○ Gender identity
  ○ Sexual orientation
• Domain 2: Health behaviors and risks
  ○ Access to healthy food
  ○ Active travel
  ○ Addictive behaviors, particularly gaming disorder
  ○ Causes for abnormal weight status
  ○ Comprehensive abortion care
  ○ Counselling bias for contraception methods (e.g., adolescents not counselled on all methods, such as emergency contraception)
  ○ Eating disorders
  ○ Online behaviors, screen time
  ○ Pre-coital sexual activity
  ○ Preparedness for sexual intercourse
  ○ Sexual and reproductive health indicators for younger adolescents (body pride, comfort with one's sexuality, puberty)
  ○ Sleep
• Domain 3: Policies, programmes, and laws
  ○ Policy implementation and impact
  ○ Adolescents' right to health and healthcare
  ○ Protection from harmful marketing
• Domain 4: Systems performance and interventions
  ○ Existence of routinely administered adolescent-specialized survey
  ○ Health service quality
  ○ Implementation for standalone and/or integrated services
  ○ Integration of adolescent-friendly services within primary health care system
  ○ Intersectoral coordination between health and other sectors
  ○ Prevention activities
  ○ Provision of adolescent-friendly services
  ○ Transition from period of paediatric care to adult care
• Domain 5: Subjective well-being
  ○ Measures of positive well-being
  ○ Positive youth development
  ○ Resilience, protective factors, supportive assets
  ○ Safety
  ○ Social connectedness, inclusion
• Domain 6: Health outcomes and conditions
  ○ Adolescent pregnancy
  ○ Disability
  ○ Emotional or psychological violence
  ○ Overall measure of mental health
  ○ Pain
  ○ Self-rated health
  ○ Stress, coping behaviors

Note: Some documented measurement gaps partially overlap with proposed priority indicators, such as the measurement gap on sexual and reproductive health indicators for younger adolescents and the priority indicator on the awareness of menstruation prior to menarche. In such cases, these items persist as measurement gaps because they are not completely addressed by existing indicators.

## Discussion

This paper describes a structured process to select a priority set of adolescent health indicators for use at the country and global levels. It builds on previous work of the GAMA AG to define a set of core measurement areas for adolescent health and map their related indicators. [8,12] The results of this indicator selection represent an important milestone to focus adolescent health measurement on the most important issues. With inclusion of clear metadata for each indicator, these indicators also provide a platform for promoting global harmonization of adolescent health measurement.

The indicator selection by the GAMA AG was based on an inclusive and participatory process, aimed at increasing buy-in of all relevant measurement stakeholders. At each step, inputs were collected from a broad range of stakeholders globally, including country-level measurement focal persons, adolescent health experts, partners from relevant measurement groups, and initiatives (e.g., focal persons of both global school surveys, the Global School-based Student Health Survey [25] and the Health Behavior in School-aged Children, [26]) UN partners, and others.

In addition, inputs from young people under the age of 30 featured throughout the process, who, although exceeded the defined adolescent age range, contributed an important, younger stakeholder perspective. Their feedback was combined with inputs from three other sources (i.e., country priorities, disease burden, and existing measurement efforts) when identifying the priority measurement areas for adolescent health [12] and was further included as part of the online feedback survey, the targeted distribution of which included youth organizations among other stakeholder groups.

The indicators presented here were purposefully selected to assess the most important adolescent health measurement areas, resulting in a list that is both focused yet broad in its scope. The 52 priority indicators recommended by the GAMA AG collectively assess 32 of the 33 previously identified core measurement areas for adolescent health. [12] This greatly improves on the scope of the 16 previously identified adolescent measurement initiatives, the most comprehensive of which was found to include indicators assessing 22 of these core measurement areas. [8] In addition, the priority indicators cover seven of the eight core measurement areas that were least represented among existing measurement initiatives, [8] providing an opportunity to focus efforts on measurement areas that have been historically neglected and underrepresented.

This process is not without limitations. First, the indicator list is skewed towards adolescent health indicators included in the previously identified 16 regional and global measurement initiatives. [8] While this excluded national measurement initiatives and may have caused the omission of relevant indicators from those or other initiatives at the initial stage of indicator mapping, the online feedback survey and discussions that followed provided opportunities where other indicators could be proposed for consideration. Several proposed indicators were added through this mechanism, such as two policy indicators currently measured through the WHO Sexual, Reproductive, Maternal, Newborn, Child and Adolescent Health Policy Survey. [27] Second, the selection process relied heavily on indicators that are currently in use, which offers the advantage of building on existing data systems but limits the ability to fill gaps in areas that are not currently well measured. Measurement gaps identified in this process, such as indicators assessing the sexual and reproductive health of younger adolescents, were combined to form the list presented within this article (Box 3) and provide a foundation from which further indicator development efforts can seek to address these gaps. Finally, representation of the different WHO regions and age groups regarding participation in the selection process was not even. The relatively weak representation from the WHO Western Pacific Region and from participants below 30 years of age might have influenced indicator selection towards those deemed important by other regions and older age groups.

While the selection of the current priority indicators by the GAMA AG represents a significant milestone, it has always been the intention that these indicators should be a ‘living’ list. Going forward, the list will be reviewed regularly and updated with new experience and evidence as it emerges. The current list of priority indicators and their accompanying measurement guidance will be tested for feasibility in countries. The feasibility study will be carried out in all WHO regions and will focus on availability, quality and use of data for the indicators, as well as key considerations related to their implementation. In parallel, the GAMA AG will liaise with stakeholders in the measurement community to further review current indicator recommendations and help harmonize measurement efforts across the adolescent health landscape. Separately, additional research is needed both to help fill the measurement gaps documented here and to explore innovative ways to improve current measurement practices. For example, data systems should explore if alternatives to population-based surveys might provide a reliable substitute for indicators traditionally relying on survey data.

### Conclusion

Adolescence is a critical period in the life course, but measures of adolescent health have been inconsistent and poorly defined. The priority indicators recommended by the GAMA AG provide a framework for adolescent health measurement whose scope reflects the most important issues for this population. Furthermore, metadata for these indicators were informed by best practices among current measurement initiatives. These indicators will be tested for feasibility in countries, revised if necessary, and periodically reviewed as new evidence becomes available. We call on partners to engage with this process as we collectively focus efforts on producing the most important data for adolescent health nationally and globally.
