# Supplementary material for: Priority Indicators for Adolescent Health Measurement – Recommendations From the Global Action for Measurement of Adolescent Health (GAMA) Advisory Group
Source: J Adolesc Health. 2022 Oct;71(4):455–65. doi: 10.1016/j.jadohealth.2022.04.015 (PMC9477504; doi:10.1016/j.jadohealth.2022.04.015)
Supplement: Appendix A [file mmc1.docx]

**Appendix A. Measurement initiatives and core measurement areas**

**Measurement Initiatives***

1. Global indicator framework for the Sustainable Development Goals and targets of the 2030 Agenda for Sustainable Development^1^
2. The Lancet Commission on Adolescent health and wellbeing^2^
3. Indicator and Monitoring Framework for the Global Strategy for Women’s, Children’s, and Adolescents’ Health (2016-2030)^3^
4. Countdown to 2030^4^
5. Family Planning 2020^†5^
6. Adolescent Country Tracker^6^
7. Global Reference List of 100 Core Health Indicators^7^
8. Global Reference List of Health Indicators for Adolescents (aged 10-19 years)^8^
9. Core Indicators for Adolescent Health: A Regional Guide (EMRO)^9^
10. Commonwealth Youth Development Index^10^
11. INSPIRE Indicator Guidance and Results Framework^11^
12. Measurement of Mental Health among Adolescents at the Population Level (MMAP) initiative^12^
13. Monitoring and Evaluation Guidance for School Health Programmes^13^
14. Measuring the Education Sector response to HIV and AIDS: guidelines for the construction and use of core indicators^14^
15. UNECE Monitoring Framework for the ICPD Programme of Action beyond 2014^15^
16. WHO’s 13th General Programme of Work Impact Framework^16^

*Adapted from Newby, et al. (2021)^17^.

^†^Now “Family Planning 2030”

In addition to indicators identified from these 16 initiatives, the draft list of proposed indicators includes two indicators from the WHO Sexual, Reproductive, Maternal, Newborn, Child and Adolescent Health Policy Survey^18^, one indicator from the Global Information System on Alcohol and Health^19^, and one indicator from the Global Youth Tobacco Survey^20^.

**Core measurement areas, organized by domain***

- **Domain 1: Social, cultural, economic, educational, environmental determinants of health**
  - Education level and schooling status
  - Gender
  - Income level and poverty
  - Population
- **Domain 2: Health behaviours and risks**
  - Alcohol use
  - Bullying
  - Contraception
  - Dietary behaviour
  - Physical activity
  - Reproductive health
  - Sexual health
  - Substance use, other than alcohol and tobacco
  - Tobacco use
  - Weight status
- **Domain 3: Policies, programmes, and laws**
  - Adolescent health policies and plans
  - Adolescent health protective laws
- **Domain 4: Systems performance and interventions**
  - Health service availability and access
  - Health service quality
  - Immunization
  - System for monitoring and surveillance of adolescent health
- **Domain 5: Subjective well-being**
  - None
- **Domain 6: Health outcomes and conditions**
  - All-cause mortality
  - Adolescent fertility
  - Anxiety disorders
  - Cause-specific mortality
  - Depressive disorders
  - Disability
  - Gender-based violence
  - HIV/AIDS
  - Interpersonal violence
  - Road injury
  - Self-harm
  - Sexual violence
  - Sexually transmitted infections (STIs) other than HIV/AIDS

*Adapted from Guthold, et al., 2020^21^

**References**

1. United Nations. General Assembly. *A/RES/71/313. Global indicator framework for the Sustainable Development Goals and targets of the 2030 Agenda for Sustainable Development.* New York, USA: United Nations;2017.

2. Patton GC, Sawyer SM, Santelli JS, et al. Our future: a Lancet commission on adolescent health and wellbeing. *The Lancet.* 2016;387(10036):2423-2478.

3. Every Woman Every Child. *The Global Strategy for Women's, Children's and Adolescents' Health (2016-2030).* New York, NY: United Nations; 2015.

4. Countdown to 2030: tracking progress towards universal coverage for reproductive, maternal, newborn, and child health. *Lancet.* 2018;391(10129):1538-1548.

5. FP 2030. Family Planning 2030,. https://fp2030.org/. Accessed 24 March, 2022.

6. UNICEF. Adolescent Country Tracker. https://data.unicef.org/wp-content/uploads/2018/05/Adolescent-Country-Tracker-postcard-30Apr18-2.pdf. Published 2018. Accessed 24 March, 2022.

7. World Health Organization. *Global Reference List of 100 Core Health Indicators (plus health-related SDGs).* Geneva, Switzerland: World Health Organization; 2018.

8. World Health Organization. *Global Reference List of Health Indicators for Adolescents (aged 10-19 years).* Geneva, Switzerland: World Health Organization; 2015.

9. World Health Organization, Regional Office for the Eastern Mediterranean,. *Core indicators for adolescent health: a regional guide.* Cairo, Egypt: World Health Organization. Regional Office for the Eastern Mediterranean;2014.

10. The Commonwealth. The Commonwealth Youth Development Index. https://thecommonwealth.org/youthdevelopmentindex. Published 2016. Accessed 30 November, 2021.

11. United Nations Children's Fund. *INSPIRE Indicator Guidance and Results Framework - Ending Violence Against Children: How to define and measure change.* New York, USA: UNICEF;2018.

12. UNICEF. Measurement of Mental Health Among Adolescents at the Population Level (MMAP). https://data.unicef.org/topic/child-health/mental-health/mmap/. Published 2020. Accessed 24 March, 2022.

13. UNESCO. *Monitoring and Evaluation Guidane for School Health Programs.* Paris, France: UNESCO;2014.

14. UNESCO. *Measuring the education sector response to HIV and AIDS. Guidelines for the construction and use of core indicators.* Paris, France: UNESCO;2013.

15. UNECE and UNFPA. *UNECE Monitoring Framework for the ICPD Programme of Action beyond 2014.* Geneva and Istanbul: UNECE and UNFPA;2018.

16. World Health Organization. WHO 13th General Programme of Work (GPW 13) Impact Framework: Targets and indicators. World Health Organization. https://www.who.int/about/what-we-do/GPW13_WIF_Targets_and_Indicators_English.pdf. Published 2018. Accessed 24 March, 2022.

17. Newby H, Marsh AD, Moller A-B, et al. A Scoping Review of Adolescent Health Indicators. *Journal of Adolescent Health.* 2021.

18. World Health Organization. *Sexual, reproductive, maternal, newborn, child and adolescent health: policy survey, 2018-2019: summary report.* Geneva: World Health Organization; 2020.

19. World Health Organization. *Indicator Code Book - Global Information System on Alcohol and Health.* Geneva, Switzerland2014.

20. Global Youth Tobacco Survey Collaborative Group. *Global Youth Tobacco Survey (GYTS): Core Questionnaire with Optional Questions, Version 1.2.* Atlanta, GA2014.

21. Guthold R, Moller AB, Adebayo E, et al. Priority Areas for Adolescent Health Measurement. *Journal of Adolescent Health.* 2021;68(5):888-898.
