# Supplementary material for: Priority Indicators for Adolescent Health Measurement – Recommendations From the Global Action for Measurement of Adolescent Health (GAMA) Advisory Group
Source: J Adolesc Health. 2022 Oct;71(4):455–65. doi: 10.1016/j.jadohealth.2022.04.015 (PMC9477504; doi:10.1016/j.jadohealth.2022.04.015)
Supplement: Appendix B [file mmc2.docx]

**Appendix B. Online feedback survey questionnaire**

*Authors’ note: The following includes the English translation of the online feedback survey. The survey was also made available in French and Spanish, the translations of which are available from the authors upon request.*

**Welcome message**

Thank you for your interest in providing feedback on the proposed set of priority adolescent health indicators. The survey includes four sections:

1. respondent background details;
2. feedback on the framing of the proposed set of priority indicators;
3. indicator-specific feedback (including suggestions to remove, revise, or replace specific indicators);
4. overall feedback on the set of proposed indicators.

Please note that you may provide feedback on as many or as few indicators as you like.

We encourage you to download and review the proposed indicators before starting the survey ([Proposed indicators for global adolescent health measurement](http://www.who.int/docs/default-source/mca-documents/advisory-groups/gama/gama-list-of-indicators-draft-2-v20201020.pdf?sfvrsn=f6d00176_6)). You may also find that the background and overview sections provide useful context for understanding this work. Please note that reviewing the document may take 45-60 minutes. Please be aware that participation in this process is completely voluntary and that you may stop participating at any time. You will also be able to save your responses at any time and resume the survey later (see “Resume later” button). When completing the survey, you will also be able to navigate between completed sections and modify your responses as necessary (see “Question Index” button).

All contributions submitted through this process will be collected and consolidated by the WHO GAMA Secretariat and presented to the GAMA Advisory Group to inform final recommendations. By submitting your comments, you confirm that you understand that your responses will be shared with the WHO GAMA Secretariat.

**Section 1: Respondent details**

| # | Question | Response | |
| --- | --- | --- | --- |
| 1 | Your name (optional) | [insert response] | |
| 2 | Your sex (optional) | [insert response] | |
| 3 | Your age (optional) | <20 years |  |
|  |  | 20-29 years |  |
|  |  | 30-39 years |  |
|  |  | 40-49 years |  |
|  |  | 50-59 years |  |
|  |  | 60-69 years |  |
|  |  | >= 70 years |  |
|  |  | No answer |  |
| 4 | Your country of residence (optional) | [insert response] |  |
| 5a | What is your current employment status? (optional) | Employed |  |
|  |  | Unemployed |  |
|  |  | Student |  |
|  |  | Retired |  |
|  |  | Other: [insert response] |  |
| 5b | If “Employed”: Which of the following best describes your current employment?  (optional) | International organization |  |
|  |  | Government |  |
|  |  | Non-governmental organization or community-based organization |  |
|  |  | Academia |  |
|  |  | Donor |  |
|  |  | Other: [insert response] |  |
| 6a | Are you completing this survey on behalf of an organization or as an individual? (optional) | On behalf of an organization |  |
|  |  | As an individual |  |
| 6b | If “On behalf of an organization”: Please provide the name of the organization (optional) | [insert response] |  |

**Section 2: Feedback on the framing of the proposed set of priority indicators**

| # | Question | Response | |
| --- | --- | --- | --- |
| 1a | Consider **the scope and the purpose of the document**. Do you think they are clearly described? | Yes |  |
|  |  | No |  |
|  |  | No opinion |  |
| 1b | If “No”: How could the scope and purpose of the document be improved? | [insert response] | |
| 2a | Consider **the method followed to arrive at this list of indicators**. Do you think they are clearly described? | Yes |  |
|  |  | No |  |
|  |  | No opinion |  |
| 2b | If “No”: How could the description of the method be improved? | [insert response] | |

**Section 3.1: Selecting measurement domains to review and providing indicator-specific feedback**

| # | Question | Response | |
| --- | --- | --- | --- |
| 1 | Proposed indicators have been organized according to the six measurement domains listed below. Please select the domains for which you would like to provide feedback.  For a list of the indicators included within each domain, please see the PDF mentioned in the survey instructions: available [here](http://www.who.int/docs/default-source/mca-documents/advisory-groups/gama/gama-list-of-indicators-draft-2-v20201020.pdf?sfvrsn=f6d00176_6).  (check all that apply) | 1. Social, cultural, economic, educational, environmental determinants of adolescent health |  |
|  |  | 2. Health behaviours and risks |  |
|  |  | 3. Policies, programmes, and laws |  |
|  |  | 4. Systems performance and interventions |  |
|  |  | 5. Subjective well-being |  |
|  |  | 6. Health outcomes and conditions |  |
| The following sections will present you with the indicators listed within the domain(s) that you have selected. You may provide feedback on as many or as few of these indicators as you like.  If you wish to provide feedback on indicators listed within other domains, you may revisit this page at any time and update your selection (via the "Question Index" button located at the bottom of the screen). | | | |

*Authors’ note: Responses to the question above were used to populate the indicator-specific portion of the survey. Participants were sequentially shown all indicators within each selected domain, including a table including the currently proposed metadata for the indicator. Participants were then presented with a set of indicator-specific questions to provide a recommendation.*

*These questions follow a standard template according to the indicator classification as either core, additional, or alternative. The following three sections present the template of indicator-specific questions for each indicator classification.*

**Section 3.2: Template for core indicators**

| **#** | **Question** | **Responses** | |
| --- | --- | --- | --- |
| 1 | What do you recommend for this indicator?  (Indicate response with “x” in final column and complete questions with same colour) | A. Retain as a core indicator without changes |  |
|  |  | B. Retain as a core indicator with changes (specify below)  *CONTINUE TO QUESTIONS 2-3* |  |
|  |  | C. Remove from the list of core indicators  *CONTINUE TO QUESTION 4* |  |
|  |  | D. Replace with a similar indicator (specify below)  *CONTINUE TO QUESTIONS 5-7* |  |
| 2 | What changes do you recommend for this indicator? | [insert response] | |
| 3 | Why do you recommend these changes? | [insert response] | |
| 4 | Why do you recommend that this indicator be removed? | [insert response] | |
| 5 | What indicator do you propose to replace this indicator? | [insert response] | |
| 6 | Why do you propose this replacement? | [insert response] | |
| 7 | Please attach any supplementary information for the proposed  replacement (e.g., indicator details) (optional) | [submit when entering in online platform] | |

**Section 3.3: Template for additional indicators**

| **#** | **Question** | **Responses** | |
| --- | --- | --- | --- |
| 1 | What do you recommend for this indicator?  (Indicate response with “x” in final column and complete questions with same colour) | A. Retain as an additional indicator without changes |  |
|  |  | B. Retain as an additional indicator with changes (specify below)  *CONTINUE TO QUESTIONS 2-3* |  |
|  |  | C. Remove from the list of additional indicators  *CONTINUE TO QUESTION 4* |  |
| 2 | What changes do you recommend for this indicator? | [insert response] | |
| 3 | Why do you recommend these changes? | [insert response] | |
| 4 | Why do you recommend that this indicator be removed? | [insert response] | |

**Section 3.4: Template for alternative indicators**

| **#** | **Question** | **Responses** | |
| --- | --- | --- | --- |
| 1 | What do you recommend for this alternate indicator?  (Indicate response with “x” in final column and complete questions with same colour) | A. Retain as an alternate indicator without changes |  |
|  |  | B. Retain as an alternate indicator with changes (specify below)  *CONTINUE TO QUESTIONS 2-3* |  |
|  |  | C. Swap with current core indicator without changes (i.e., make 6.10-ALT a core indicator and 6.10 the alternate indicator) |  |
|  |  | D. Swap with current core indicator with changes (specify below) (i.e., make [this indicator] a core indicator and [the linked core indicator] the alternate indicator)  *CONTINUE TO QUESTIONS 2-3* |  |
|  |  | E. Remove alternate indicator  *CONTINUE TO QUESTION 4* |  |
| 2 | What changes do you recommend for this indicator? | [insert response] | |
| 3 | Why do you recommend these changes? | [insert response] | |
| 4 | Why do you recommend that this indicator be removed? | [insert response] | |

**Section 4.1 Overall feedback on additional indicators**

| # | Question | Response | |
| --- | --- | --- | --- |
| 1 | The indicator-specific questions asked individually about the 15 proposed additional indicators. Considering the overall balance among all proposed indicators, is there any one additional indicator that you recommend be elevated to the list of core indicators? | [insert additional indicator identifier (see below this table for a list of additional indicators)] |  |
|  |  | I do not recommend elevating any of these indicators to the list of core indicators |  |
| 2 | *If recommending to elevate an additional indicator:*  Why do you recommend that this indicator be elevated to the list of core indicators? | [insert response] |  |
| 3 | *If recommending to elevate an additional indicator:*  Do you recommend that this indicator replace a currently proposed core indicator or be included alongside the currently proposed core indicators? | Replace a currently proposed core indicator (specify below) |  |
|  |  | Include alongside the currently proposed core indicators |  |
| 4 | *If recommending that the indicator replace a currently proposed core indicator:*  Which currently proposed core indicator do you suggest that this indicator replace? | [insert additional indicator identifier (see below this table for a list of core indicators)] |  |

*Authors’ note: The indicators listed below represents the set of indicators being proposed at the time of the online feedback survey. For a list of the currently proposed indicators, see Table 2 of the manuscript or Appendix E.*

*Additional indicators*

- A1.01 Percentage of adolescents (10–19 years) not in education, employment, or training, by age group (10-14, 15-19 years) and sex
- A1.02 Percentage of adolescents (10-19 years) at the end of primary; and at the end of lower secondary achieving at least a minimum proficiency level in (i) reading and (ii) mathematics, by age group (10-14, 15-19 years) and sex
- A2.01 Prevalence of current (past 30 days) alcohol use among adolescents (10-19 years), by age group (10-14, 15-19 years) and sex
- A2.02 Percentage of adolescents (10-19 years) who usually drank sugar-sweetened beverages once per day or more during the past 30 days, by age group (10-14, 15-19 years) and sex
- A2.03 Percentage of female adolescents (10-19 years) who were aware of menstruation before menarche, by age group (10-14, 15-19 years)
- A3.01 Existence of national policy exempting adolescents (10-19 years) from user fees for specified health services in the public sector, by type of service
- A3.02 Existence of legal age limit for married and unmarried adolescents (10-19 years) to provide consent, without spousal/parental/legal guardian consent, for specified adolescent health services, by marital status and type of service
- A4.01 Existence of a nationally-defined minimum package of school-based health and nutrition services based on local health priorities
- A4.02 Percentage of schools that provided life skills-based HIV and sexuality education within the previous academic year
- A5.01 Percentage of adolescents (10-19 years) with someone to talk to when they have a worry or problem, by age group (10-14, 15-19 years) and sex
- A5.02 Percentage of adolescents (10-19 years) with a positive connection with their parent or guardian, by age group (10-14, 15-19 years) and sex
- A6.01 Incidence rate of sexually transmitted infections (STIs) among adolescents (10-19 years), by age group (10-14, 15-19 years) and sex
- A6.02 Percentage of adolescents (10-19 years) reporting current (past two weeks) suicidal thoughts, by age group (10-14, 15-19 years) and sex
- A6.03 Percentage of female adolescents (10-19 years) who have undergone female genital mutilation/cutting, by age group (10-14, 15-19 years)
- A6.04 Prevalence of anaemia among adolescents (10-19 years), by age category (10-14, 15-19 years) and sex

Core indicators

- Percentage of total population that are adolescents (10–19 years), by age category (10-14, 15-19 years) and sex
- 1.02 Percentage of adolescents completing primary, lower secondary, and upper secondary school, by level and sex
- 1.03 Percentage of adolescents (10-19 years) living below the national poverty line, by age group (10-14, 15-19 years) and sex
- 1.04 Percentage of adolescents (10-19 years) living with moderate or severe food insecurity in the population, based on the Food Insecurity Experience Scale (FIES), by age group (10-14, 15-19 years) and sex
- 1.05 Percentage of female adolescents (15-19 years) who make their own informed decisions regarding sexual relations, contraceptive use and reproductive health care
- 2.01 Prevalence of overweight and obesity among adolescents (10-19 years), by weight status (overweight, obese), age group (10-14, 15-19 years), and sex
- 2.02 Prevalence of thinness among adolescents (10-19 years), by age group (10-14, 15-19 years), and sex
- 2.03 Past 30 day prevalence of heavy episodic drinking among adolescents (10-19 years), age group (10-14, 15-19 years) and sex
- 2.04 Past 12 month prevalence of psychoactive drug use among adolescents (10–19 years), by age group (10-14, 15-19 years), sex and by type of substances
- 2.05 Prevalence of current (past 30 days) use of tobacco products among adolescents (10–19 years), by age group (10-14, 15-19 years), sex, and type of tobacco used
- 2.06 Percentage of adolescents (10–19 years) who consume at least 5 servings of fruit and vegetables daily, by age group (10-14, 15-19 years) and sex
- 2.07 Percentage of adolescents (10–19 years) who have accumulated an average of at least 60 minutes per day of moderate‐vigorous physical activity in the previous week, by age group (10-14, 15-19 years) and sex
- 2.08 Percentage of adolescents (10-19 years) involved in bullying within the past 12 months, by type of involvement (victim, perpetrator, both), type of bullying (in-person, digital/cyber), age group (10-14, 15-19 years), and sex
- 2.09 Percentage of adolescents (15-19 years) who had their first sexual intercourse before 15 years of age, by sex
- 2.10 Percentage of live births to female adolescents (10-19 years) attended by skilled health personnel, by age group (10-14, 15-19 years)
- 2.11 Prevalence of contraceptive use (modern method) among female adolescents (10-19 years), by age group (10-14, 15-19 years) and method used
- 2.12 Percentage of female adolescents (10-19 years) who have their need for contraception satisfied with modern methods, by age group (10-14, 15-19 years)
- 3.01 Existence of a functional adolescent (10-19 years) health program with coverage at the national level
- 3.02 Existence of national standards for delivery of health services to adolescents (10-19 years)
- 4.01 Percentage of adolescents (10-19 years) using specified health services within the past 12 months, by age group (10-14, 15-19 years) and sex
- 4.02 Percentage of adolescents (15 years) covered by HPV vaccine (last dose in schedule), by sex
- 4.03 Existence of age‐ and sex‐disaggregated health data for adolescents (10-19 years) in the national health information system
- 6.01 Adolescent (10-19 years) mortality rate, by age group (10-14, 15-19 years) and sex
- 6.02 Adolescent (10-19 years) mortality rate, by specified causes of death, age group (10-14, 15-19 years) and sex
- 6.03 Number of new adolescent (10-19 years) HIV infections per 1,000 uninfected adolescent population, by age group (10-14, 15-19 years) and sex
- 6.04 Percentage of adolescents (10-19 years) who used a condom at last intercourse, by age group (10-14, 15-19 years) and sex
- 6.05 Percentage of adolescents (10-19 years) reporting a suicide attempt in the past 12 months, by age group (10-14, 15-19 years) and sex
- 6.06 Percentage of adolescents (10-19 years) with depression and/or anxiety, by age group (10-14, 15-19 years) and sex
- 6.07 Percentage of adolescents (10-19 years) with depression and/or anxiety seeking mental health care or psychosocial support, by age group (10-14, 15-19 years) and sex
- 6.08 Incidence rate of specified types of injuries among adolescents (10–19 years), and by age category (10-14, 15-19 years), sex and type of injuries (per 100,000 population)
- 6.09 Percentage of adolescents (10-19 years) involved in physical violence in the past 12 months, by type of involvement (victim, perpetrator, both), age group (10-14, 15-19 years), sex, perpetrator (parents/caregivers, teachers, intimate partners, peers)
- 6.10 Percentage of adolescents (10-19 years) experiencing contact sexual violence in the past 12 months, by age group (10-14, 15-19 years), sex, and perpetrator
- 6.11 Adolescent (10-19 years) fertility rate, by age group (10-14, 15-19 years)

**Section 4.2 Measurement gaps**

| # | Question | Response | |
| --- | --- | --- | --- |
| 1 | As part of this process, the following measurement gaps have been identified. These represent areas where further development is required to identify a suitable indicator.  Please select UP TO THREE measurement gaps that you would prioritize for indicator development. You may also select “Other” and enter an item not appearing on the list. (optional) | Youth homelessness |  |
|  |  | Gender identity |  |
|  |  | Active travel |  |
|  |  | Sports participation |  |
|  |  | Online behaviours |  |
|  |  | Addictive behaviours, particularly gaming disorder |  |
|  |  | Pre-coital sexual activity |  |
|  |  | SRH indicators for younger adolescents (body pride, comfort with one’s sexuality, puberty, menstruation/menstrual health) |  |
|  |  | Abortion and post-abortion care |  |
|  |  | Counselling bias for contraception methods (e.g., adolescents not counselled on all  methods, such as emergency contraception) |  |
|  |  | Overall indicator assessing adolescent right to health/healthcare |  |
|  |  | Measures of policy implementation and impact |  |
|  |  | Prevention activities |  |
|  |  | Measures of health service quality |  |
|  |  | Proportion of health facilities providing adolescent-friendly services |  |
|  |  | Integration of adolescent-friendly services within primary health care system |  |
|  |  | Indicator of implementation for standalone and/or integrated services |  |
|  |  | Existence of routinely administered adolescent-specialized survey |  |
|  |  | Measures of positive well-being |  |
|  |  | Resilience, protective factors, supportive assets |  |
|  |  | Positive youth development |  |
|  |  | Sleep |  |
|  |  | Self-rated health |  |
|  |  | Other: [insert response] |  |

**Section 4.3 Overall feedback**

| # | Question | Response | |
| --- | --- | --- | --- |
| 1 | Do the proposed indicators cover the key issues and emerging priorities in adolescent health? | Yes |  |
|  |  | No (specify below) |  |
|  |  | No opinion |  |
| 2 | *If “No”:*  What key issues and/or emerging priorities are not covered? | [insert response] | |
| 3 | Consider the six adolescent health measurement domains presented in  Table 1 of the document:  1. Social, cultural, economic, educational, environmental  determinants of health  2. Health behaviours and risks  3. Policies, programmes, and laws  4. Systems performance and interventions  5. Subjective well-being  6. Health outcomes and conditions  How balanced are the proposed indicators with respect to these six domains? | Highly balanced |  |
|  |  | Somewhat balanced |  |
|  |  | Neither balanced nor imbalanced |  |
|  |  | Somewhat imbalanced |  |
|  |  | Highly imbalanced |  |
|  |  | No opinion |  |
| 4 | Please include any comments on the overall balance of indicators by domain (optional) | [insert response] | |
| 5 | Consider that the proposed listed includes 33 CORE indicators. How would you describe the number of CORE indicators? | The number seems appropriate |  |
|  |  | Too many core indicators are proposed |  |
|  |  | Too few core indicators are proposed |  |
|  |  | No opinion |  |
| 6 | Consider that the proposed listed includes 15 ADDITIONAL indicators. How would you describe the number of ADDITIONAL indicators? | The number seems appropriate |  |
|  |  | Too many additional indicators are proposed |  |
|  |  | Too few additional indicators are proposed |  |
|  |  | No opinion |  |
| 7 | Please use this space to provide any additional comments on the proposed indicators (optional) | [insert response] | |
