# Supplementary material for: Priority Indicators for Adolescent Health Measurement – Recommendations From the Global Action for Measurement of Adolescent Health (GAMA) Advisory Group
Source: J Adolesc Health. 2022 Oct;71(4):455–65. doi: 10.1016/j.jadohealth.2022.04.015 (PMC9477504; doi:10.1016/j.jadohealth.2022.04.015)
Supplement: Appendix C [file mmc3.docx]

**Appendix C. Participant involvement in indicator selection process**

Participants were from 67 WHO Member States across all WHO regions, including 64 participants (25%) located in the WHO African Region, 61 (23%) in the Region of the Americas, 28 (11%) in the Eastern Mediterranean Region, 78 (30%) in the European Region, 17 (7%) in the South-East Asian Region, and 11 (4%) in the Western Pacific Region (Table C1).

**Table C1. Participant involvement by WHO Regions and Member States**

| **WHO region** | **Individual participants**  **N (%)** | **Member States represented**  **N (%)^1^** |
| --- | --- | --- |
| AFR | 64 (25%) | 18 (38%) |
| AMR | 61 (23%) | 11 (31%) |
| EMR | 28 (11%) | 12 (57%) |
| EUR | 78 (30%) | 16 (30%) |
| SEAR | 17 (7%) | 6 (55%) |
| WPR | 11 (4%) | 4 (15%) |
| Other/not specified | 2 (1%) | - |
| Total | 261 (100%) | 67 (35%) |

^1^ This column shows the number of unique Member States reported by participants and the proportion that this represents of the total number of Member States in the same area.

Abbreviations: AFR = WHO African Region; AMR = WHO Region of the Americas; EMR = WHO Eastern Mediterranean Region; EUR = WHO European Region; SEAR = WHO South-East Asia Region; WPR = WHO Western Pacific Region

**Table C2. Participant groups represented in the 3^rd^ through 6^th^ GAMA Meetings**

|  | **3^rd^ GAMA Meeting**  **(February 2020, Cape Town)** | **4^th^ GAMA Meeting**  **(June 2020, virtual)** | **5^th^ GAMA Meeting**  **(December 2020, virtual)** | **6^th^ GAMA Meeting**  **(April 2021, virtual)** |
| --- | --- | --- | --- | --- |
| GAMA advisory group and Secretariat | X | X | X | X |
| Representatives of UN organizations | X | X | X | X |
| Country representatives | X | X |  | X |
| ADH focal points of WHO regional and country offices | X* | X |  | X |
| Representatives of WHO HQ technical departments |  | X |  | X |
| Observers | X | X | X | X |

***ADH focal points from AFR only**

Note: See Appendix D for a summary of participant involvement in the online feedback survey.
