# Supplementary material for: Priority Indicators for Adolescent Health Measurement – Recommendations From the Global Action for Measurement of Adolescent Health (GAMA) Advisory Group
Source: J Adolesc Health. 2022 Oct;71(4):455–65. doi: 10.1016/j.jadohealth.2022.04.015 (PMC9477504; doi:10.1016/j.jadohealth.2022.04.015)
Supplement: Appendix D [file mmc4.docx]

**Appendix D. Summary of participant involvement in online feedback survey**

**Figure D1. Timeline of online feedback survey**


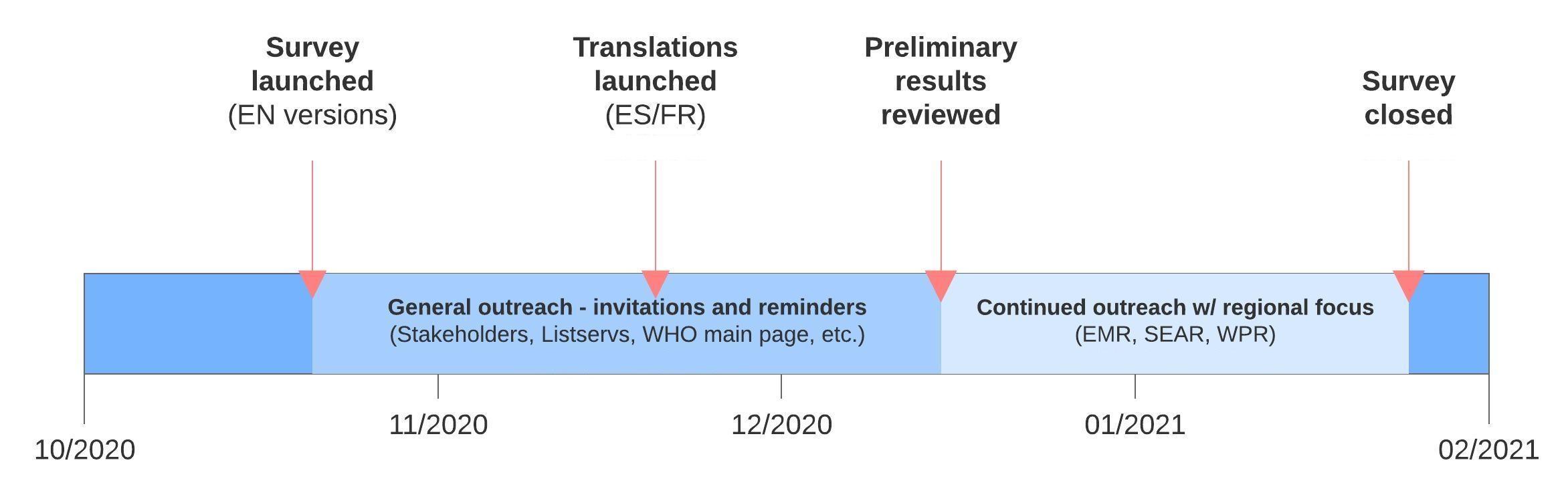


- General outreach: E-mails to key stakeholders, country missions, and relevant listservs; social media via Twitter (MCA, PMNCH, IBP Network); WHO main page and WHO newsroom article.
- Continued outreach with regional focus: Targeted regions with lower participation; Distribution by WHO regional ADH focal points and regional GAMA members

**Figure D2. Summary of survey participation**

- 156 individuals participated in the survey


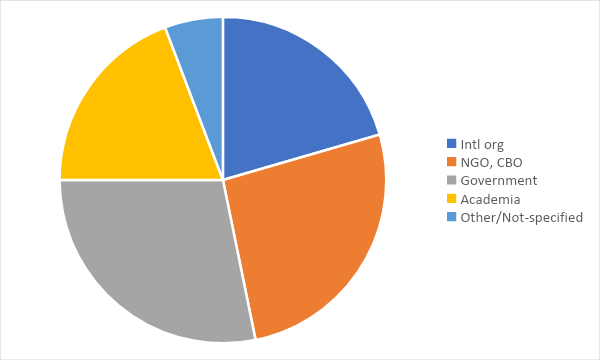


| **Table D1. Survey participation by age group** | |
| --- | --- |
| **Age group** | **Submissions (%)** |
| < 20 years | 0 (0%) |
| 20-29 years | 12 (8%) |
| 30-39 years | 25 (16%) |
| 40-49 years | 42 (27%) |
| 50-59 years | 29 (19%) |
| 60-69 years | 13 (8%) |
| >= 70 years | 2 (1%) |
| Not reported | 33 (21%) |
| Total | 156 (100%) |

| **Table D2. Survey participation by WHO region** | | |
| --- | --- | --- |
| **Region** | **Submissions (%)** | **Countries**  **represented** |
| AFR | 39 (25%) | 17 |
| AMR | 34 (22%) | 11 |
| EMR | 25 (16%) | 11 |
| EUR | 36 (23%) | 14 |
| SEAR | 13 (8%) | 6 |
| WPR | 7 (4%) | 3 |
| Not specified | 2 (1%) | N/A |
| Total | 156 (100%) | 62 |

AFR = WHO African Region; AMR = WHO Region of the Americas; EMR = WHO Eastern Mediterranean Region; EUR = WHO European Region; SEAR = WHO South-East Asia Region; WPR = WHO Western Pacific Region

**A4.3 Overview of indicator-specific feedback**

**
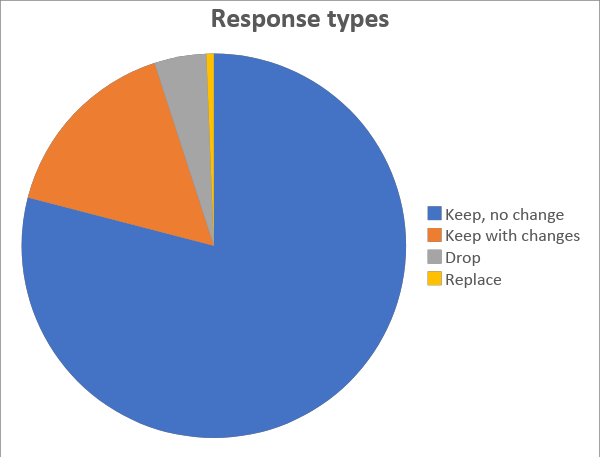
**

- Approach
  - Participants selected which indicators to review within each domain
  - Each indicator included MCQ for main recommendation with text box for additional detail
  - Keep, no change; Keep with changes; Drop; Replace
- Results
  - Indicators were reviewed by an average of 35 participants each
  - Almost 80% of responses were “keep as is”
  - Next most common was “keep with changes” (16%)

**A4.4 Overall feedback on balance of indicators**

- Most participants (86%) responded that indicators were either highly or somewhat balanced among 6 domains
- Additional comments noted some domains/content areas that may be under- and over-represented
- **Under-represented:**
  - Policies, programmes, laws (x4); Well-being (x3); Mental health (x2); Health systems and interventions (x1)
- **Over-represented:**
  - Sexual and reproductive health (x5); Generally, consider reducing overlap in specific area
