# Supplementary material for: Priority Indicators for Adolescent Health Measurement – Recommendations From the Global Action for Measurement of Adolescent Health (GAMA) Advisory Group
Source: J Adolesc Health. 2022 Oct;71(4):455–65. doi: 10.1016/j.jadohealth.2022.04.015 (PMC9477504; doi:10.1016/j.jadohealth.2022.04.015)
Supplement: Appendix E [file mmc5.pdf]

Priority indicators for adolescent health measurement proposed by the Global Action for Measurement of Adolescent health (GAMA) Advisory Group

DRAFT FOR FEASIBILITY STUDY

Version 21 June 2022

World Health Organization

Disclaimer: This document is a draft to be used for country-level feasibility testing. Results of the feasibility study will inform a final round of revisions, which will be undertaken by the WHO GAMA Secretariat in consultation with the members of the GAMA Advisory Group.

DRAFT

Table of contents

Tables and figures 5

List of acronyms and abbreviations 6

Background 8

The Global Action for Measurement of Adolescent health (GAMA) 8

Purpose of this priority indicator list 8

Methods to derive the proposed indicator list 9

Step 1. Mapping of adolescent health measurement initiatives and indicator lists 9

Step 2. Selection of priority adolescent health measurement areas 9

Step 3. Mapping of indicators assessing the core adolescent health measurement areas 11

Step 4. Developing selection criteria for prioritizing mapped indicators 11

Step 5. Prioritizing indicators and developing a first draft of the list of proposed priority indicators 12

Step 6. Internal review and revision of proposed priority indicators 12

Step 7. Public feedback and subsequent revisions 13

Next steps 14

Indicator information and important considerations 15

Indicator types and numbering 15

Indicator disaggregation 15

Measurement gaps 16

Structure of the indicator tables 16

Overview and summary table of proposed indicators 16

Indicator details 16

Overview and summary table of proposed indicators 18

Indicator details 31

1. Social, cultural, economic, educational, and environmental determinants of health 31

2. Health behaviours and risks 41

3. Policies, programmes, and laws 64

4. Systems performance and interventions 69

5. Subjective well-being 75

DRAFT

6. Health outcomes and conditions 78
References 97
Annexes 100
Annex 1: Additional details on methods 100
Annex 2: Adolescent health measurement domains, core measurement areas, and contents area 101
Annex 3: Classification of adolescent health measurement areas 102

DRAFT

Tables and figure

Table 1. Selected core, expanded and context-specific adolescent health measurement areas under six domains 10

Table 2. Distribution of core, alternative, and additional indicators by measurement domain 18

Figure 1. Preferred data sources for all proposed indicators by measurement domain 19

Table 3. Summary of proposed priority indicators 20

DRAFT

List of acronyms and abbreviations

AA-HA!	Global Accelerated Action for the Health of Adolescents
AG	advisory group
AIDS	Acquired Immune Deficiency Syndrome
BMI	body mass index
CRVS	civil registration and vital statistics
DSM-5	Diagnostic and Statistical Manual of Mental Disorders, 5th Edition
EMRO	WHO Regional Office for the Eastern Mediterranean
FGM	female genital mutilation
FIES	Food Insecurity Experience Scale
FRESH	Focusing Resources on Effective School Health
GAMA	Global Action for the Measurement of Adolescent Health
GPW 13	13th General Programme of Work (WHO)
HIV	human immunodeficiency virus
HMIS	health management information system
HPV	human papilloma virus
ICD-11	International Classification of Diseases
ICPD	International conference on population and development
MMAP	Measurement of Mental Health Among Adolescents at the Population Level
SD	standard deviation
SDG	Sustainable Development Goal
SRMNCAH	sexual, reproductive, maternal, newborn, child, and adolescent health
STI	sexually transmitted infection
UN	United Nations
UNECE	United Nations Economic Commission for Europe
UNFPA	United Nations Population Fund
UNICEF	United Nations Children's Fund
WHO	World Health Organization

DRAFT

Acknowledgement

The current draft of this document would not be possible without the meaningful contributions of the many who have assisted in this process both directly and indirectly. We would like to thank the organizations who have invested substantial resources in the development and implementation in the adolescent health measurement initiatives and indicator lists that formed the basis for the indicators included herein. These inputs provided the invaluable foundation upon which this work was built. Next, we would like to thank the numerous individuals involved in the selection and refining of indicators from these existing sources to produce a set of adolescent health indicators with broad coverage of the key areas of adolescent health. Lastly, we would like to thank the Bill and Melinda Gates Foundation, the generous support from which enabled this work to take place.

DRAFT

Background

The Global Action for Measurement of Adolescent health (GAMA)

To improve adolescent health measurement globally, the World Health Organization (WHO), in collaboration with other United Nations (UN) agencies, including the Joint UN Program on human immunodeficiency virus (HIV) and Acquired Immune Deficiency Syndrome (AIDS), UN Educational, Scientific, and Cultural Organization, UN Population Fund (UNFPA), UN Children's Fund (UNICEF), UN Women, the World Bank Group, and the World Food Programme, established the Global Action for Measurement of Adolescent health (GAMA) advisory group (AG) in 2018.

The GAMA AG consists of 16 members, including 12 senior and four young professionals, from 12 countries across all WHO Regions. Members were selected through a competitive process following an open call, based on their technical expertise and ensuring sex and geographic balance, and coverage of the main health issues for adolescents across the group.

The target population for GAMA's work includes younger (10-14 years) and older (15-19 years) adolescents of all genders, adolescents in and out of school, in humanitarian settings, adolescents living with disabilities, ethnic and religious minorities, migrants, and institutionalised adolescents.

The objectives of GAMA's work are:

- To provide technical guidance to WHO, partner UN agencies and other relevant measurement groups **to define a set of priority adolescent health indicators**, for the purpose of harmonizing efforts around adolescent health measurement and reporting;
- To promote harmonized guidance for adolescent health measurement that supports countries and technical organizations in the collection of useful data to track progress in the improvement of adolescent health.

For additional information on GAMA, please see GAMA's website[1] and a related commentary.[2]

Purpose of this priority indicator list

This document details a list of priority indicators that are proposed for the measurement of adolescent health in all countries and all adolescent population subgroups. The number of included indicators has been purposefully limited to minimize the reporting burden and ensure that the final product is feasible to measure at the country level. By definition, the indicators contained within this list are not intended to provide a comprehensive assessment of all areas of adolescent health. Rather, this list represents a set of priority indicators selected to be

applicable to adolescents globally. The indicators presented here are intended to guide policy and programming and to assist with identifying areas where further and more detailed assessments of health status and programming are needed.

Methods to derive the proposed indicator list

The selection of the indicators included in this document builds upon several earlier steps completed by GAMA. A summary of these steps is included below.

Step 1. Mapping of adolescent health measurement initiatives and indicator lists

A total of 16 global or regional adolescent health measurement initiatives and indicator lists were identified through an expert consultation that included members of the GAMA AG UN representatives, focal points from WHO Departments, and focal points from WHO Regional Offices. Included initiatives had to meet three criteria:

(1) include recommendations about adolescent health measurement;
(2) propose at least one indicator specifically including “adolescent”, “youth” or “young people”, or including the entire or part of the adolescent age range 10-19 years; and
(3) be globally or regionally (i.e., multi-country) focused.

The list of initiatives is included in Annex 1.

Step 2. Selection of priority adolescent health measurement areas

As a first step towards selecting priority indicators, priority areas for adolescent health measurement were identified. To do this in an informed and systematic way, GAMA collected four critical inputs to inform the priority setting: (1) young people’s perspectives; (2) priorities in countries; (3) the adolescent disease burden; and (4) measurement areas included in the 16 initiatives mentioned in Step 1. The selection of priority areas is described in detail in a published manuscript.[3]

In brief, a total of 99 health areas were identified, mapped across the four inputs, and grouped under six domains forming an adolescent health measurement framework. Each health area was coded according to the frequency in each of the four inputs. From this, using a modified Delphi approach, the members of the GAMA AG then selected:

- 33 core measurement areas (considered by more than 70% of GAMA AG members to be relevant to all adolescents globally);
- 19 expanded measurement areas (considered by 50-70% of GAMA AG members to be relevant to all adolescents globally);
- And six context-specific measurement areas (considered by more than 70% of GAMA AG members to be relevant only to adolescents of specific regions and/or specific subgroups).

For additional details on the classification of measurement areas, see Table 1 and Annex 3.

Table 1. Core, expanded and context-specific adolescent health measurement areas under six domains

Core adolescent health measurement areas	Expanded adolescent health measurement areas	Context-specific adolescent health measurement areas
**Domain 1: Social, cultural, economic, educational, and environmental determinants of health**		
Education level and schooling status	Being part of a vulnerable group	Child marriage
Gender	Environment and pollution	Child labour
Income level and poverty	Ethnicity	Social and cultural norms
Population	Social support	
**Domain 2: Health behaviours and risks**		
Alcohol use	Sedentary behaviour	
Bullying	Sleep	
Contraception	Social media and internet	
Dietary behaviour		
Physical activity		
Reproductive health		
Sexual health		
Substance use, other than alcohol and tobacco		
Tobacco use		
Weight status		
**Domain 3: Policies, programmes, and laws**		
Adolescent health policies and plans	Adolescent health programmes	
Adolescent health protective laws	Adolescents' participation in programming and planning	
**Domain 4: Systems performance and interventions**		
Health service availability and access	Health education	Social protection
Health service quality	Health service utilization and barriers	
Immunization	School health	
System for monitoring and surveillance of adolescent health		
**Domain 5: Subjective well-being**		
	Autonomy	
	Social connectedness	
**Domain 6: Health outcomes and conditions**		
All-cause mortality	Asthma	Iron-deficiency

DRAFT

Core adolescent health measurement areas	Expanded adolescent health measurement areas	Context-specific adolescent health measurement areas
Adolescent fertility	Collective violence and legal intervention	Vitamin A deficiency
Anxiety disorders	Diabetes	
Cause-specific mortality	Drowning	
Depressive disorders	Maternal conditions	
Disability		
Gender-based violence		
HIV/AIDS		
Interpersonal violence		
Road injury		
Self-harm		
Sexual violence		
Sexually transmitted infections (STIs) other than HIV/AIDS		

Step 3. Mapping of indicators assessing the core adolescent health measurement areas

The 16 measurement initiatives identified in Step 1 were reviewed against the 33 core measurement areas identified in Step 2, while expanded and context-specific measurement areas will be addressed later. All indicators addressing at least one of the 33 core measurement areas were extracted, producing a list of 413 adolescent health indicators.[4] Extracted indicator metadata included indicator name, definition, numerator, denominator, type, relevant age group, use status, data source, and the measurement initiative where the indicator was included. Indicators were further classified according to the core measurement areas that they addressed.

Step 4. Developing selection criteria for prioritizing mapped indicators

Next, the GAMA AG defined selection criteria for selecting priority indicators from among those identified in the indicator mapping. Initial selection criteria were discussed at the 2nd GAMA Meeting (June 2019, Geneva) and were further refined and their use was pilot tested during the 3rd GAMA Meeting (February 2020, Cape Town). The final list included four criteria: ***Relevance, Feasibility, Validity,*** and ***Usefulness***. For further details on the selection criteria, see Annex 1.

DRAFT

Step 5. Prioritizing indicators and developing a first draft of the list of proposed priority indicators

To facilitate the process of subsequent indicator selection, the 413 indicators mapped in Step 3 were organized into eight content areas based on the adolescent health measurement domains and the core measurement area they assessed (Annex 2). A working group was formed for each of the eight content areas, the membership of which included GAMA AG members, UN representatives, and country representatives attending the 3rd GAMA Meeting. Each group member was given an Excel sheet with the indicators specific to his or her group and proceeded to score each indicator according to each of the criteria defined in Step 4 on a 5-point scale. A summary score for each indicator was generated as the average score across each criterion and each participant.

During virtual meetings among members of each of the eight groups, the scoring results of the top ten indicators were reviewed and group members proposed priority, alternative, and additional indicators.

Following the working group virtual meetings and based on the proposed priority, alternative, and additional indicators, the WHO GAMA Secretariat developed a first draft list of proposed priority indicators.

Step 6. Internal review and revision of proposed priority indicators

The first draft list of proposed priority indicators was circulated among participants in advance of the 4th GAMA Meeting (June 2020, virtual meeting). They included members of the GAMA AG; GAMA representatives across WHO technical departments, WHO Regional Offices, and UN partner agencies; country representatives attending the 3rd GAMA Meeting; and observers. During the meeting, proposed indicators were reviewed according to their core measurement area. Participants noted where revisions to proposed indicators would be helpful and identified potential measurement gaps.

Based on all inputs received before and during the 4th GAMA Meeting, a second draft list of proposed adolescent health indicators was compiled and circulated for another review by meeting participants. All feedback was reviewed by the WHO GAMA Secretariat and the document was revised accordingly, resulting in a third draft of the list of proposed priority indicators.

DRAFT

Step 7. Public feedback and subsequent revisions

The third draft of the list of proposed indicators for adolescent health measurement was placed online for public review and comment. The document was available in English, French, and Spanish. Public feedback was collected through a structured online survey hosted through DataForm, a WHO version of the LimeSurvey platform.[5] The survey included four sections:

1. respondent background details;
2. feedback on the framing of the proposed set of priority indicators;
3. indicator-specific feedback (including suggestions to remove, revise, or replace specific indicators); and
4. overall feedback on the set of proposed indicators.

Invitations to participate were sent to key stakeholders, including through e-mails distributed by WHO's Governing Bodies Department to country Missions of WHO Member States according to the "Official List of Missions", experts in adolescent health and measurement, and relevant listservs. The feedback process was also advertised through social media (Twitter), a WHO Newsroom article, and featured on the WHO main webpage.

The English version of the survey was launched in October 2020 with French and Spanish versions of the survey available the following month. Preliminary survey results were reviewed during the 5th GAMA Meeting (December 2020, virtual meeting), and it was decided that the survey would remain open through mid-January 2021. The additional time was used to conduct targeted outreach to regions that were under-represented in the first review of responses. In total, more than 150 participants across all world regions provided feedback through the online survey.

All feedback was reviewed and considered when preparing the fourth draft of the proposed list of indicators, which was presented at the 6th GAMA Meeting (April 2021, virtual meeting). The discussion included a review of major changes and outstanding issues. A summary of the discussion including participant recommendations was compiled and shared with members of the GAMA AG for their final consideration.

As the last step in the review process, the GAMA AG conducted a final review of the proposed indicators. This review focused on the pending issues raised at the 6th GAMA Meeting and the revisions proposed by the WHO GAMA Secretariat in response to the online feedback survey. The latest version of the indicator guidance document was uploaded to Google Docs for collaborative review. To allow for a more in-depth review, GAMA AG members organized themselves into working groups for each of the six adolescent health measurement domains. Working group members collaboratively reviewed the indicators within their selected domain and group-specific virtual meetings were held to address any issues where further discussion

was required. Based on these final inputs, the WHO GAMA Secretariat revised the list of proposed indicators to its final draft.

Next steps

The process described above marks the end of the development for the first set of priority indicators for adolescent health. The next steps for these indicators involve two streams of work. First, desk-based reviews of data sources and availability will be conducted for each proposed indicator. In terms of data availability, countries will be classified as either measuring an indicator that is an exact match, a partial match, or no matching indicator. Where an exact match exists, recent data for the indicator will also be compiled. Where a partial match exists, the deviations from the proposed indicator will be documented and serve as an input in efforts to promote harmonization of indicator measurement. Second, this set of indicators will be tested for feasibility in countries and will be further revised based on these experiences. Furthermore, it should be noted that these indicators are not static and will periodically be revisited and updated in accordance with new available evidence.

DRAFT

Indicator information and important considerations

Indicator types and numbering

The GAMA AG proposed three types of indicators:

- **Core indicators** are considered to be the most important for measuring the health of all adolescents globally.
- **An alternative indicator** is proposed in one case where the core indicator may not be feasible or may be too sensitive to measure.
- **Additional indicators** are provided for settings where further detail within that area would add value, and resources for data collection and reporting are available.

Each indicator is assigned a unique identifier based on the measurement domain where it is listed. The alternative indicator is listed after the indicator for which it serves as an alternative and has “ALT” appended to the number. Additional indicators are listed at the end of each content area and have “A” added to the beginning of the identifier.

Indicator disaggregation

The GAMA AG has generally proposed standard disaggregation by sex[a] and by five-year age groups (i.e., 10-14, 15-19 years) for all indicators where pertinent.[6] Disaggregation by additional characteristics is proposed for selected indicators where this is deemed particularly informative (e.g., type of tobacco used for the indicator on current tobacco use). The disaggregation proposed in the indicator-specific metadata is intended to focus on the most important characteristics, though additional disaggregation may be both beneficial and necessary in certain contexts.

The following non-exhaustive list includes potential additional characteristics to consider for disaggregation:

- General disaggregation
    - adolescent living situation (living on own, living with family, in foster care)
    - belonging to a vulnerable population (indigenous, international migrant, etc.)
    - intersectionality (race, class, gender, etc.)
    - household characteristics (family size, nuclear, extended family)
    - marital status (unmarried, married)
    - more specific age groups (individual age, finer scale age groups)
    - nutrition (food security status)
    - residence (urban/rural, peri urban, urban slum, refugee camp)

---

[a] In this context, ‘sex’ refers to biological sex at birth with primary classification as male or female. Where relevant, additional classification options may be considered. Furthermore, disaggregation by additional related constructs, such as current gender identity, may also be considered in certain contexts.

    - schooling status (education level, in-school, out-of-school)
- Sexual and reproductive health indicators
    - consent status for sexual intercourse (consensual, forced/coerced, transactional)
    - type of sexual partner
    - whether childbirth occurred in facility or elsewhere
- Substance use indicators
    - more specific tobacco types
    - most frequently used substances as per national context
- Violence indicators
    - location where violence occurred (community, school, home)
    - nature of bullying (sexual, physical, emotional [e.g., public humiliation])
    - perpetrator characteristics (age, sex)
    - relationship to perpetrator (e.g., older peer, same age peer, current or former boyfriend/girlfriend, teacher, spouse, religious figure, other adult)
    - whether motivated by victim characteristic (e.g., sexist/misogynist, homophobic/transphobic, disability status)

Measurement gaps

Participants in each of the group-specific virtual meetings, the 4th GAMA Meeting, and the internal review of the first draft of this document were asked to identify any measurement gaps within the currently proposed set of indicators. Those measurement gaps relating to one of the 33 previously defined core measurement areas (Table 1) where no suitable indicator was identified are included in this document and can be found at the end of the domain-specific section summaries.

Structure of the indicator tables

The remainder of this document presents the proposed priority indicators in further detail and is broadly organized into two sections:

Overview and summary table of proposed indicators

The first section includes a high-level summary of all proposed priority indicators with selected metadata information presented for each indicator (indicator name, definition, and the initiative from which it was derived). This section is intended to provide an overview of the proposed indicators and may be useful when considering the set of indicators as a whole.

Indicator details

The second section presents expanded metadata details for each proposed indicator and is organized according to the six measurement domains (Table 2). Each subsection begins with a brief summary that lists the indicators included within the subsection, relevant indicators included in another subsection (for indicators that can be classified under multiple subsections),

and potential measurement gaps identified to-date. The summary is followed by indicator-specific tables that include expanded information on each of the proposed indicators. Tables are divided into two parts by a double solid line:

- **Top half, indicator metadata as currently proposed:** definition, numerator, denominator, the preferred data source, alternative data sources (where applicable), proposed age range, disaggregation, indicator type[b] and use status.
- **Bottom half, additional indicator details:** initiatives including the indicator with a symbol (†) identifying the initiative upon which the indicator proposed by the GAMA AG was based, and any additional comments (where relevant).

DRAFT

[b] *Input indicators* measure human and financial resources, physical facilities, equipment, and operational policies that enable programme activities to be implemented. *Process indicators* measure the activities carried out to achieve the objectives of a programme and include both what is done and how well it is done. *Output indicators* measure the results of the processes in terms of service access, availability, quality and safety. *Outcome indicators* measure intermediate results of programmes measurable at the population level. *Impact indicators* measure long-term outcomes that programmes are designed to affect, including decreases in mortality and morbidity. In: Moller AB, Newby H, Hanson C et al. Measures matter: A scoping review of maternal and newborn indicators. PLoS One 13(10):e0204763.

Overview and summary table of proposed indicators

The GAMA AG, in collaboration with participants of the 3rd through the 6th GAMA Meetings, proposed 36 core, 1 alternative, and 15 additional indicators (Table 2).

Table 2. Distribution of core, alternative, and additional indicators by measurement domain

Measurement domain	Core	Alternative	Additional	Total
1. Social, cultural, economic, educational, and environmental determinants of adolescent health	6	1	1	8
2. Health behaviours and risks	13	0	4	17
3. Policies, programmes, and laws	2	0	2	4
4. Systems performance and interventions	3	0	2	5
5. Subjective well-being	1	0	1	2
6. Health outcomes and conditions	11	0	5	16
**Total**	**36**	**1**	**15**	**52**

For each core, alternative and additional indicator, one preferred data source was identified. One or more alternative data sources were also identified where applicable. Preferred data sources included: population-based surveys that include the general adolescent population (e.g., household surveys) or a specific subpopulation (e.g., school-based student surveys); Health Management Information System (HMIS); policy surveys (e.g., Sexual, Reproductive, Maternal, Newborn, Child, and Adolescent Health [SRMNCAH] Policy Survey, school policy survey); and civil registration and vital statistics (CRVS). Figure 1 shows the distribution of preferred data sources across all proposed indicators.

DRAFT

Figure 1. Preferred data sources for all proposed indicators by measurement domain

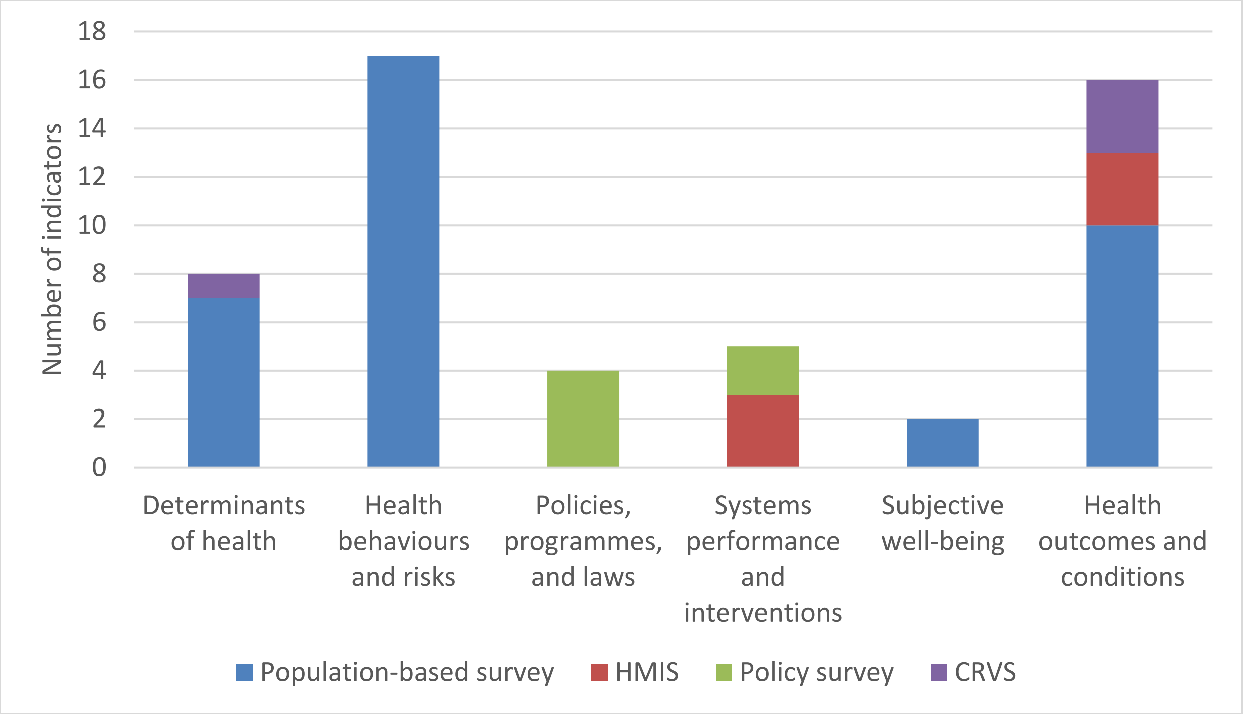

Note: HMIS = health management information systems; CRVS = civil registration and vital statistics.

DRAFT

Table 3. Summary of proposed priority indicators

No.	Indicator Name	Definition	Initiatives using this indicator
**Domain 1: Social, cultural, economic, educational, and environmental determinants of health**			
1.01	Proportion of total population that are adolescents (10–19 years), by age group (10-14, 15-19 years) and sex	Proportion of the total population in a country that are adolescents (10-19 years), by age group (10-14, 15-19 years) and sex.	EMRO core indicators for adolescent health†
1.02	Proportion of young people who have completed primary, lower secondary, and upper secondary school, by level and sex	Proportion of a cohort of young people aged 3-5 years above the intended age for the last grade of each level of education who have completed that grade.	Adolescent Country Tracker; SDGs (4.1.2)†; Lancet Commission on Adolescent Health and Wellbeing
1.03	Proportion of adolescents (10-19 years) who live below the national poverty line, by age group (10-14, 15-19 years) and sex	Proportion of adolescents (10-19 years) who live in households with income below the nationally established poverty line, by age group (10-14, 15-19 years) and sex.	INSPIRE; SDGs (1.2.1)†; UNECE Monitoring Framework (ICPD)
1.03-ALT	Proportion of adolescents (10-19 years) who live below the international poverty line, by age group (10-14, 15-19 years) and sex	Proportion of adolescents (10-19 years) who live in households with income below the international poverty line, by age group (10-14, 15-19 years) and sex.	Adolescent Country Tracker; SDGs (1.1.1)†
1.04	Proportion of adolescents (10-19 years) who live with moderate or severe food insecurity, based on the Food Insecurity Experience Scale (FIES), by age group (10-14, 15-19 years) and sex	Proportion of adolescents (10-19 years) who live in households having experienced food insecurity at moderate or severe levels, based on the FIES, during the reference period.	INSPIRE; SDGs (2.1.2)†
1.05	Proportion of female adolescents (15-19 years) who make their own informed decisions regarding sexual relations, contraceptive use, and reproductive health care	Proportion of female adolescents (15-19 years) who are married or in union and who make their own decision on all three selected areas i.e., can say no to sexual intercourse with their husband or partner if they do not want, decide on use of contraception, and decide on their own health care. Only female adolescents who satisfy all three empowerment	Global Strategy for WCAH; SDGs (5.6.1)†; UNECE Monitoring Framework (ICPD)

DRAFT

No.	Indicator Name	Definition	Initiatives using this indicator
		criteria are considered as those who make their own decisions regarding sexual and reproductive health.	
1.06	Proportion of adolescents (10–19 years) not in education, employment, or training, by age group (10-14, 15-19 years) and sex	Proportion of adolescents (10-19 years) not in education, employment, or training, by age group (10-14, 15-19 years) and sex.	Global Strategy for WCAH; Lancet Commission on Adolescent Health and Wellbeing; SDGs (8.6.1)[†]; UNECE Monitoring Framework (ICPD); Youth Development Index
A1.01	Proportion of adolescents (10-19 years) at the end of primary and at the end of lower secondary achieving at least a minimum proficiency level in (i) reading and (ii) mathematics, by age group (10-14, 15-19 years) and sex	Proportion of adolescents (10-19 years) at the end of primary education and the end of lower secondary education who achieve at least a minimum proficiency level in (a) reading and (b) mathematics.	Global Strategy for WCAH; SDGs (4.1.1 b, c)[†]
**Domain 2: Health behaviours and risks**			
2.01	Prevalence of overweight and obesity among adolescents (10-19 years), by weight status (overweight, obese), age group (10-14, 15-19 years) and sex	Proportion of adolescents (10-19 years) whose body mass index (BMI) was ≥1 standard deviation (SD) (overweight) and ≥2 SDs from the median BMI (obese) according to WHO growth reference standards for respective age and sex, by weight status (overweight, obese), age group (10-14,15-19 years) and sex.	FRESH; Global Reference List of Health Indicators for Adolescents[†]; Global Reference List of 100 Core Health Indicators; Lancet Commission on Adolescent Health and Wellbeing; UNECE Monitoring Framework (ICPD)
2.02	Prevalence of thinness among adolescents (10-19 years), by age group (10-14, 15-19 years) and sex	Proportion of adolescents (10-19 years) whose BMI was ≤ 2 SDs from the median BMI according to WHO growth reference standards for the respective age and sex, by age group (10-14, 15-19 years) and sex.	EMRO core indicators for adolescent health; Global Reference List of Health Indicators for Adolescents[†]
2.03	Past 30 day prevalence of heavy episodic drinking among adolescents (10-19 years), by age group (10-14, 15-19 years) and sex	Proportion of adolescents (10-19 years) who consumed at least six alcoholic drinks on one or more days during the past 30 days, by age group (10-14, 15-19 years) and sex.	Global Information System on Alcohol and Health[†]; INSPIRE; Lancet Commission on Adolescent Health and Wellbeing

No.	Indicator Name	Definition	Initiatives using this indicator
2.04	Past 30 day prevalence of psychoactive drug use among adolescents (10–19 years), by type of substances, age group (10-14, 15-19 years) and sex	Proportion of adolescents (10-19 years) who used any psychoactive drug other than alcohol or tobacco for non-medical purposes during the past 30 days, by type of substance, age group (10-14, 15-19 years), sex.	Adolescent Country Tracker; EMRO core indicators for adolescent health[†]; Youth Development Index
2.05	Past 30 day prevalence of tobacco use among adolescents (10–19 years), by type of tobacco used, age group (10-14, 15-19 years) and sex	Proportion of adolescents (10-19 years) who used tobacco on one or more days during the past 30 days, by type of tobacco used (cigarettes, other smoking tobacco, smokeless tobacco), age group (10-14, 15-19 years) and sex.	EMRO core indicators for adolescent health[†]; FRESH; Global Reference List of Health Indicators for Adolescents
2.06	Proportion of adolescents (10–19 years) who consumed at least 5 servings of vegetables and fruits daily during the past 7 days, by age group (10-14, 15-19 years) and sex	Proportion of adolescents (10-19 years) who consumed at least five servings of vegetables and fruits daily during the past 7 days, by age group (10-14, 15-19 years) and sex.	EMRO core indicators for adolescent health[†]
2.07	Proportion of adolescents (10–19 years) who accumulated an average of at least 60 minutes per day of moderate to vigorous-intensity physical activity during the past 7 days, by age group (10-14, 15-19 years) and sex	Proportion of adolescents (10–19 years) who accumulated an average of at least 60 minutes per day of moderate- to vigorous-intensity physical activity during the past 7 days, by age group (10-14, 15-19 years) and sex.	EMRO core indicators for adolescent health; FRESH; Global Reference List of Health Indicators for Adolescents[†]; Global Strategy for WCAH; WHO GPW 13
2.08	Proportion of adolescents (10-19 years) involved in bullying within the past 12 months, by type of involvement (victim, perpetrator, both), type of bullying (in-person, digital/cyber), age group (10-14, 15-19 years) and sex	Proportion of adolescents who were involved in bullying during the past 12 months, by type of involvement (victim, perpetrator, both), type of bullying (in-person, digital/cyber), age group (10-14, 15-19 years) and sex.	Adolescent Country Tracker; FRESH; INSPIRE; SDGs (Thematic indicator 4.a.2)[†]; UNECE Monitoring Framework (ICPD)

DRAFT

No.	Indicator Name	Definition	Initiatives using this indicator
2.09	Proportion of adolescents (15-19 years) who had their first sexual intercourse before 15 years of age, by sex	Proportion of adolescents (15-19 years) who report having had their first sexual intercourse before 15 years of age, by sex.	FRESH; Global Reference List of Health Indicators for Adolescents; INSPIRE†; Measuring the Education Sector Response to HIV and AIDS
2.10	Proportion of adolescents (10-19 years) who used a condom at last sexual intercourse, by age group (10-14, 15-19 years) and sex	Proportion of adolescents (10-19 years) who used a condom the last time they had sexual intercourse, by age group (10-14, 15-19 years) and sex.	FRESH; UNECE Monitoring Framework (ICPD)†
2.11	Proportion of live births to female adolescents (10-19 years) attended by skilled health personnel, by age group (10-14, 15-19 years)	Proportion of live births to female adolescents (10-19 years) attended by skilled health personnel, by age group (10-14, 15-19 years).	Countdown to 2030†; EMRO core indicators for adolescent health; Global Reference List of 100 Core Health Indicators; Global Strategy for WCAH; SDGs (3.1.2); UNECE Monitoring Framework (ICPD); WHO GPW 13
2.12	Proportion of adolescents (10-19 years) who used a contraceptive (modern method) at last sexual intercourse, by method used, age group (10-14, 15-19 years), and sex	Proportion of adolescents (10-19 years) who used any modern method of contraception the last time they had sexual intercourse, by method used, age group (10-14, 15-19 years) and sex.	FP2020; Countdown to 2030†; Global Reference List of 100 Core Health Indicators; UNECE Monitoring Framework (ICPD)
2.13	Proportion of adolescents (10-19 years) who have their need for contraception satisfied with modern methods, by age group (10-14, 15-19 years) and sex	Proportion of adolescents (10-19 years) who desired either to have no (additional) children or to postpone the next child and were currently using at least one modern method of contraception, by age group (10-14, 15-19 years) and sex.	Countdown to 2030; FP2020; Global Reference List of Health Indicators for Adolescents; Global Reference List of 100 Core Health Indicators; Global Strategy for WCAH; Lancet Commission on Adolescent Health and Wellbeing;

DRAFT

No.	Indicator Name	Definition	Initiatives using this indicator
			SDGs (3.7.1)[†]; UNECE Monitoring Framework (ICPD); WHO GPW 13
A2.01	Past 30 day prevalence of alcohol use among adolescents (10-19 years), by age group (10-14, 15-19 years) and sex	Proportion of adolescents (10-19 years) who consumed at least one alcoholic drink during the past 30 days, by age group (10-14, 15-19 years) and sex.	FRESH; Global Reference List of Health Indicators for Adolescents[†]
A2.02	Proportion of adolescents (10-19 years) who drank sugar-sweetened beverages one or more times per day during the past 7 days, by age group (10-14, 15-19 years) and sex	Proportion of adolescents (10-19 years) who drank sugar-sweetened beverages one or more times per day during the past 7 days, by age group (10-14, 15-19 years) and sex.	FRESH[†]
A2.03	Proportion of female adolescents (10-19 years) who were aware of menstruation before menarche, by age group (10-14, 15-19 years)	Proportion of female adolescents (10-19 years) who were aware of menstruation before menarche, by age group (10-14, 15-19 years).	None
A2.04	Past 30 day prevalence of electronic cigarette use among adolescents (10–19 years), by age group (10-14, 15-19 years) and sex	Percentage of adolescents (10-19 years) who used electronic cigarettes on one or more days during the past 30 days, by age group (10-14, 15-19 years) and sex.	Global Youth Tobacco Survey[†]
**Domain 3: Policies, programmes, and laws**			
3.01	Existence of an operational adolescent (10-19 years) health programme with coverage at the national level	The country has an adolescent (10-19 years) health programme (stand-alone or integrated) at the national level or in all subnational (first administrative level) jurisdictions with at least one designated full-time person and a regular government budget allocation to support the programme.	Countdown to 2030[†]

DRAFT

No.	Indicator Name	Definition	Initiatives using this indicator
3.02	Existence of national standards for delivery of health services to adolescents (10-19 years)	The country has national standards for delivery of health services specifically for adolescents (10-19 years) that include a clearly defined, comprehensive package of health services, the implementation of which has been monitored.	Countdown to 2030[†]
A3.01	Existence of national policy exempting adolescents (10-19 years) from user fees for specified health services in the public sector, by type of service	The existence of a national policy exempting adolescents from user fees for specified health services in the public sector (i.e., outpatient care visits, inpatient care visits, HIV testing and counselling, contraceptives, mental health, rehabilitation for substance abuse, pharmaceutical products and/or other medical supplies if required for diagnosis and treatment, testing and treatment for sexually transmitted infections and vaccination for human papilloma virus [HPV]), by type of service.	WHO SRMNCAH Policy Survey[†]
A3.02	Absence of legal age limit for married and unmarried adolescents (10-19 years) to provide consent, without spousal/parental/legal guardian consent, for specified adolescent health services, by marital status and type of service	The absence of a legal age limit to allow married and unmarried adolescents (10-19 years) to provide consent, without parental/legal guardian consent, for specified adolescent health services (i.e., contraceptive services except sterilization, emergency contraception, HIV testing and counselling services, HIV care and treatment, harm reduction interventions for injecting drug users and mental health services), by marital status and type of service.	WHO SRMNCAH Policy Survey[†]
**Domain 4: Systems performance and interventions**			
4.01	Proportion of adolescents (10-19 years) who made a visit to a health facility to receive a health service during the past 12 months, by age group (10-14, 15-19 years) and sex	Proportion of adolescents (10-19 years) who visited a health to receive a health service during the past 12 months, by age group (10-14, 15-19 years) and sex.	Global Reference List of Health Indicators for Adolescents[†]

DRAFT

No.	Indicator Name	Definition	Initiatives using this indicator
4.02	Proportion of 15-year-old adolescents covered by human papilloma virus (HPV) vaccine (last dose in schedule), by sex	Proportion of 15-year-old adolescents who received the recommended doses of HPV vaccine, by sex.	Global Strategy for WCAH; SDGs (3.b.1)†
4.03	Existence of age- and sex-disaggregated health data for adolescents (10-19 years) in the national health information system	National health information system includes data on adolescent (10-19 years) health indicators, broken down by age group (10-14, 15-19 years) and sex.	EMRO core indicators for adolescent health†
A4.01	Proportion of schools that offer comprehensive school health services	Proportion of schools that report offering comprehensive school health services, defined as school health services addressing at least four health areas relevant to their student population, including: positive health and development; unintentional injury; violence; sexual and reproductive health including HIV; communicable disease; noncommunicable disease, sensory functions, physical disability, oral health, nutrition, and physical activity; and mental health, substance use and self-harm.	None
A4.02	Proportion of schools that offer life skills-based HIV and sexuality education during the previous academic year	Proportion of schools that report offering life skills-based HIV and sexuality education (i.e., education on life skills, sexual and reproductive health, sexuality, and HIV transmission and prevention) during the previous academic year within the formal curriculum.	SDGs (Thematic Indicator 4.7.2)†; UNECE Monitoring Framework (ICPD)
**Domain 5: Subjective well-being**			
5.01	Proportion of adolescents (10-19 years) with someone to talk to when they have a worry or problem, by age group (10-14, 15-19 years) and sex	Proportion of adolescents (10-19 years) who were able to talk to someone, either most or all of the time, when they have a problem or worry having to do with difficult feelings and experiences, by age group (10-14, 15-19 years) and sex.	MMAP†

No.	Indicator Name	Definition	Initiatives using this indicator
A5.01	Proportion of adolescents (10-19 years) with a positive connection with their parent or guardian, by age group (10-14, 15-19 years) and sex	Proportion of adolescents (10-19 years) with parents/guardians who understood their problems or worries most or all of the time during the past 30 days, by age group (10-14, 15-19 years) and sex.	MMAP[†]
**Domain 6. Health outcomes and conditions**			
6.01	Adolescent (10-19 years) mortality rate, by age group (10-14, 15-19 years) and sex	Number of deaths among adolescents (10-19 years) per 100,000 adolescent population during a year, by age group (10-14, 15-19 years) and sex.	Adolescent Country Tracker; Countdown to 2030; Global Reference List of Health Indicators for Adolescents; Global Reference List of 100 Core Health Indicators; Global Strategy for WCAH[†]; Youth Development Index
6.02	Adolescent (10-19 years) mortality rate, by specified causes of death, age group (10-14, 15-19 years) and sex	Number of deaths among adolescents (10-19 years) per 100,000 adolescent population during a year, by specified causes, including priority causes of adolescent death globally (i.e., road traffic injury, interpersonal violence, drowning, self-harm, maternal conditions, diarrhoeal diseases, and HIV/AIDS) and other causes determined by the national context, by age group (10-14, 15-19 years) and sex.	Countdown to 2030[†]; Global Strategy for WCAH
6.03	Number of new cases of HIV infections among adolescents (10-19 years) per 1,000 uninfected adolescent population, by age group (10-14, 15-19 years) and sex	Number of new cases of HIV infections among adolescents (10-19 years) per 1,000 uninfected adolescent population (defined as the number of new HIV infections per 1,000 person-years among the uninfected population), by age group (10-14, 15-19 years) and sex.	Global Reference List of 100 Core Health Indicators; Global Strategy for WCAH; SDGs (3.3.1)[†]; UNECE Monitoring Framework (ICPD); WHO GPW 13
6.04	Number of new cases of sexually transmitted infections (STIs) among adolescents (10-19 years), by age group (10-14, 15-19 years) and sex	Number of new cases of STIs (syndromic or etiological reporting) other than HIV among adolescents (10-19 years) per 100,000 adolescent population during a year, by age group (10-14, 15-19 years) and sex.	Global Reference List of 100 Core Health Indicators[†]

DRAFT

No.	Indicator Name	Definition	Initiatives using this indicator
6.05	Proportion of adolescents (10-19 years) who report a suicide attempt during the past 12 months, by age group (10-14, 15-19 years) and sex	Proportion of adolescents (10-19 years) who reported a suicide attempt during the past 12 months, by age group (10-14, 15-19 years) and sex.	EMRO core indicators for adolescent health; MMAP†
6.06	Proportion of adolescents (10-19 years) who report symptoms of depression and/or anxiety, by age group (10-14, 15-19 years) and sex	Proportion of adolescents (10-19 years) with symptoms of depression and/or anxiety at a clinical threshold, by age group (10-14, 15-19 years) and sex.	EMRO core indicators for adolescent health; Global Reference List of Health Indicators for Adolescents; Global Strategy for WCAH; MMAP†
6.07	Number of new cases of specified types of injuries among adolescents (10–19 years) per 100,000 population, type of injury, age group (10-14, 15-19 years) and sex	Number of new cases of specific types of injuries (i.e., road traffic injuries, fire-related burns, poisonings, falls, and drowning) among adolescents (10-19 years) per 100,000 adolescent population during a year, by age group (10-14, 15-19 years) and sex.	EMRO core indicators for adolescent health†
6.08	Proportion of adolescents (10-19 years) who experienced physical violence during the past 12 months, by perpetrator (parents/caregivers, teachers, other adults, intimate partners, peers), age group (10-14, 15-19 years) and sex	Proportion of adolescents (10-19 years) who report having experienced physical violence (excluding sexual violence) during the past 12 months, by perpetrator (parents/caregivers, teachers, other adults, intimate partners, peers), age group (10-14, 15-19 years) and sex.	FRESH; INSPIRE†
6.09	Proportion of adolescents (10-19 years) who experienced contact sexual violence during the past 12 months, by perpetrator (parents/caregivers, teachers, other	Proportion of adolescents (10-19 years) experienced sexual violence involving physical contact (i.e., forced (completed) sex; pressured or coerced (completed) sex; attempted (but not completed) forced, coerced, or pressured sex; unwanted, non-consensual sexual	INSPIRE†

No.	Indicator Name	Definition	Initiatives using this indicator
	adults, intimate partners, peers), age group (10-14, 15-19 years) and sex	touch) during the past 12 months, by perpetrator (parents/caregivers, teachers, other adults, intimate partners, peers), age group (10-14, 15-19 years) and sex.	
6.10	Adolescent (10-19 years) birth rate, by age group (10-14, 15-19 years)	Number of live births to female adolescents (10-19 years) per 1,000 female adolescents during a year, by age group (10-14, 15-19 years).	Adolescent Country Tracker; Countdown to 2030; EMRO core indicators for adolescent health; FP2020; Global Reference List of Health Indicators for Adolescents[†]; Global Reference List of 100 Core Health Indicators; Global Strategy for WCAH; Lancet Commission on Adolescent Health and Wellbeing; SDGs (3.7.2); UNECE Monitoring Framework (ICPD); Youth Development Index
6.11	Prevalence of anaemia among adolescents (10-19 years), by age group (10-14, 15-19 years) and sex	Proportion of adolescents (10-19 years) who have a haemoglobin level below 11 g/dl for males and 12 g/dl for females, by age group (10-14, 15-19 years) and sex.	Global Strategy for WCAH; EMRO core indicators for adolescent health[†]; Lancet Commission on Adolescent Health and Wellbeing
A6.01	Proportion of adolescents (10-19 years) perpetrating physical violence during the past 12 months, by age group (10-14, 15-19 years) and sex	Proportion of adolescents (10-19 years) who perpetrated physical violence (excluding sexual violence) during the past 12 months, by age group (10-14, 15-19 years) and sex.	None
A6.02	Proportion of young women and men (18-29 years) who experienced sexual violence by age 18, by perpetrator (parents/caregivers, teachers, intimate partners, peers), age at	Proportion of young women and men (18-29 years) who report having experienced sexual violence by age 18, by perpetrator (parents/caregivers, teachers, other adults, intimate partners, peers), age at victimization (<10, 10-14, 15-18 years) and sex.	Global Reference List of 100 Core Health Indicators; Global Strategy for WCAH; INSPIRE; SDGs (16.2.3)[†]; UNECE Monitoring Framework (ICPD)

DRAFT

No.	Indicator Name	Definition	Initiatives using this indicator
	victimization (<10, 10-14, 15-18 years) and sex		
A6.03	Proportion of adolescents (10-19 years) who report suicidal thoughts during the past 2 weeks, by age group (10-14, 15-19 years) and sex	Proportion of adolescents (10-19 years) who report suicidal thoughts during the past 2 weeks, by age group (10-14, 15-19 years) and sex.	EMRO core indicators for adolescent health; MMAP†
A6.04	Proportion of adolescents (10-19 years) with symptoms of anxiety or depression who report contact with a health professional or counsellor for their mental health symptoms, by age group (10-14, 15-19 years) and sex	Proportion of adolescents (10-19 years) with symptoms of anxiety or depression who report contact with a health professional or counsellor for their mental health symptoms, by age group (10-14, 15-19 years) and sex.	Global Reference List of 100 Core Health Indicators; MMAP†
A6.05	Proportion of female adolescents (10-19 years) who have undergone female genital mutilation/cutting, by age group (10-14, 15-19 years)	Proportion of female adolescents (10-19 years) who have undergone female genital mutilation/cutting, by age group (10-14, 15-19 years).	Countdown to 2030; Global Reference List of 100 Core Health Indicators; SDGs (5.2.3)†

†Basis for indicator proposed by the GAMA AG

EMRO = WHO Regional Office for the Eastern Mediterranean; FRESH = Focusing Resources on Effective School Health; GPW 13 = 13th General Programme of Work (WHO); ICPD = International conference on population and development; MMAP = Measurement of Mental Health Among Adolescents at the Population Level; SDG = Sustainable Development Goal; UNECE = United Nations Economic Commission for Europe

DRAFT

Indicator details

1. Social, cultural, economic, educational, and environmental determinants of health

Section summary

Indicators included within section (indicator details presented below summary):

- 1.01 Proportion of total population that are adolescents (10–19 years), by age group (10-14, 15-19 years) and sex
- 1.02 Proportion of young people who have completed primary, lower secondary, and upper secondary school, by level and sex
- 1.03 Proportion of adolescents (10-19 years) who live below the national poverty line, by age group (10-14, 15-19 years) and sex
- 1.03-ALT Proportion of adolescents (10-19 years) who live below the international poverty line, by age group (10-14, 15-19 years) and sex
- 1.04 Proportion of adolescents (10-19 years) who live with moderate or severe food insecurity, based on the Food Insecurity Experience Scale (FIES), by age group (10-14, 15-19 years) and sex
- 1.05 Proportion of female adolescents (15-19 years) who make their own informed decisions regarding sexual relations, contraceptive use, and reproductive health care
- 1.06 Proportion of adolescents (10–19 years) not in education, employment, or training, by age group (10-14, 15-19 years) and sex
- A1.01 Proportion of adolescents (10-19 years) at the end of primary and at the end of lower secondary achieving at least a minimum proficiency level in (i) reading and (ii) mathematics, by age group (10-14, 15-19 years) and sex

Relevant indicators included in other sections

- None

Measurement gaps

- Gender identity
- Youth homelessness

DRAFT

1.01 Proportion of total population that are adolescents (10–19 years), by age group (10-14, 15-19 years) and sex

Definition	Proportion of the total population in a country that are adolescents (10-19 years), by age group (10-14, 15-19 years) and sex.
Numerator	Number of adolescents (10–19 years) in a country, as of 1 July of a given year (mid-year)*.
Denominator	Total population in the same country, as of 1 July in the same year*.
Preferred data source	CRVS
Alternative data source(s)	Population-based survey; Census plus modelling
Proposed age range	10-19 years
Disaggregation	Age group (10-14, 15-19 years); sex
Indicator type	Impact
Use status	In use
Initiatives using this indicator	EMRO core indicators for adolescent health†
Additional comments	*Population should be estimated as of 1 July of each year for consistency with standard demographic practices.[7]<br><br>†Basis for indicator proposed by the GAMA AG

1.02 Proportion of young people who have completed primary, lower secondary, and upper secondary school, by level and sex

Definition	Proportion of a cohort of young people aged 3-5 years above the intended age for the last grade of each level of education who have completed that grade.
Numerator	Number of young people respondents in the cohort aged 3-5 years above the intended age for the last grade level of each level of education who have completed that grade.
Denominator	Total number of young people respondents in the same cohort.
Preferred data source	Population-based survey
Alternative data source(s)	Not applicable
Proposed age range	To be determined based on national education guidelines
Disaggregation	Level of schooling; sex
Indicator type	Outcome
Use status	In use
Initiatives using this indicator	Adolescent Country Tracker; SDGs (4.1.2)[†]; Lancet Commission on Adolescent Health and Wellbeing
Additional comments	The SDG indicator metadata[8] provide the following specification on intended age for last grade:<br><br>The intended age for the last grade of each level of education is the age at which pupils would enter the grade if they had started school at the official primary entrance age, had studied full-time and had progressed without repeating or skipping a grade.<br><br>For example, if the official age of entry into primary education is 6 years, and if primary education has 6 grades, the intended age for the last grade of primary education is 11 years. In this case, 14-16 years (11 + 3 = 14 and 11 + 5 = 16) would be the reference age group for calculation of the primary completion rate.<br><br>[†]Basis for indicator proposed by the GAMA AG

1.03 Proportion of adolescents (10-19 years) who live below the national poverty line, by age group (10-14, 15-19 years) and sex

Definition	Proportion of adolescents (10-19 years) who live in households with income below the nationally established poverty line, by age group (10-14, 15-19 years) and sex.
Numerator	Number of adolescent respondents (10-19 years) who live in households with income below the nationally established poverty line.
Denominator	Total number of adolescent respondents (10-19 years).
Preferred data source	Population-based survey
Alternative data source(s)	Not applicable
Proposed age range	10-19 years
Disaggregation	Age group (10-14, 15-19 years); sex
Indicator type	Impact
Use status	In use with modifications
Initiatives using this indicator	INSPIRE; SDGs (1.2.1)[†]; UNECE Monitoring Framework (ICPD)
Additional comments	Computation of this indicator requires the existence of a nationally defined poverty line. In cases where the multiple national poverty lines have been established, the most recently established poverty line should be used.<br><br>Computation details for the national/international poverty rate are available in the SDG indicator metadata.[8]<br><br>These approaches generally depend on household survey data to determine the proportion of the population living below the respective poverty line. Further computation will be necessary to determine the proportion of adolescents living in households below the respective poverty line.<br><br>[†]Basis for indicator proposed by the GAMA AG

1.03-ALT Proportion of adolescents (10-19 years) who live below the international poverty line, by age group (10-14, 15-19 years) and sex

Definition	Proportion of adolescents (10-19 years) who live in households with income below the international poverty line*, by age group (10-14, 15-19 years) and sex.
Numerator	Number of adolescent respondents (10-19 years) who live in households within income below the international poverty line*.
Denominator	Total number of adolescent respondents (10-19 years).
Preferred data source	Population-based survey
Alternative data source(s)	Not applicable
Proposed age range	10-19 years
Disaggregation	Age group (10-14, 15-19 years); sex
Indicator type	Impact
Use status	In use with modifications
Initiatives using this indicator	Adolescent Country Tracker; SDGs (1.1.1)†
Additional comments	*The international poverty line is currently set at $1.90 a day at 2011 international prices.<br><br>Computation details for the national/international poverty rate are available in SDG indicator metadata.[8]<br><br>These approaches generally depend on household survey data to determine the proportion of the population living below the respective poverty line. Further computation will be necessary to determine the proportion of adolescents living in households below the respective poverty line.<br><br>†Basis for indicator proposed by the GAMA AG

DRAFT

1.04 Proportion of adolescents (10-19 years) who live with moderate or severe food insecurity, based on the Food Insecurity Experience Scale (FIES), by age group (10-14, 15-19 years) and sex

Definition	Proportion of adolescents (10-19 years) who live in households having experienced food insecurity at moderate or severe levels, based on the FIES*, during the reference period.
Numerator	Number of adolescent respondents (10-19 years) who live in households that have experienced food insecurity at moderate or severe levels, based on the FIES*, during the reference period.
Denominator	Total number of adolescent respondents (10-19 years).
Preferred data source	Population-based survey
Alternative data source(s)	Not applicable
Proposed age range	10-19 years
Disaggregation	Age group (10-14, 15-19 years); sex
Indicator type	Impact
Use status	In use with modifications
Initiatives using this indicator	INSPIRE; SDGs (2.1.2)[†]
Additional comments	* The severity of food insecurity, defined as a latent trait, is measured on the FIES global reference scale, a measurement standard established by the Food and Agriculture Organization through the application of the Food Insecurity Experience Scale in more than 140 countries worldwide, starting in 2014.[8]<br><br>The FIES considers the three classes of (a) food security or mild food insecurity; b) moderate or severe food insecurity, and (c) severe food insecurity.[9,10] Detailed computational information is available in the SDG metadata.[8]<br><br>This approach can be used to classify households by food insecurity status. Additional computation would be required to determine the proportion of adolescents in the survey living in households with moderate or severe food insecurity.<br><br>[†]Basis for indicator proposed by the GAMA AG

DRAFT

1.05 Proportion of female adolescents (15-19 years) who make their own informed decisions regarding sexual relations, contraceptive use, and reproductive health care

Definition	Proportion of female adolescents (15-19 years) who are married or in union and who make their own decision on all three selected areas i.e., can say no to sexual intercourse* with their husband or partner if they do not want, decide on use of contraception, and decide on their own health care. Only female adolescents who satisfy all three empowerment criteria are considered as those who make their own decisions regarding sexual and reproductive health.
Numerator	Number of married or in union female adolescents respondents (15-19 years):<br>● who can say “no” to sex*; and<br>● for whom the decision on contraception is not mainly made by the husband/partner; and<br>● for whom decision on health care for themselves is not usually made by the husband/partner or someone else<br><br>Only female adolescents who satisfy all three empowerment criteria are included in the numerator.
Denominator	Total number female adolescent respondents (15-19 years) who are married or in union.
Preferred data source	Population-based survey
Alternative data source(s)	Not applicable
Proposed age range	15-19 years
Disaggregation	None
Indicator type	Outcome
Use status	In use
Initiatives using this indicator	Global Strategy for WCAH; SDGs (5.6.1)[†]; UNECE Monitoring Framework (ICPD)
Additional comments	This composite indicator assesses the proportion of female adolescents (15-19 years) for whom all three specified criteria are met. Where relevant, countries may choose to also report on the proportion of female adolescents satisfying one or more of the empowerment criteria.<br><br>*Sexual intercourse includes any sexual contact involving the genital area, including oral sex, vaginal sex, and anal sex.<br><br>[†]Basis for indicator proposed by the GAMA AG

DRAFT

1.06 Proportion of adolescents (10–19 years) not in education, employment, or training, by age group (10-14, 15-19 years) and sex

Definition	Proportion of adolescents (10-19 years) not in education, employment or training*, by age group (10-14, 15-19 years) and sex.
Numerator	Number of adolescent respondents (10-19 years) not in education, employment, or training*.
Denominator	Total number of adolescent respondents (10-19 years).
Preferred data source	Population-based survey
Alternative data source(s)	Not applicable
Proposed age range	10-19 years
Disaggregation	Age group (10-14, 15-19 years); sex
Indicator type	Outcome
Use status	In use with modifications
Initiatives using this indicator	Global Strategy for WCAH; Lancet Commission on Adolescent Health and Wellbeing; SDGs (8.6.1)[†]; UNECE Monitoring Framework (ICPD); Youth Development Index
Additional comments	*The SDG indicator metadata[8] provide the following definition of employment, education, and training:<br><br>According to the International Standard Classification of Education (ISCED), education is defined as organized and sustained communication designed to bring about learning.[11] Formal education is defined in ISCED as education that is institutionalized, intentional, and planned through public organizations and recognized private bodies and, in their totality, make up the formal education system of a country.<br><br>Non-formal education, like formal education is defined in ISCED as education that is institutionalized, intentional and planned by an education provider but is considered an addition, alternative and/or a complement to formal education. It may be short in duration and/or low in intensity and it is typically provided in the form of short courses, workshops or seminars. Informal learning is defined in ISCED as forms of learning that are intentional or deliberate, but not institutionalized. It is thus less organized and less structured than either formal or non-formal education. Informal learning may include learning activities that occur in the family, in the work place, in the local community, and in daily

	life, on a self-directed, family-directed or socially-directed basis. For the purposes of this indicator, persons will be considered in education if they are in formal or non-formal education, as described above, but excluding informal learning.<br><br>Employment is defined as all persons of working age who, during a short reference period (one week), were engaged in any activity to produce goods or provide services for pay or profit.<br><br>For the purpose of this indicator, persons are considered to be in training if they are in a non-academic learning activity through which they acquire specific skills intended for vocational or technical jobs. Vocational training prepares trainees for jobs that are based on manual or practical activities, and for skilled operative jobs, both blue and white collar related to a specific trade, occupation or vocation. Technical training on the other hand imparts learning that can be applied in intermediate-level jobs, in particular those of technicians and middle managers.<br><br>[†]Basis for indicator proposed by the GAMA AG

DRAFT

A1.01 Proportion of adolescents (10-19 years) at the end of primary and at the end of lower secondary achieving at least a minimum proficiency level in (i) reading and (ii) mathematics, by age group (10-14, 15-19 years) and sex

Definition	Proportion of adolescents (10-19 years) at the end of primary education and the end of lower secondary education who achieve at least a minimum proficiency level* in (a) reading and (b) mathematics.
Numerator	Number of adolescent respondents (10-19 years) at the end of primary education and the end of lower secondary education achieving at least a minimum proficiency level* in (a) reading and (b) mathematics.
Denominator	Total number of adolescent respondents at the end of primary education and the end of lower secondary education.
Preferred data source	Population-based survey
Alternative data source(s)	Not applicable
Proposed age range	10-19 years
Disaggregation	Education level (end of primary, end of lower secondary); subject (reading, mathematics); sex
Indicator type	Impact
Use status	In use
Initiatives using this indicator	Global Strategy for WCAH; SDGs (4.1.1 b, c)[†]
Additional comments	*The minimum proficiency level will be measured relative to new common reading and mathematics scales currently in development.<br><br>The SDG indicator metadata provide additional details of minimum proficiency levels and indicator computation.[8]<br><br>[†]Basis for indicator proposed by the GAMA AG

DRAFT

2. Health behaviours and risks

Section summary

Indicators included within section (indicator details presented below summary):

- 2.01 Prevalence of overweight and obesity among adolescents (10-19 years), by weight status (overweight, obese), age group (10-14, 15-19 years) and sex
- 2.02 Prevalence of thinness among adolescents (10-19 years), by age group (10-14, 15-19 years) and sex
- 2.03 Past 30 day prevalence of heavy episodic drinking among adolescents (10-19 years), by age group (10-14, 15-19 years) and sex
- 2.04 Past 30 day prevalence of psychoactive drug use among adolescents (10–19 years), by type of substances, age group (10-14, 15-19 years) and sex
- 2.05 Past 30 day prevalence of tobacco use among adolescents (10–19 years), by type of tobacco used, age group (10-14, 15-19 years) and sex
- 2.06 Proportion of adolescents (10–19 years) who consumed at least 5 servings of vegetables and fruits daily during the past 7 days, by age group (10-14, 15-19 years) and sex
- 2.07 Proportion of adolescents (10–19 years) who accumulated an average of at least 60 minutes per day of moderate to vigorous-intensity physical activity during the past 7 days, by age group (10-14, 15-19 years) and sex
- 2.08 Proportion of adolescents (10-19 years) involved in bullying within the past 12 months, by type of involvement (victim, perpetrator, both), type of bullying (in-person, digital/cyber), age group (10-14, 15-19 years) and sex
- 2.09 Proportion of adolescents (15-19 years) who had their first sexual intercourse before 15 years of age, by sex
- 2.10 Proportion of adolescents (10-19 years) who used a condom at last sexual intercourse, by age group (10-14, 15-19 years) and sex
- 2.11 Proportion of live births to female adolescents (10-19 years) attended by skilled health personnel, by age group (10-14, 15-19 years)
- 2.12 Proportion of adolescents (10-19 years) who used a contraceptive (modern method) at last sexual intercourse, by method use, age group (10-14, 15-19 years), and sex
- 2.13 Proportion of adolescents (10-19 years) who have their need for contraception satisfied with modern methods, by age group (10-14, 15-19 years) and sex
- A2.01 Past 30 day prevalence of alcohol use among adolescents (10-19 years), by age group (10-14, 15-19 years) and sex

DRAFT

- A2.02 Proportion of adolescents (10-19 years) who drank sugar-sweetened beverages one or more times per day during the past 7 days, by age group (10-14, 15-19 years) and sex
- A2.03 Proportion of female adolescents (10-19 years) who were aware of menstruation before menarche, by age group (10-14, 15-19 years)
- A2.04 Past 30 day prevalence of electronic cigarette use among adolescents (10–19 years), by age group (10-14, 15-19 years) and sex

Relevant indicators included in other sections

- 1.04 Proportion of adolescents (10-19 years) who live with moderate or severe food insecurity, based on the Food Insecurity Experience Scale (FIES), by age group (10-14, 15-19 years) and sex
- 4.02 Proportion of 15-year-old adolescents covered by human papilloma virus (HPV) vaccine (last dose in schedule), by sex
- A4.01 Proportion of schools that offer comprehensive school health services
- A4.02 Proportion of schools that offer life skills-based HIV and sexuality education during the previous academic year
- 6.08 Proportion of adolescents (10-19 years) who experienced physical violence during the past 12 months, by perpetrator (parents/caregivers, teachers, other adults, intimate partners, peers), age group (10-14, 15-19 years) and sex
- 6.09 Proportion of adolescents (10-19 years) who experienced contact sexual violence during the past 12 months, by perpetrator (parents/caregivers, teachers, other adults, intimate partners, peers), age group (10-14, 15-19 years) and sex
- 6.11 Prevalence of anaemia among adolescents (10-19 years), by age group (10-14, 15-19 years) and sex
- A6.01 Proportion of adolescents (10-19 years) perpetrating physical violence during the past 12 months, by age group (10-14, 15-19 years) and sex
- A6.02 Proportion of young women and men (18-29 years) who experienced sexual violence by age 18, by perpetrator, age at victimization (<10, 10-14, 15-18 years) and sex
- A6.05 Proportion of female adolescents (10-19 years) who have undergone female genital mutilation/cutting, by age group (10-14, 15-19 years)

Measurement gaps

- Active travel
- Addictive behaviours, particularly gaming disorder
- Comprehensive abortion care

DRAFT

- Counselling bias for contraception methods (e.g., adolescents not counselled on all methods, such as emergency contraception)
- Online behaviours
- Pre-coital sexual activity
- Sexual and reproductive health indicators for younger adolescents (body pride, comfort with one’s sexuality, puberty, menstruation/menstrual health)
- Sports participation

DRAFT

2.01 Prevalence of overweight and obesity among adolescents (10-19 years), by weight status (overweight, obese), age group (10-14, 15-19 years) and sex

Definition	Proportion of adolescents (10-19 years) whose body mass index (BMI) was ≥1 standard deviation (SD) (overweight) and ≥2 SDs from the median BMI (obese) according to WHO growth reference standards for respective age and sex, by weight status (overweight, obese), age group (10-14,15-19 years) and sex.
Numerator	Number of adolescent respondents (10-19 years) whose BMI was ≥1 SD (overweight) and ≥2 SDs from the median BMI (obese) according to WHO growth reference standards for respective age and sex.
Denominator	Total number of adolescent respondents (10-19 years).
Preferred data source	Population-based survey
Alternative data source(s)	Not applicable
Proposed age range	10-19 years
Disaggregation	Weight status (overweight, obese); age group (10-14, 15-19 years); sex
Indicator type	Outcome
Use status	In use
Initiatives using this indicator	FRESH; Global Reference List of Health Indicators for Adolescents[†]; Global Reference List of 100 Core Health Indicators; Lancet Commission on Adolescent Health and Wellbeing; UNECE Monitoring Framework (ICPD)
Additional comments	Objectively measured BMI is preferable to self-report.<br><br>[†]Basis for indicator proposed by the GAMA AG

2.02 Prevalence of thinness among adolescents (10-19 years), by age group (10-14, 15-19 years) and sex

Definition	Proportion of adolescents (10-19 years) whose BMI was ≤ 2 SDs from the median BMI according to WHO growth reference standards for the respective age and sex, by age group (10-14, 15-19 years) and sex.
Numerator	Number of adolescent respondents (10-19 years) whose BMI was ≤ 2 SDs from the median BMI according to WHO growth reference standards for the respective age and sex.
Denominator	Total number of adolescent respondents (10-19 years).
Preferred data source	Population-based survey
Alternative data source(s)	Not applicable
Proposed age range	10-19 years
Disaggregation	Age group (10-14, 15-19 years); sex
Indicator type	Outcome
Use status	In use
Initiatives using this indicator	EMRO core indicators for adolescent health; Global Reference List of Health Indicators for Adolescents†
Additional comments	Objectively measured BMI is preferable to self-report.<br><br>Within this age group, thinness is the relevant measurement for ≤2 SD from the median BMI. Alternative cut-offs of mild underweight (1-2 SD from median BMI) and severe thinness (≤3 SD from median BMI) may also be considered if relevant.<br><br>†Basis for indicator proposed by the GAMA AG

2.03 Past 30 day prevalence of heavy episodic drinking among adolescents (10-19 years), by age group (10-14, 15-19 years) and sex

Definition	Proportion of adolescents (10-19 years) who consumed at least six alcoholic drinks* on one or more days during the past 30 days, by age group (10-14, 15-19 years) and sex.
Numerator	Number of adolescent respondents (10-19 years) who reported consuming at least six alcoholic drinks* on one or more days during the past 30 days.
Denominator	Total number of adolescent respondents (10-19 years).
Preferred data source	Population-based survey
Alternative data source(s)	Not applicable
Proposed age range	10-19 years
Disaggregation	Age group (10-14, 15-19 years); sex
Indicator type	Outcome
Use status	In use with modifications
Initiatives using this indicator	Global Information System on Alcohol and Health†; INSPIRE; Lancet Commission on Adolescent Health and Wellbeing
Additional comments	This indicator uses the same threshold of six alcoholic drinks for all adolescents, regardless of sex, age, or other characteristic. It is recommended that data collection for this indicator obtain the exact number of drinks consumed so that alternative thresholds may be considered where relevant.<br><br>*A standard alcoholic drink corresponds to 10 grams of alcohol. The exact amount of alcohol in an alcoholic drink may be calculated by first multiplying the volume (ml) of the drink by the percentage of pure alcohol. This calculation provides the volume (ml) of alcohol in the drink. The volume should then be multiplied by a conversion factor of 0.79 (g/ml) to determine the amount of alcohol in the drink. For example, 1 can beer (330 ml) at 5% (strength) x 0.79 (conversion factor) = 13 grams of alcohol.[12]<br><br>Drinking alcohol does not include drinking a few sips of wine for religious purposes.

<table>
<tr>
<td></td>
<td>1 standard drink =

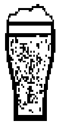 1 standard bottle of regular beer (285ml)
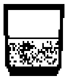 1 single measure of spirits (30ml)
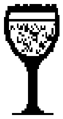 1 medium size glass of wine (120ml)
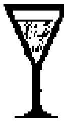 1 measure of aperitif (60ml)

†Basis for indicator proposed by the GAMA AG</td>
</tr>
</table>

DRAFT

2.04 Past 30 day prevalence of psychoactive drug use among adolescents (10–19 years), by type of substances, age group (10-14, 15-19 years) and sex

Definition	Proportion of adolescents (10-19 years) who used any psychoactive drug* other than alcohol or tobacco for non-medical purposes during the past 30 days, by type of substance, age group (10-14, 15-19 years), sex.
Numerator	Number of adolescent respondents (10-19 years) who reported using any psychoactive drug* other than alcohol or tobacco for non-medical purposes during the past 30 days.
Denominator	Total population of adolescent respondents (10-19 years).
Preferred data source	Population-based survey
Alternative data source(s)	Not applicable
Proposed age range	10-19 years
Disaggregation	Type of substance (e.g., cannabis, opiates); age group (10-14, 15-19 years); sex
Indicator type	Outcome
Use status	In use with modifications
Initiatives using this indicator	Adolescent Country Tracker; EMRO core indicators for adolescent health[†]; Youth Development Index
Additional comments	*Psychoactive drugs and substances are defined as “a substance that, when ingested, affects mental processes, e.g., cognition or affect. This term and its equivalent, psychotropic drug, are the most neutral and descriptive terms for the whole class of substances, licit and illicit, of interest to drug policy.”[13]<br><br>[†]Basis for indicator proposed by the GAMA AG

2.05 Past 30 day prevalence of tobacco use among adolescents (10–19 years), by type of tobacco used, age group (10-14, 15-19 years) and sex

Definition	Proportion of adolescents (10-19 years) who used tobacco on one or more days during the past 30 days, by type of tobacco used (cigarettes, other smoking tobacco, smokeless tobacco), age group (10-14, 15-19 years) and sex.
Numerator	Number of adolescent respondents (10-19 years) who reported using tobacco on one or more days during the past 30 days.
Denominator	Total number of adolescent respondents (10-19 years).
Preferred data source	Population-based survey
Alternative data source(s)	Not applicable
Proposed age range	10-19 years
Disaggregation	Type of tobacco used (i.e., cigarettes, other smoking tobacco, smokeless tobacco); age group (10-14, 15-19 years); sex
Indicator type	Outcome
Use status	In use
Initiatives using this indicator	EMRO core indicators for adolescent health[†]; FRESH; Global Reference List of Health Indicators for Adolescents
Additional comments	Tobacco use includes use of cigarettes, other smoked tobacco products, and smokeless tobacco products and includes both daily and non-daily use.[14] Current tobacco use does not include current use of electronic cigarettes, which is assessed separately (see indicator A2.04).<br><br>[†]Basis for indicator proposed by the GAMA AG

2.06 Proportion of adolescents (10–19 years) who consumed at least 5 servings of vegetables and fruits daily during the past 7 days, by age group (10-14, 15-19 years) and sex

<table>
<tr><td>Definition</td><td>Proportion of adolescents (10-19 years) who consumed at least five servings* of vegetables and fruits daily during the past 7 days, by age group (10-14, 15-19 years) and sex.</td></tr>
<tr><td>Numerator</td><td>Number of adolescent respondents (10-19 years) who reported consuming at least five servings* of vegetables and fruits daily during the past 7 days.</td></tr>
<tr><td>Denominator</td><td>Total number of adolescent respondents (10-19 years).</td></tr>
<tr><td>Preferred data source</td><td>Population-based survey</td></tr>
<tr><td>Alternative data source(s)</td><td>Not applicable</td></tr>
<tr><td>Proposed age range</td><td>10-19 years</td></tr>
<tr><td>Disaggregation</td><td>Age group (10-14, 15-19 years); sex</td></tr>
<tr><td>Indicator type</td><td>Outcome</td></tr>
<tr><td>Use status</td><td>In use</td></tr>
<tr><td>Initiatives using this indicator</td><td>EMRO core indicators for adolescent health[†]</td></tr>
<tr><td>Additional comments</td><td>*One standard serving = 80 grams (translated into different units of cup depending on type of vegetable and standard cup measures in the country).
<table>
<tr><th></th><th>Considered to be:</th><th>1 Serving =</th><th>Examples</th></tr>
<tr><td rowspan="2">Vegetables</td><td>Raw, green leafy vegetables</td><td>1 cup</td><td>Spinach, salad, etc.</td></tr>
<tr><td>Other vegetables, cooked or chopped raw</td><td>½ cup</td><td>Tomatoes, carrots, pumpkin, corn, Chinese cabbage, fresh beans, onion, etc.
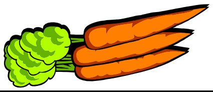 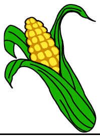</td></tr>
<tr><td rowspan="2">Fruit</td><td>Apple, banana, orange</td><td>1 medium size piece</td><td>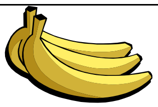 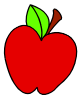</td></tr>
<tr><td>Chopped or cooked fruit</td><td>½ cup</td><td></td></tr>
</table>
Note: tubers such as potato and cassava should not be included.

[†]Basis for indicator proposed by the GAMA AG</td></tr>
</table>

DRAFT

2.07 Proportion of adolescents (10–19 years) who accumulated an average of at least 60 minutes per day of moderate to vigorous-intensity physical activity during the past 7 days, by age group (10-14, 15-19 years) and sex

Definition	Proportion of adolescents (10–19 years) who accumulated an average of at least 60 minutes per day of moderate- to vigorous-intensity physical activity* during the past 7 days, by age group (10-14, 15-19 years) and sex.
Numerator	Number of adolescent respondents (10-19 years) who accumulated an average of at least 60 minutes per day of moderate to vigorous-intensity physical activity* during the past 7 days.
Denominator	Total number of adolescent respondents (10-19 years).
Preferred data source	Population-based survey
Alternative data source(s)	Not applicable
Proposed age range	10-19 years
Disaggregation	Age group (10-14, 15-19 years); sex
Indicator type	Outcome
Use status	In use with modifications
Initiatives using this indicator	EMRO core indicators for adolescent health; FRESH; Global Reference List of Health Indicators for Adolescents†; Global Strategy for WCAH; WHO GPW 13
Additional comments	Device-based measurement of physical activity (e.g., via accelerometer/movement sensor) is preferable to self-reported data.<br><br>*For a definition of moderate and vigorous-intensity physical activity, including example activities, please see the WHO guidelines on physical activity and sedentary behaviour.[15]<br><br>†Basis for indicator proposed by the GAMA AG

DRAFT

2.08 Proportion of adolescents (10-19 years) involved in bullying within the past 12 months, by type of involvement (victim, perpetrator, both), type of bullying (in-person, digital/cyber), age group (10-14, 15-19 years) and sex

Definition	Proportion of adolescents who were involved in bullying* during the past 12 months, by type of involvement (victim, perpetrator, both), type of bullying (in-person, digital/cyber), age group (10-14, 15-19 years) and sex.
Numerator	Number of adolescent respondents (10-19 years) who reported bullying and/or being bullied* during the past 12 months.
Denominator	Total number of adolescent respondents (10-19 years).
Preferred data source	Population-based survey.
Alternative data source(s)	Not applicable
Proposed age range	10-19 years
Disaggregation	Type of involvement (victim, perpetrator, both); type of bullying (e.g., in-person vs. digital/cyber); age group (10-14, 15-19 years); sex
Indicator type	Outcome
Use status	In use with modifications
Initiatives using this indicator	Adolescent Country Tracker; FRESH; INSPIRE; SDGs (Thematic indicator 4.a.2)[†]; UNECE Monitoring Framework (ICPD)
Additional comments	*The INSPIRE indicator guidance[16] provides the following definitions for bullying, including in-person and cyber (digital) bullying:<br><br>**Bullying:** Unwanted, aggressive behaviour by another child or a group of children who are neither siblings nor in a romantic relationship with the victim. Bullying involves a repeated pattern of physical, psychological or social aggression likely to cause harm, and often takes place in schools and other settings where children gather, as well as online. It may occur in person or online (cyber bullying).<br>• **In-person bullying** may include: physical acts, such as pushing and hitting, and verbal acts, such as making fun of people for their race, religion or appearance, or sexual comments or jokes. Bullying may also include repeatedly leaving people out or ignoring them.<br>• **Cyber (digital) bullying** may include: sending hurtful messages or posting them online where others can see; threatening someone online; creating a website that makes fun of someone; and sharing or posting hurtful images or

	pictures without permission through texting, emails, social media or other online channels.<br><br>†Basis for indicator proposed by the GAMA AG

DRAFT

2.09 Proportion of adolescents (15-19 years) who had their first sexual intercourse before 15 years of age, by sex

Definition	Proportion of adolescents (15-19 years) who had their first sexual intercourse* before 15 years of age, by sex.
Numerator	Number of adolescent respondents (15-19 years) who reported having first sexual intercourse* before 15 years of age.
Denominator	Total number of adolescent respondents (15-19 years).
Preferred data source	Population-based survey
Alternative data source(s)	Not applicable
Proposed age range	<15 years
Disaggregation	Sex
Indicator type	Outcome
Use status	In use
Initiatives using this indicator	FRESH; Global Reference List of Health Indicators for Adolescents; INSPIRE[†]; Measuring the Education Sector Response to HIV and AIDS
Additional comments	*Sexual intercourse includes any sexual contact involving the genital area, including oral sex, vaginal sex, and anal sex.<br><br>[†]Basis for indicator proposed by the GAMA AG

DRAFT

2.10 Proportion of adolescents (10-19 years) who used a condom at last sexual intercourse, by age group (10-14, 15-19 years) and sex

Definition	Proportion of adolescents (10-19 years) who used a condom the last time they had sexual intercourse*, by age group (10-14, 15-19 years) and sex.
Numerator	Number of adolescent respondents (10-19 years) who reported using a condom the last time they had sexual intercourse*.
Denominator	Total number of adolescent respondents (10-19 years) who reported having had sexual intercourse.
Preferred data source	Population-based survey
Alternative data source(s)	Not applicable
Proposed age range	10-19 years
Disaggregation	Age group (10-14, 15-19 years); sex
Indicator type	Outcome
Use status	In use with modifications
Initiatives using this indicator	FRESH; UNECE Monitoring Framework (ICPD)[†]
Additional comments	*Sexual intercourse includes any sexual contact involving the genital area, including oral sex, vaginal sex, and anal sex.<br><br>[†]Basis for indicator proposed by the GAMA AG

2.11 Proportion of live births to female adolescents (10-19 years) attended by skilled health personnel, by age group (10-14, 15-19 years)

Definition	Proportion of live births to female adolescents (10-19 years) attended by skilled health personnel*, by age group (10-14, 15-19 years).
Numerator	Number of female adolescent respondents (10-19 years) who reported being attended by skilled health personnel* at the time of childbirth.
Denominator	Total number of female adolescent respondents (10-19 years) who reported a live birth.
Preferred data source	Population-based survey
Alternative data source(s)	Not applicable
Proposed age range	10-19 years
Disaggregation	Age group (10-14, 15-19 years)
Indicator type	Outcome
Use status	In use with modifications
Initiatives using this indicator	Countdown to 2030†; EMRO core indicators for adolescent health; Global Reference List of 100 Core Health Indicators; Global Strategy for WCAH; SDGs (3.1.2); UNECE Monitoring Framework (ICPD); WHO GPW 13
Additional comments	*See the 2018 joint statement by WHO, UNFPA, UNICEF, the International Confederation of Midwives, the International Council of Nurses, the International Federation of Gynaecology and Obstetrics and the International Paediatric Association for the 2018 definition of skilled health personnel providing care during childbirth.[17]<br><br>†Basis for indicator proposed by the GAMA AG

2.12 Proportion of adolescents (10-19 years) who used a contraceptive (modern method) at last sexual intercourse, by method used, age group (10-14, 15-19 years), and sex

Definition	Proportion of adolescents (10-19 years) who used any modern method of contraception* the last time they had sexual intercourse**, by method used, age group (10-14, 15-19 years) and sex.
Numerator	Number of adolescent respondents (10-19 years) who reported using a modern method of contraception* at last sexual intercourse**.
Denominator	Total number of adolescent respondents (10-19 years) who reported having had sexual intercourse**.
Preferred data source	Population-based survey
Alternative data source(s)	Not applicable
Proposed age range	10-19 years
Disaggregation	Method used; age group (10-14, 15-19 years); sex
Indicator type	Outcome
Use status	In use with modifications
Initiatives using this indicator	FP2020; Countdown to 2030†; Global Reference List of 100 Core Health Indicators; UNECE Monitoring Framework (ICPD)
Additional comments	This indicator is to be assessed among both married and unmarried adolescents. Where relevant, disaggregation by marital status may be considered.<br><br>*Modern methods include oral contraceptive pill, intra-uterine device, injectables, implants, male condom, lactational amenorrhea method, standard days method, female condom, emergency contraception, diaphragm, and foam or jelly.[18,19]<br><br>The methods listed in the definition above reflect the set of modern methods under consideration when developing additional metadata for this indicator and will need to be revisited as new evidence emerges.<br><br>Two additional methods are generally included among the general population, female sterilization and male sterilization, though these were omitted as they were not considered relevant for adolescents.<br><br>** Sexual intercourse includes any sexual contact involving the genital area, including oral sex, vaginal sex, and anal sex.<br><br>†Basis for indicator proposed by the GAMA AG

2.13 Proportion of adolescents (10-19 years) who have their need for contraception satisfied with modern methods, by age group (10-14, 15-19 years) and sex

Definition	Proportion of adolescents (10-19 years) who desired either to have no (additional) children or to postpone the next child and were currently using at least one modern method of contraception*, by age group (10-14, 15-19 years) and sex.
Numerator	Number of adolescent respondents (10-19 years old) who reported currently using, or whose sexual partner was currently using, at least one modern contraceptive method*.
Denominator	Total number of adolescent respondents (10-19 years) with demand for family planning (the sum of contraceptive prevalence (any method) and the unmet need for family planning).
Preferred data source	Population-based survey
Alternative data source(s)	Not applicable
Proposed age range	10-19 years
Disaggregation	Age group (10-14, 15-19 years); sex
Indicator type	Outcome
Use status	In use with modifications
Initiatives using this indicator	Countdown to 2030; FP2020; Global Reference List of Health Indicators for Adolescents; Global Reference List of 100 Core Health Indicators; Global Strategy for WCAH; Lancet Commission on Adolescent Health and Wellbeing; SDGs (3.7.1)[†]; UNECE Monitoring Framework (ICPD); WHO GPW 13
Additional comments	This indicator is also referred to as the demand for family planning satisfied with modern methods.<br><br>Where relevant, additional disaggregation by marital status may be considered.<br><br>*Modern methods include oral contraceptive pill, intra-uterine device, injectables, implants, male condom, lactational amenorrhea method, standard days method, female condom, emergency contraception, diaphragm, and foam or jelly.[18,19]<br><br>Note that the methods listed in the definition above reflect the set of modern methods under consideration when developing metadata for this indicator and will need to be revisited as new evidence emerges.

	Two additional methods are generally included among the general population, female sterilization and male sterilization, though these were omitted as they were not considered relevant for adolescents.<br><br>†Basis for indicator proposed by the GAMA AG

DRAFT

A2.01 Past 30 day prevalence of alcohol use among adolescents (10-19 years), by age group (10-14, 15-19 years) and sex

Definition	Proportion of adolescents (10-19 years) who consumed at least one alcoholic drink* during the past 30 days, by age group (10-14, 15-19 years) and sex.
Numerator	Number of adolescent respondents (10-19 years) who reported consuming at least one alcoholic drink* during the past 30 days.
Denominator	Total number of adolescent respondents (10-19 years).
Preferred data source	Population-based survey
Alternative data source(s)	Not applicable
Proposed age range	10-19 years
Disaggregation	Age group (10-14, 15-19 years); sex
Indicator type	Outcome
Use status	In use
Initiatives using this indicator	FRESH; Global Reference List of Health Indicators for Adolescents†
Additional comments	*A standard alcoholic drink corresponds to 10 grams of alcohol. The exact amount of alcohol in an alcoholic drink may be calculated by first multiplying the volume (ml) of the drink by the percentage of pure alcohol. This calculation provides the volume (ml) of alcohol in the drink. The volume should then be multiplied by a conversion factor of 0.79 (g/ml) to determine the amount of alcohol in the drink. For example, 1 can beer (330 ml) at 5% (strength) x 0.79 (conversion factor) = 13 grams of alcohol.[12]<br><br>Drinking alcohol does not include drinking a few sips of wine for religious purposes.<br><br>1 standard drink =<br>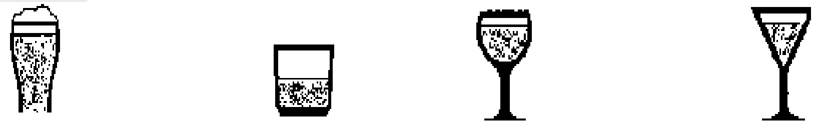<br>1 standard bottle of **regular beer** (285ml) — 1 single measure of **spirits** (30ml) — 1 medium size glass of **wine** (120ml) — 1 measure of **aperitif** (60ml)<br><br>†Basis for indicator proposed by the GAMA AG

DRAFT

A2.02 Proportion of adolescents (10-19 years) who drank sugar-sweetened beverages one or more times per day during the past 7 days, by age group (10-14, 15-19 years) and sex

Definition	Proportion of adolescents (10-19 years) who drank sugar-sweetened beverages* one or more times per day during the past 7 days, by age group (10-14, 15-19 years) and sex.
Numerator	Number of adolescent respondents (10-19 years) who reported drinking sugar-sweetened beverages* one or more times per day during the past 7 days.
Denominator	Total number of adolescent respondents (10-19 years).
Preferred data source	Population-based survey
Alternative data source(s)	Not applicable
Proposed age range	10-19 years
Disaggregation	Age group (10-14, 15-19 years), sex
Indicator type	Outcome
Use status	In use with modifications
Initiatives using this indicator	FRESH[†]
Additional comments	*Sugar-sweetened beverages include all types of beverages containing free sugars and these include carbonated or non-carbonated soft drinks, fruit/vegetable juices and drinks, liquid and powder concentrates, flavoured water, energy and sports drinks, ready-to-drink tea, ready-to-drink coffee, and flavoured milk drinks.[20]<br><br>[†]Basis for indicator proposed by the GAMA AG

DRAFT

A2.03 Proportion of female adolescents (10-19 years) who were aware of menstruation before menarche, by age group (10-14, 15-19 years)

Definition	Proportion of female adolescents (10-19 years) who were aware of menstruation* before menarche, by age group (10-14, 15-19 years).
Numerator	Number of postmenarchal female adolescent respondents (10-19 years) who reported being aware of menstruation* before menarche.
Denominator	Total number of postmenarchal female adolescent respondents (10-19 years).
Preferred data source	Population-based survey
Alternative data source(s)	Not applicable
Proposed age range	10-19 years
Disaggregation	Age group (10-14, 15-19 years)
Indicator type	Outcome
Use status	Not currently in use
Initiatives using this indicator	None
Additional comments	This indicator is derived from an indicator proposed by the members of the Menstrual Hygiene and Health Management Meeting (March 2019; Geneva).[21]<br><br>*This is intended to assess the lowest level of knowledge regarding menstruation, i.e., that respondents knew what was happening to them when they first saw bleeding and/or were aware that this was something that would happen to them.

A2.04 Past 30 day prevalence of electronic cigarette use among adolescents (10–19 years), by age group (10-14, 15-19 years) and sex

Definition	Percentage of adolescents (10-19 years) who used electronic cigarettes on one or more days during the past 30 days, by age group (10-14, 15-19 years) and sex.
Numerator	Number of adolescent respondents (10-19 years) who reported using electronic cigarettes on one or more days during the past 30 days.
Denominator	Total population of adolescent respondents (10-19 years).
Preferred data source	Population-based survey
Alternative data source(s)	Not applicable
Proposed age range	10-19 years
Disaggregation	Age group (10-14, 15-19 years); sex
Indicator type	Outcome
Use status	In use with modifications
Initiatives using this indicator	Global Youth Tobacco Survey[†]
Additional comments	Current use of electronic cigarettes includes both daily or non-daily use. Current use of electronic cigarettes is assessed separately from cigarettes, other smoked tobacco products, and smokeless tobacco products and is not included in the current definition of current any tobacco use (See 2.05 for current tobacco use).[14]<br><br>[†]Basis for indicator proposed by the GAMA AG

DRAFT

3. Policies, programmes, and laws

Section summary

Indicators included within section (indicator details presented below summary):

- 3.01 Existence of an operational adolescent (10-19 years) health programme with coverage at the national level
- 3.02 Existence of national standards for delivery of health services to adolescents (10-19 years)
- A3.01 Existence of national policy exempting adolescents (10-19 years) from user fees for specified health services in the public sector, by type of service
- A3.02 Absence of legal age limit for married and unmarried adolescents (10-19 years) to provide consent, without spousal/parental/legal guardian consent, for specified adolescent health services, by marital status and type of service

Relevant indicators included in other sections

- None

Measurement gaps

- Measures of policy implementation and impact
- Overall indicator assessing adolescent right to health/healthcare

DRAFT

3.01 Existence of an operational adolescent (10-19 years) health programme with coverage at the national level

Definition	The country has an adolescent (10-19 years) health programme (stand-alone or integrated) at the national level or in all subnational (first administrative level) jurisdictions with at least one designated full-time person* and a regular government budget allocation to support the programme.
Numerator	<u>National level:</u> The country reports the existence of a national adolescent health programme (stand-alone or integrated) with at least one designated full-time person* and a regular government budget allocation to support the program.<br><br><u>Subnational level (first administrative level):</u> Each subnational jurisdiction reports the existence of a subnational adolescent health programme (stand-alone or integrated) with at least one full-time person* and a regular government budget allocation to support the program.
Denominator	Not applicable.
Preferred data source	Policy survey
Alternative data source(s)	Not applicable
Proposed age range	10-19 years
Disaggregation	Not applicable
Indicator type	Input and process
Use status	In use with modifications
Initiatives using this indicator	Countdown to 2030†
Additional comments	Data for this indicator are collected through the SRMNCAH Policy Survey[22] using the questions below:<br>1. Is there a regular government budget allocation to support the national adolescent health programme? Yes/No/Unknown<br>2. Is there at least one designated full-time person for the national adolescent health programme? Yes/No/Unknown<br><br>*The requirement of a single full-time person may also be satisfied by multiple individuals sharing a post.<br><br>†Basis for indicator proposed by the GAMA AG

3.02 Existence of national standards for delivery of health services to adolescents (10-19 years)

Definition	The country has national standards for delivery of health services specifically for adolescents (10-19 years) that include a clearly defined, comprehensive package of health services, the implementation of which has been monitored.
Numerator	The country reports the existence of national standards for delivery of health services to adolescents that include a clearly defined, comprehensive package of health services, the implementation of which has been monitored.
Denominator	Not applicable.
Preferred data source	Policy survey
Alternative data source(s)	Not applicable
Proposed age range	10-19 years
Disaggregation	Not applicable
Indicator type	Input and process
Use status	In use
Initiatives using this indicator	Countdown to 2030[†]
Additional comments	Data for this indicator are collected through the SRMNCAH Policy Survey[22] using the questions below.<br>1. Does the country have national standards for delivery of health services to adolescents? Yes/No/Unknown<br>If yes,<br>2. Do these standards include a clearly defined comprehensive package of health services for adolescents (10-19)? Yes/No/Unknown<br>3. Are activities being carried out to monitor the implementation of these adolescent health standards for delivery? Yes/No/Unknown<br><br>A list of currently recommended adolescent services and interventions is included within the Global Accelerated Action for the Health of Adolescents (AA-HA!).[23]<br><br>[†]Basis for indicator proposed by the GAMA AG

A3.01 Existence of national policy exempting adolescents (10-19 years) from user fees for specified health services in the public sector, by type of service

Definition	The existence of a national policy exempting adolescents from user fees for specified health services in the public sector (i.e., outpatient care visits, inpatient care visits, HIV testing and counselling, contraceptives, mental health, rehabilitation for substance abuse, pharmaceutical products and/or other medical supplies if required for diagnosis and treatment, testing and treatment for sexually transmitted infections and vaccination for human papilloma virus [HPV]), by type of service.
Numerator	Yes = All adolescents are exempted from user fees for the specified service<br>Partial = Selected adolescent population subgroups are exempted from user fees for the specified service<br>No = Adolescents are not exempted from user fees for the specified service.
Denominator	Not applicable
Preferred data source	Policy survey
Alternative data source(s)	Not applicable
Proposed age range	10-19 years
Disaggregation	Type of service
Indicator type	Inputs and processes
Use status	In use
Initiatives using this indicator	WHO SRMNCAH Policy Survey†
Additional comments	†Basis for indicator proposed by the GAMA AG

A3.02 Absence of legal age limit for married and unmarried adolescents (10-19 years) to provide consent, without spousal/parental/legal guardian consent, for specified adolescent health services, by marital status and type of service

Definition	The absence of a legal age limit to allow married and unmarried adolescents (10-19 years) to provide consent, without parental/legal guardian consent, for specified adolescent health services (i.e., contraceptive services except sterilization, emergency contraception, HIV testing and counselling services, HIV care and treatment, harm reduction interventions for injecting drug users and mental health services), by marital status and type of service.
Numerator	The country reports no legal age limit for providing consent to the specified service without spousal (married adolescents) or parental/legal guardian (unmarried adolescents) consent.
Denominator	Not applicable
Preferred data source	Policy survey
Alternative data source(s)	Not applicable
Proposed age range	10-19 years
Disaggregation	Marital status, type of service
Indicator type	Inputs and processes
Use status	In use
Initiatives using this indicator	WHO SRMNCAH Policy Survey†
Additional comments	†Basis for indicator proposed by the GAMA AG

DRAFT

4. Systems performance and interventions

Section summary

Indicators included within section (indicator details presented below summary):

- 4.01 Proportion of adolescents (10-19 years) who made a visit to a health facility to receive a health service during the past 12 months, by age group (10-14, 15-19 years) and sex
- 4.02 Proportion of 15-year-old adolescents covered by human papilloma virus (HPV) vaccine (last dose in schedule), by sex
- 4.03 Existence of age- and sex-disaggregated health data for adolescents (10-19 years) in the national health information system
- A4.01 Proportion of schools that offer comprehensive school health services
- A4.02 Proportion of schools that offer life skills-based HIV and sexuality education during the previous academic year

Relevant indicators included in other sections:

- 3.02 Existence of national standards for delivery of health services to adolescents (10-19 years)

Measurement gaps:

- Existence of routinely administered adolescent-specialized survey
- Indicator of implementation for standalone and/or integrated services
- Integration of adolescent-friendly services within primary health care system
- Measures of health service quality
- Prevention activities
- Proportion of health facilities providing adolescent-friendly services

DRAFT

4.01 Proportion of adolescents (10-19 years) who made a visit to a health facility to receive a health service during the past 12 months, by age group (10-14, 15-19 years) and sex

Definition	Proportion of adolescents (10-19 years) who visited a health facility* to receive a health service during the past 12 months, by age group (10-14, 15-19 years) and sex.
Numerator	Number of adolescent respondents (10-19 years) who reported visiting to a health facility* to receive a health service during the past 12 months.
Denominator	Total number of adolescent respondents (10-19 years).
Preferred data source	HMIS
Alternative data source(s)	Population-based survey
Proposed age range	10-19 years
Disaggregation	Age group (10-14, 15-19 years); sex
Indicator type	Outcome
Use status	Not currently in use
Initiatives using this indicator	Global Reference List of Health Indicators for Adolescents[†]
Additional comments	*A visit to a health facility includes a visit to a hospital, health centre, clinic, doctor's office, but not a visit to a pharmacy, traditional healer, or herbalist. The health facility may be in any health sector (e.g., public, private, other). Where relevant, additional disaggregation by health sector may be considered.<br><br>A list of currently recommended adolescent services and interventions is included within the AA-HA!.[23]<br><br>[†]Basis for indicator proposed by the GAMA AG

DRAFT

4.02 Proportion of 15-year-old adolescents covered by human papilloma virus (HPV) vaccine (last dose in schedule), by sex

Definition	Proportion of 15-year-old adolescents who received the recommended doses of HPV vaccine, by sex.
Numerator	Number of 15-year-old adolescents who received the recommended doses of HPV vaccination at any age.
Denominator	Total number of 15-year-old adolescents.
Preferred data source	HMIS
Alternative data source(s)	Population-based survey
Proposed age range	<16 years
Disaggregation	Sex
Indicator type	Outcome
Use status	In use
Initiatives using this indicator	Global Strategy for WCAH; SDGs (3.b.1)[†]
Additional comments	[†]Basis for indicator proposed by the GAMA AG

DRAFT

4.03 Existence of age- and sex-disaggregated health data for adolescents (10-19 years) in the national health information system

Definition	National health information system* includes data on adolescent (10-19 years) health indicators, broken down by age group (10-14, 15-19 years) and sex.
Numerator	For calculation, see "Additional comments" below.
Denominator	Not applicable.
Preferred data source	HMIS
Alternative data source(s)	Not applicable
Proposed age range	10-19 years
Disaggregation	Not applicable
Indicator type	Inputs and processes
Use status	In use
Initiatives using this indicator	EMRO core indicators for adolescent health†
Additional comments	*A health information system is defined as "the interrelated component parts for acquiring and analysing data and providing information (management information; health statistics; health literature) for the management of a health programme or system and for monitoring health activities."[24]<br><br>Metadata for the EMRO core indicators for adolescent health[25] provide the additional computational details:<br><br>Calculated as an index.<br>Score 0 = No adolescent health age- and sex-disaggregated data in the health information system<br>Score 1 = Data included in the health information system but not in full (either one of the criteria is not met)<br>Score 2 = Data included in the health information system in full (both criteria are present)<br><br>Criteria<br>1. Age groups included: 10–14 and 15–19 years<br>2. Sex-disaggregated<br><br>†Basis for indicator proposed by the GAMA AG

A4.01 Proportion of schools that offer comprehensive school health services

Definition	Proportion of schools that report offering comprehensive school health services, defined as school health services addressing at least four health areas relevant to their student population, including: positive health and development; unintentional injury; violence; sexual and reproductive health including HIV; communicable disease; noncommunicable disease, sensory functions, physical disability, oral health, nutrition, and physical activity; and mental health, substance use and self-harm.
Numerator	Number of schools that report offering school health services that address at least four of the following health areas: positive health and development; unintentional injury; violence; sexual and reproductive health including HIV; communicable disease; noncommunicable disease, sensory functions, physical disability, oral health, nutrition, and physical activity; and mental health, substance use and self-harm.
Denominator	Total number of responding schools.
Preferred data source	Policy survey
Alternative data source(s)	Not applicable
Proposed age range	Not applicable
Disaggregation	None
Indicator type	Output
Use status	Not currently in use
Initiatives using this indicator	None
Additional comments	The operational definition of comprehensive school health services provided above is based on a definition provided in the recently published WHO Guideline on School Health Services.[26]<br><br>†Basis for indicator proposed by the GAMA AG

A4.02 Proportion of schools that offer life skills-based HIV and sexuality education during the previous academic year

Definition	Proportion of schools that report offering life skills-based HIV and sexuality education (i.e., education on life skills, sexual and reproductive health, sexuality, and HIV transmission and prevention) during the previous academic year within the formal curriculum.
Numerator	*School Policy Survey:* Number of responding schools that provided life skills-based HIV and sexuality education during the previous academic year according to a combination of all essential topics and at least six desirable topics in the questionnaire, as part of the formal curriculum.<br><br>*Annual School Census:* Number of responding schools that report teaching all three of the following within the formal curriculum: generic life skills, sexual and reproductive health/sexuality education, and HIV transmission and prevention.
Denominator	Total number of responding schools.
Preferred data source	Policy survey
Alternative data source(s)	Annual school census
Proposed age range	Not applicable
Disaggregation	None
Indicator type	Output
Use status	In use
Initiatives using this indicator	SDGs (thematic indicator 4.7.2)[†]; UNECE Monitoring Framework (ICPD)
Additional comments	Additional details of the proposed assessment methods are available in the SDG indicator metadata.[27]<br><br>[†]Basis for indicator proposed by the GAMA AG

5. Subjective well-being

Section summary

Indicators included within section (indicator details presented below summary):

- 5.01 Proportion of adolescents (10-19 years) with someone to talk to when they have a worry or problem, by age group (10-14, 15-19 years) and sex
- A5.01 Proportion of adolescents (10-19 years) with a positive connection with their parent or guardian, by age group (10-14, 15-19 years) and sex

Relevant indicators included in other sections:

- None

Measurement gaps:

- Measures of positive well-being
- Positive youth development
- Resilience, protective factors, supportive assets
- Sleep

DRAFT

5.01 Proportion of adolescents (10-19 years) with someone to talk to when they have a worry or problem, by age group (10-14, 15-19 years) and sex

Definition	Proportion of adolescents (10-19 years) who were able to talk to someone*, either most or all of the time, when they have a problem or worry having to do with difficult feelings and experiences, by age group (10-14, 15-19 years) and sex.
Numerator	Number of adolescent respondents (10-19 years) who reported being able to talk to someone*, either most or all of the time, when they have a problem or worry having to do with difficult feelings and experiences.
Denominator	Total number of adolescent respondents (10-19 years).
Preferred data source	Population-based survey
Alternative data source(s)	Not applicable
Proposed age range	10-19 years
Disaggregation	Age group (10-14, 15-19 years); sex
Indicator type	Outcome
Use status	In use
Initiatives using this indicator	MMAP[†]
Additional comments	*Talking to someone is defined as having any sort of meeting or contact with anyone about these sorts of problems or worries.[28,29]<br><br>[†]Basis for indicator proposed by the GAMA AG

DRAFT

A5.01 Proportion of adolescents (10-19 years) with a positive connection with their parent or guardian, by age group (10-14, 15-19 years) and sex

Definition	Proportion of adolescents (10-19 years) with parents/guardians who understood their problems or worries most or all of the time during the past 30 days, by age group (10-14, 15-19 years) and sex.
Numerator	Number of adolescent respondents (10-19 years) who reported that their parents or guardians understood their problems or worries most or all of the time during the past 30 days.
Denominator	Total number of adolescent respondents (10-19 years).
Preferred data source	Population-based survey
Alternative data source(s)	Not applicable
Proposed age range	10-19 years
Disaggregation	Age group (10-14, 15-19 years); sex
Indicator type	Impact
Use status	In use
Initiatives using this indicator	MMAP†
Additional comments	†Basis for indicator proposed by the GAMA AG

DRAFT

6. Health outcomes and conditions

Section summary

Indicators included within section (indicator details presented below summary):

- 6.01 Adolescent (10-19 years) mortality rate, by age group (10-14, 15-19 years) and sex
- 6.02 Adolescent (10-19 years) mortality rate, by specified causes of death, age group (10-14, 15-19 years) and sex
- 6.03 Number of new cases of HIV infections among adolescents (10-19 years) per 1,000 uninfected adolescent population, by age group (10-14, 15-19 years) and sex
- 6.04 Number of new cases of sexually transmitted infections (STIs) among adolescents (10-19 years), by age group (10-14, 15-19 years) and sex
- 6.05 Proportion of adolescents (10-19 years) who report a suicide attempt during the past 12 months, by age group (10-14, 15-19 years) and sex
- 6.06 Proportion of adolescents (10-19 years) who report symptoms of depression and/or anxiety, by age group (10-14, 15-19 years) and sex
- 6.07 Number of new cases of specified types of injuries among adolescents (10–19 years) per 100,000 population, type of injury, age group (10-14, 15-19 years) and sex
- 6.08 Proportion of adolescents (10-19 years) who experienced physical violence during the past 12 months, by perpetrator (parents/caregivers, teachers, other adults, intimate partners, peers), age group (10-14, 15-19 years) and sex
- 6.09 Proportion of adolescents (10-19 years) who experienced contact sexual violence during the past 12 months, by perpetrator (parents/caregivers, teachers, other adults, intimate partners, peers), age group (10-14, 15-19 years) and sex
- 6.10 Adolescent (10-19 years) birth rate, by age group (10-14, 15-19 years)
- 6.11 Prevalence of anaemia among adolescents (10-19 years), by age group (10-14, 15-19 years) and sex
- A6.01 Proportion of adolescents (10-19 years) perpetrating physical violence during the past 12 months, by age group (10-14, 15-19 years) and sex
- A6.02 Proportion of young women and men (18-29 years) who experienced sexual violence by age 18, by perpetrator (parents/caregivers, teachers, intimate partners, peers), age at victimization (<10, 10-14, 15-18 years) and sex
- A6.03 Proportion of adolescents (10-19 years) who report suicidal thoughts during the past 2 weeks, by age group (10-14, 15-19 years) and sex
- A6.04 Proportion of adolescents (10-19 years) with symptoms of anxiety or depression who report contact with a health professional or counsellor for their mental health symptoms, by age group (10-14, 15-19 years) and sex

DRAFT

- A6.05 Proportion of female adolescents (10-19 years) who have undergone female genital mutilation/cutting, by age group (10-14, 15-19 years)

Relevant indicators included in other sections:

- None

Measurement gaps:

- Adolescent pregnancy
- Disability
- Overall measure of mental health
- Self-rated health
- Stress, post-traumatic stress

DRAFT

6.01 Adolescent (10-19 years) mortality rate, by age group (10-14, 15-19 years) and sex

Definition	Number of deaths among adolescents (10-19 years) per 100,000 adolescent population during a year, by age group (10-14, 15-19 years) and sex.
Numerator	Number of deaths among adolescents (10-19 years) x 100,000 during a given year.
Denominator	Total adolescent (10-19 years) population during the same year.
Preferred data source	CRVS
Alternative data source(s)	Population-based survey
Proposed age range	10-19 years
Disaggregation	Age group (10-14, 15-19 years); sex
Indicator type	Impact
Use status	In use
Initiatives using this indicator	Adolescent Country Tracker; Countdown to 2030; Global Reference List of 100 Core Health Indicators; Global Reference List of Health Indicators for Adolescents; Global Strategy for WCAH[†]; Youth Development Index
Additional comments	[†]Basis for indicator proposed by the GAMA AG

DRAFT

6.02 Adolescent (10-19 years) mortality rate, by specified causes of death, age group (10-14, 15-19 years) and sex

Definition	Number of deaths among adolescents (10-19 years) per 100,000 adolescent population during a year, by specified causes, including priority causes of adolescent death globally (i.e., road traffic injury, interpersonal violence, drowning, self-harm, maternal conditions, diarrhoeal diseases, and HIV/AIDS) and other causes determined by the national context, by age group (10-14, 15-19 years) and sex.
Numerator	Number of deaths among adolescents (10-19 years) due to specified causes x 100,000 during a given year.
Denominator	Total adolescent (10-19 years) population during the same year.
Preferred data source	CRVS
Alternative data source(s)	Population-based surveys; National surveillance system; HMIS
Proposed age range	10-19 years
Disaggregation	Cause (leading global causes, such as injuries, homicide, suicide, maternal conditions, and HIV, as well as other relevant causes determined by national context); age group (10-14, 15-19 years); sex
Indicator type	Impact
Use status	In use with modifications
Initiatives using this indicator	Countdown to 2030[†]; Global Strategy for WCAH
Additional comments	[†]Basis for indicator proposed by the GAMA AG

DRAFT

6.03 Number of new cases of HIV infections among adolescents (10-19 years) per 1,000 uninfected adolescent population, by age group (10-14, 15-19 years) and sex

Definition	Number of new cases of HIV infections among adolescents (10-19 years) per 1,000 uninfected adolescent population (defined as the number of new HIV infections per 1,000 person-years among the uninfected population), by age group (10-14, 15-19 years) and sex.
Numerator	See "Additional comments" below.
Denominator	See "Additional comments" below.
Preferred data source	HMIS
Alternative data source(s)	Population-based survey; Other data source (see "Additional comments" below)
Proposed age range	10-19 years
Disaggregation	Age group (10-14, 15-19 years); sex
Indicator type	Impact
Use status	In use
Initiatives using this indicator	Global Reference List of 100 Core Health Indicators; Global Strategy for WCAH; SDGs (3.3.1)[†]; UNECE Monitoring Framework (ICPD); WHO GPW 13
Additional comments	The SDG indicator metadata[8] provide the additional computational details:<br><br>Computation method: Longitudinal data on individuals are the best source of data but are rarely available for large populations. Special diagnostic tests in surveys or from health facilities can be used to obtain data on HIV incidence. HIV incidence is thus modelled using the Spectrum software.<br><br>[†]Basis for indicator proposed by the GAMA AG

DRAFT

6.04 Number of new cases of sexually transmitted infections (STIs) among adolescents (10-19 years), by age group (10-14, 15-19 years) and sex

Definition	Number of new cases of STIs (syndromic or etiological reporting) other than HIV among adolescents (10-19 years) per 100,000 adolescent population during a year, by age group (10-14, 15-19 years) and sex.
Numerator	Number of new cases of STIs other than HIV among adolescents (10-19 years) during a given year x 100,000.
Denominator	Total population of adolescents (10-19 years) during the same year.
Preferred data source	HMIS
Alternative data source(s)	Not applicable
Proposed age range	10-19 years
Disaggregation	Age group (10-14, 15-19 years); sex
Indicator type	Impact
Use status	In use with modifications
Initiatives using this indicator	Global Reference List of 100 Core Health Indicators[†]
Additional comments	[†]Basis for indicator proposed by the GAMA AG

DRAFT

6.05 Proportion of adolescents (10-19 years) who report a suicide attempt during the past 12 months, by age group (10-14, 15-19 years) and sex

Definition	Proportion of adolescents (10-19 years) who reported a suicide attempt* during the past 12 months, by age group (10-14, 15-19 years) and sex.
Numerator	Number of adolescent respondents (10-19 years) who reported a suicide attempt* during the past 12 months.
Denominator	Total number of adolescent respondents (10-19 years).
Preferred data source	Population-based survey
Alternative data source(s)	Not applicable
Proposed age range	10-19 years
Disaggregation	Age group (10-14, 15-19 years); sex
Indicator type	Impact
Use status	In use
Initiatives using this indicator	EMRO core indicators for adolescent health; MMAP†
Additional comments	*A suicide attempt is defined as "An act with non-fatal outcome in which an individual deliberately initiates a non-habitual behaviour that, without intervention from others, will cause self-harm, or deliberately ingests a substance in excess of the prescribed dosage, and which is aimed at realizing changes that the person desires via the actual or expected physical consequences."[30]<br><br>†Basis for indicator proposed by the GAMA AG

6.06 Proportion of adolescents (10-19 years) who report symptoms of depression and/or anxiety, by age group (10-14, 15-19 years) and sex

Definition	Proportion of adolescents (10-19 years) with symptoms of depression and/or anxiety at a clinical threshold*, by age group (10-14, 15-19 years) and sex.
Numerator	Number of adolescent respondents (10-19 years) who reported symptoms of depression and/or anxiety at a clinical threshold*.
Denominator	Total number of adolescent respondents (10-19 years).
Preferred data source	Population-based survey
Alternative data source(s)	Not applicable
Proposed age range	10-19 years
Disaggregation	Age group (10-14, 15-19 years), sex
Indicator type	Impact
Use status	Not currently in use
Initiatives using this indicator	EMRO core indicators for adolescent health; Global Reference List of Health Indicators for Adolescents; Global Strategy for WCAH; MMAP[†]
Additional comments	*Indicators were developed to assess and monitor overall burden of a major depressive episode or anxiety disorder based on a level of symptoms consistent with clinical diagnosis according to the Diagnostic and Statistical Manual of Mental Disorders, 5th Edition (DSM-5) and International Classification of Diseases 11th Revision (ICD-11).[28,29]<br><br>[†]Basis for indicator proposed by the GAMA AG

DRAFT

6.07 Number of new cases of specified types of injuries among adolescents (10–19 years) per 100,000 population, type of injury, age group (10-14, 15-19 years) and sex

Definition	Number of new cases of specific types of injuries (i.e., road traffic injuries, fire-related burns, poisonings, falls, and drowning) among adolescents (10-19 years) per 100,000 adolescent population during a year, by age group (10-14, 15-19 years) and sex.
Numerator	Number of new cases of a specific type of injuries (i.e., road traffic injuries, fire-related burns, poisonings, falls, and drowning) among adolescents (10-19 years) during a given year x 100,000.
Denominator	Total adolescent population (10-19 years) during the same year.
Preferred data source	HMIS
Alternative data source(s)	Population-based survey; National surveillance system
Proposed age range	10-19 years
Disaggregation	Injury type (road traffic injuries, fire-related burns, poisoning, falls, and drowning); age group (10-14, 15-19 years); sex
Indicator type	Impact
Use status	In use
Initiatives using this indicator	EMRO core indicators for adolescent health[†]
Additional comments	The types of injuries listed above were selected for their global relevance. Reporting of additional injury types may be considered based on the national and regional context.<br><br>[†]Basis for indicator proposed by the GAMA AG

6.08 Proportion of adolescents (10-19 years) who experienced physical violence during the past 12 months, by perpetrator (parents/caregivers, teachers, other adults, intimate partners, peers), age group (10-14, 15-19 years) and sex

Definition	Proportion of adolescents (10-19 years) who experienced physical violence (excluding sexual violence) during the past 12 months, by perpetrator (parents/caregivers, teachers, other adults, intimate partners, peers), age group (10-14, 15-19 years) and sex.
Numerator	Number of adolescent respondents (10-19 years) who reported experiencing physical violence (excluding sexual violence) during the past 12 months.
Denominator	Total number of adolescent respondents (10-19 years).
Preferred data source	Population-based survey
Alternative data source(s)	Not applicable
Proposed age range	10-19 years
Disaggregation	Perpetrator (parents/caregivers, teachers, other adults, intimate partners, peers); age group (10-14, 15-19 years); sex
Indicator type	Impact
Use status	In use with modifications
Initiatives using this indicator	FRESH; INSPIRE[†]
Additional comments	[†]Basis for indicator proposed by the GAMA AG

DRAFT

6.09 Proportion of adolescents (10-19 years) who experienced contact sexual violence during the past 12 months, by perpetrator (parents/caregivers, teachers, other adults, intimate partners, peers), age group (10-14, 15-19 years) and sex

Definition	Proportion of adolescents (10-19 years) who experienced sexual violence involving physical contact (i.e., forced (completed) sex; pressured or coerced (completed) sex; attempted (but not completed) forced, coerced, or pressured sex; unwanted, non-consensual sexual touch) during the past 12 months, by perpetrator (parents/caregivers, teachers, other adults, intimate partners, peers), age group (10-14, 15-19 years) and sex.
Numerator	Number of adolescent respondents (10-19 years) who reported experiencing contact sexual violence during the past 12 months.
Denominator	Total number of adolescent respondents (10-19 years).
Preferred data source	Population-based survey
Alternative data source(s)	Not applicable
Proposed age range	10-19 years
Disaggregation	Perpetrator (parents/caregivers, teachers, other adults, intimate partners, peers); age group (10-14, 15-19 years); sex
Indicator type	Impact
Use status	In use with modifications
Initiatives using this indicator	INSPIRE†
Additional comments	Sexual violence may take many forms. The items included within this indicator represent the subset of forms of sexual violence involving physical contact. Examples of non-contact forms of sexual violence may include: Non-contact, in person acts such as verbal sexual harassment, forced to undress, exposure, etc.; online sexual abuse; and sexual exploitation.[16]<br><br>†Basis for indicator proposed by the GAMA AG

6.10 Adolescent (10-19 years) birth rate, by age group (10-14, 15-19 years)

Definition	Number of live births to female adolescents (10-19 years) per 1,000 female adolescents during a year, by age group (10-14, 15-19 years).
Numerator	Number of live births to female adolescents (10-19 years) during a given year.
Denominator	Total number of female adolescents (10-19 years) during the same year.
Preferred data source	CRVS
Alternative data source(s)	Population-based surveys; HMIS
Proposed age range	10-19 years
Disaggregation	Age group (10-14, 15-19 years)
Indicator type	Impact
Use status	In use with modifications
Initiatives using this indicator	Adolescent Country Tracker; Countdown to 2030; EMRO core indicators for adolescent health; FP2020; Global Reference List of Health Indicators for Adolescents[†]; Global Reference List of 100 Core Health Indicators; Global Strategy for WCAH; Lancet Commission on Adolescent Health and Wellbeing; SDGs (3.7.2); UNECE Monitoring Framework (ICPD); Youth Development Index
Additional comments	[†]Basis for indicator proposed by the GAMA AG

DRAFT

6.11 Prevalence of anaemia among adolescents (10-19 years), by age group (10-14, 15-19 years) and sex

Definition	Proportion of adolescents (10-19 years) who have a haemoglobin level below 11 g/dl for males and 12 g/dl for females, by age group (10-14, 15-19 years) and sex.
Numerator	Number of female adolescent respondents (10-19 years) who have a haemoglobin level less than 11 g/dl and male adolescents less than 12 g/dl at sea level.
Denominator	Total population of female and male adolescent respondents (10-19 years).
Preferred Data source	Population-based survey
Alternative data source(s)	National surveillance system
Proposed age range	10-19 years
Disaggregation	Age group (10-14, 15-19 years); sex
Indicator type	Outcome
Use status	In use
Initiatives using this indicator	Global Strategy for WCAH; EMRO core indicators for adolescent health[†]; Lancet Commission on Adolescent Health and Wellbeing
Additional comments	[†]Basis for indicator proposed by the GAMA AG

A6.01 Proportion of adolescents (10-19 years) perpetrating physical violence during the past 12 months, by age group (10-14, 15-19 years) and sex

Definition	Proportion of adolescents (10-19 years) who perpetrated physical violence (excluding sexual violence) during the past 12 months, by age group (10-14, 15-19 years) and sex.
Numerator	Number of adolescent respondents (10-19 years) who reported perpetrating physical violence (excluding sexual violence) during the past 12 months.
Denominator	Total number of adolescent respondents (10-19 years).
Preferred data source	Population-based survey
Alternative data source(s)	Not applicable
Proposed age range	10-19 years
Disaggregation	Age group (10-14, 15-19 years); sex
Indicator type	Impact
Use status	Not currently in use
Initiatives using this indicator	None
Additional comments	

DRAFT

A6.02 Proportion of young women and men (18-29 years) who experienced sexual violence by age 18, by perpetrator (parents/caregivers, teachers, intimate partners, peers), age at victimization (<10, 10-14, 15-18 years) and sex

Definition	Proportion of young women and men (18-29 years) who experienced sexual violence by age 18, by perpetrator (parents/caregivers, teachers, other adults, intimate partners, peers), age at victimization (<10, 10-14, 15-18 years) and sex.
Numerator	Number of female and male respondents (18-29 years) who reported experiencing any sexual violence by age 18.
Denominator	Total number female and male respondents (18-29 years).
Preferred data source	Population-based survey
Alternative data source(s)	Not applicable
Proposed age range	By age 18 years
Disaggregation	Perpetrator (parents/caregivers, teachers, intimate partners, peers); age group at victimization (<10, 10-14, 15-18 years); sex
Indicator type	Outcome
Use status	In use
Initiatives using this indicator	Global Reference List of 100 Core Health Indicators; Global Strategy for WCAH; INSPIRE; SDGs (16.2.3)[†]; UNECE Monitoring Framework (ICPD)
Additional comments	The SDG indicator metadata[8] provide the following additional detail:<br><br>Sexual violence comprises any sexual activities imposed by an adult on a child against which the child is entitled to protection by criminal law. This includes: (a) The inducement or coercion of a child to engage in any unlawful or psychologically harmful sexual activity; (b) The use of children in commercial sexual exploitation; (c) The use of children in audio or visual images of child sexual abuse; and (d) Child prostitution, sexual slavery, sexual exploitation in travel and tourism, trafficking for purposes of sexual exploitation (within and between countries), sale of children for sexual purposes and forced marriage. Sexual activities are also considered as abuse when committed against a child by another child if the offender is significantly older than the victim or uses power, threat or other means of pressure. Consensual sexual activities between children are not considered as sexual abuse if the children are older than the age limit defined by the State Party.

DRAFT

	[†]Basis for indicator proposed by the GAMA AG

DRAFT

A6.03 Proportion of adolescents (10-19 years) who report suicidal thoughts during the past 2 weeks, by age group (10-14, 15-19 years) and sex

Definition	Proportion of adolescents (10-19 years) who report suicidal thoughts during the past 2 weeks, by age group (10-14, 15-19 years) and sex.
Numerator	Number of adolescent respondents (10-19 years) who reported suicidal thoughts during the past two weeks.
Denominator	Total number of adolescent respondents (10-19 years).
Preferred data source	Population-based survey
Alternative data source(s)	Not applicable
Proposed age range	10-19 years
Disaggregation	Age group (10-14, 15-19 years); sex
Indicator type	Impact
Use status	In use with modifications
Initiatives using this indicator	EMRO core indicators for adolescent health; MMAP†
Additional comments	†Basis for indicator proposed by the GAMA AG

DRAFT

A6.04 Proportion of adolescents (10-19 years) with symptoms of anxiety or depression who report contact with a health professional or counsellor for their mental health symptoms, by age group (10-14, 15-19 years) and sex

Definition	Proportion of adolescents (10-19 years) with symptoms of anxiety or depression* who report contact with a health professional or counsellor for their mental health symptoms, by age group (10-14, 15-19 years) and sex.
Numerator	Number of adolescent respondents (10-19 years) who reported symptoms of anxiety and/or depression* and contact with a health professional or counsellor for mental health care.
Denominator	Number of adolescent respondents (10-19 years) who reported symptoms of anxiety and/or depression*.
Preferred data source	Population-based survey
Alternative data source(s)	Not applicable
Proposed age range	10-19 years
Disaggregation	Age group (10-14, 15-19 years); sex
Indicator type	Outcome
Use status	Not currently in use
Initiatives using this indicator	Global Reference List of 100 Core Health Indicators; MMAP[†]
Additional comments	*Indicators were developed to assess and monitor overall burden of a major depressive episode or anxiety disorder based on a level of symptoms consistent with clinical diagnosis according to the DSM-5 and ICD-11.[28,29]<br><br>[†]Basis for indicator proposed by the GAMA AG

A6.05 Proportion of female adolescents (10-19 years) who have undergone female genital mutilation/cutting, by age group (10-14, 15-19 years)

Definition	Proportion of female adolescents (10-19 years) who have undergone female genital mutilation/cutting, by age group (10-14, 15-19 years).
Numerator	Number of female adolescent respondents (10-19 years) who reported having undergone female genital mutilation/cutting.
Denominator	Total number of female adolescent respondents (10-19 years).
Preferred data source	Population-based survey
Alternative data source(s)	Not applicable
Proposed age range	10-19 years
Disaggregation	Age group (10-14, 15-19 years)
Indicator type	Outcome
Use status	In use with modifications
Initiatives using this indicator	Countdown to 2030; Global Reference List of 100 Core Health Indicators; SDGs (5.2.3)[†]
Additional comments	[†]Basis for indicator proposed by the GAMA AG

DRAFT

References

1. World Health Organization. The Global Action for Measurement of Adolescent health (GAMA). https://www.who.int/data/maternal-newborn-child-adolescent/gama (accessed 16 April 2021).

2. Guthold R, Moller AB, Azzopardi P, et al. The Global Action for Measurement of Adolescent health (GAMA) Initiative-Rethinking Adolescent Metrics. *Journal of Adolescent Health* 2019; **64**(6): 697-9.

3. Guthold R, Moller AB, Adebayo E, et al. Priority Areas for Adolescent Health Measurement. *Journal of Adolescent Health* 2021.

4. Newby H, Marsh AD, Moller A-B, et al. A Scoping Review of Adolescent Health Indicators. *Journal of Adolescent Health* 2021.

5. Lime Survey. Professional online surveys with LimeSurvey. 2020. https://www.limesurvey.org/ (accessed 19 June 2020).

6. Diaz T, Strong KL, Cao B, et al. A call for standardised age-disaggregated health data. *The Lancet Healthy Longevity* 2021; **2**(7): e436-e43.

7. United Nations, Department of Economic and Social Affairs, Population Division. World Population Prospects 2019: Methodology of the United Nations population estimates and projections: United Nations, 2019.

8. United Nations, Department of Economic and Social Affairs, Statistics Division. SDG Indicators - Metadata repository. https://unstats.un.org/sdgs/metadata/ (accessed 12 October 2021).

9. Food and Agriculture Organization of the United Nations. Food Insecurity Experience Scale. https://www.fao.org/in-action/voices-of-the-hungry/fies/en/ (accessed 12 October 2021).

10. Cafiero C, Viviani S, Nord M. Food security measurement in a global context: The food insecurity experience scale. *Measurement* 2018; **116**: 146-52.

11. United Nations Educational, Scientific, and Cultural Organization. International Standard Classification of Education (ISCED). http://uis.unesco.org/en/topic/international-standard-classification-education-isced (accessed 12 October 2021).

12. Babor TF, Higgins-Biddle JC. Brief Intervention for Hazardous and Harmful Drinking: A Manual for Use in Primary Care, 2001.

13. World Health Organization. Lexicon of alcohol and drug terms. Geneva: World Health Organization; 1994.

14. World Health Organization. WHO global report on trends in prevalence of tobacco use 2000-2025. 3rd ed ed. Geneva: World Health Organization; 2019.

15. World Health Organization. WHO guidelines on physical activity and sedentary behaviour. Geneva: World Health Organization; 2020.

16. United Nations Children's Fund. INSPIRE Indicator Guidance and Results Framework - Ending Violence Against Children: How to define and measure change. New York, USA: UNICEF, 2018.

DRAFT

17. World Health Organization. Definition of skilled health personnel providing care during childbirth: the 2018 joint statement by WHO, UNFPA, UNICEF, ICM, ICN, FIGO and IPA. CC BY-NC-SA 3.0 IGO. Geneva: World Health Organization, 2018.
18. Croft TN, Marshall AMJ, Allen CK, et al. Current Use of Contraceptive Methods. Guide to DHS Statistics. Rockville, Maryland, USA: ICF; 2020.
19. World Health Organization, Johns Hopkins Bloomberg School of Public Health, Center for Communication Programs. Family planning: a global handbook for providers: evidence-based guidance developed through worldwide collaboration. 3rd ed ed. Geneva: World Health Organization; 2018.
20. World Health Organization. Tackling NCDs: 'best buys' and other recommended interventions for the prevention and control of noncommunicable diseases. CC BY-NC-SA 3.0 IGO. Geneva: World Health Organization, 2017.
21. Sommer M, Zulaika, G., Schmitt, M., Gruer, C. (Eds.). Monitoring Menstrual Health and Hygiene: Measuring Progress for Girls on Menstruation; Meeting Report. New York & Geneva: Columbia University and WSSCC, 2019.
22. World Health Organization. Sexual, reproductive, maternal, newborn, child and adolescent health: policy survey, 2018-2019: summary report. Geneva: World Health Organization; 2020.
23. World Health Organization. Global Accelerated Action for the Health of Adolescents (AA-HA!): guidance to support country implementation. Geneva: World Health Organization, 2017.
24. World Health Organization. Health information systems development and strengthening : guidance on needs assessment for national health information systems development. Geneva: World Health Organization; 2000.
25. World Health Organization, Regional Office for the Eastern Mediterranean,. Core indicators for adolescent health: a regional guide. Cairo, Egypt: World Health Organization. Regional Office for the Eastern Mediterranean, 2014.
26. World Health Organization, United Nations Educational, Scientific, and Cultural Organization. WHO guideline on school health services. Geneva: World Health Organization; 2021.
27. Cornu C, Liu Y. TCG4: Development of SDG thematic indicator 4.7.2: Section of Health and Education, Division for Inclusion, Peace and Sustainable Development. Education Sector. UNESCO, 2018.
28. Carvajal-Velez L, Harris Requejo J, Ahs JW, et al. Increasing Data and Understanding of Adolescent Mental Health Worldwide: UNICEF's Measurement of Mental Health Among Adolescents at the Population Level Initiative. *Journal of Adolescent Health* 2021.
29. UNICEF. Measurement of Mental Health Among Adolescents at the Population Level (MMAP). 2020. https://data.unicef.org/topic/child-health/mental-health/mmap/ (accessed 2 July 2020).
30. Platt S, Bille-Brahe U, Kerkhof A, et al. Parasuicide in Europe: the WHO/EURO multicentre study on parasuicide. I. Introduction and preliminary analysis for 1989. *Acta Psychiatr Scand* 1992; **85**(2): 97-104.

31. United Nations. General Assembly. A/RES/71/313. Global indicator framework for the Sustainable Development Goals and targets of the 2030 Agenda for Sustainable Development. New York, USA: United Nations, 2017.
32. Azzopardi PS, Hearps SJC, Francis KL, et al. Progress in adolescent health and wellbeing: tracking 12 headline indicators for 195 countries and territories, 1990–2016. *The Lancet* 2019; **393**(10176): 1101-18.
33. Every Woman Every Child. Indicator and Monitoring Framework for the Global Strategy for Women's, Children's and Adolescents' Health 2016-2030. New York, 2016.
34. Countdown to 2030: tracking progress towards universal coverage for reproductive, maternal, newborn, and child health. *Lancet* 2018; **391**(10129): 1538-48.
35. Family Planning 2020. FP 2020. 2018. https://www.familyplanning2020.org/ (accessed 4 December 2019).
36. UNICEF. Adolescent Country Tracker. 2018. https://data.unicef.org/resources/adolescent-country-tracker/ (accessed 4 December 2018).
37. World Health Organization. Global Reference List of 100 Core Health Indicators (plus health-related SDGs). Geneva, Switzerland, 2018.
38. World Health Organization. Global Reference List of Health Indicators for Adolescents (aged 10-19 years). Geneva, Switzerland, 2015.
39. The Commonwealth. The Commonwealth Youth Development Index. 2016. https://thecommonwealth.org/youthdevelopmentindex (accessed 7 April 2020).
40. UNESCO. Monitoring and Evaluation Guidane for School Health Programs. Paris, France: UNESCO, 2014.
41. UNESCO. Measuring the education sector response to HIV and AIDS. Guidelines for the construction and use of core indicators. Paris, France: UNESCO, 2013.
42. World Health Organization. WHO 13th General Programme of Work (GPW 13) Impact Framework: Targets and indicators. 2018. https://www.who.int/about/what-we-do/GPW13_WIF_Targets_and_Indicators_English.pdf (accessed 7 April 2020).

Annexes

Annex 1: Additional details on methods

List of 16 global and regional initiatives including adolescent health indicators

1. Global indicator framework for the Sustainable Development Goals and targets of the 2030 Agenda for Sustainable Development[31]
2. The Lancet Commission on Adolescent health and wellbeing[32]
3. Indicator and Monitoring Framework for the Global Strategy for Women's, Children's, and Adolescents' Health (2016-2030)[33]
4. Countdown to 2030[34]
5. Family Planning 2020[35]
6. Adolescent Country Tracker[36]
7. Global Reference List of 100 Core Health Indicators[37]
8. Global Reference List of Health Indicators for Adolescents (aged 10-19 years)[38]
9. Core Indicators for Adolescent Health: A Regional Guide (Eastern Mediterranean Regional Office)[25]
10. Commonwealth Youth Development Index[39]
11. INSPIRE Indicator Guidance and Results Framework[16]
12. Monitoring and Evaluation Guidance for School Health Programmes[40]
13. Measuring the Education Sector Response to HIV and AIDS: guidelines for the construction and use of core indicators[41]
14. UNECE Monitoring Framework for the ICPD Programme of Action beyond 2014
15. WHO's 13th General Programme of Work Impact Framework[42]
16. Measurement of Mental Health Among Adolescents at the Population Level[29]

Indicator scoring criteria

- Relevance: The indicator measures a specific construct in a priority area of interest and there is a clear, demonstrated relationship between the indicator and adolescent health.
- Feasibility: Data for the indicator can be obtained with reasonable and affordable effort.
- Validity: The indicator provides a robust assessment of the construct of interest, is sensitive to change in that construct, and has been field tested. The method of assessment produces consistent results that are comparable across time periods and settings.
- Usefulness: The indicator captures information that is easily understood and timely. The information is easy to communicate to stakeholders and facilitates investment and action in adolescent health strategies, priorities, or programming.

DRAFT

Annex 2: Adolescent health measurement domains, core measurement areas, and contents area

ADOLESCENT HEALTH MEASUREMENT DOMAIN	ADOLESCENT HEALTH CORE MEASUREMENT AREA	CONTENT AREA/ WORKING GROUP
Social, cultural, economic, educational, environmental determinants of health	Education level and schooling status	3. Determinants of health
	Income level and poverty	
	Population	5. Demographics, mortality, morbidity, disability
	Gender	6. Sexual and reproductive health
Health behaviours and risks	Weight status	4. Health behaviours and risks
	Alcohol use	
	Substance use, other than alcohol and tobacco	
	Tobacco use	
	Dietary behaviour	
	Physical activity	
	Sexual health	6. Sexual and reproductive health
	Reproductive health	
	Contraception	
	Bullying	7. Violence and injury
Policies, programmes, and laws	Adolescent health policies and plans	1. Policies, programmes, laws
	Adolescent health protective laws	
Systems performance and interventions	Health service availability and access	2. Systems performance and interventions
	Health service quality	
	Immunization	
	System for monitoring and surveillance of adolescent health	
Health outcomes and conditions	All-cause mortality	5. Demographics, mortality, morbidity, disability
	Cause-specific mortality	
	Disability	
	HIV/AIDS	6. Sexual and reproductive health
	STIs other than HIV/AIDS	
	Adolescent fertility	
	Road injury	7. Violence and injury
	Interpersonal violence	
	Sexual violence	
	Gender-based violence	
	Self-harm	8. Mental health
	Anxiety disorders	
	Depressive disorders	

NOTE: A ninth content area, "Wellbeing," was subsequently added based on recommendations made during the 4th GAMA Meeting (June 2020, virtual meeting).

DRAFT

Annex 3: Classification of adolescent health measurement areas

Members of the GAMA AG and others identified 99 adolescent health measurement areas, based on four inputs [(1) young people's perspectives; (2) priorities in countries; (3) adolescent disease burden; and (4) measurement areas included in the existing initiatives]. From this, the 16 members of the GAMA AG selected 33 core, 19 expanded, and 6 context-specific measurement areas. The table below presents the 99 measurement areas according to their classification.

Domain	Priority measurement areas			Not selected as priority
	Core	Expanded	Context-specific	
Social, cultural, economic, educational, environmental determinants of health	• Education level and schooling status<br>• Gender<br>• Income level and poverty<br>• Population	• Being part of a vulnerable group<br>• Environment and pollution<br>• Ethnicity<br>• Social support	• Child marriage<br>• Child labour<br>• Social and cultural norms	• Disaster risk reduction<br>• Employment status<br>• Water, sanitation, and hygiene
Health behaviours and risks	• Alcohol use<br>• Bullying<br>• Contraception<br>• Dietary behaviour<br>• Physical activity<br>• Reproductive health<br>• Sexual health<br>• Substance use, other than alcohol and tobacco<br>• Tobacco use<br>• Weight status	• Sedentary behaviour<br>• Sleep<br>• Social media and internet		• Gaming<br>• High fasting plasma glucose<br>• High LDL cholesterol<br>• High systolic blood pressure<br>• Menstruation
Policies, programmes, and laws	• Adolescent health policies and plans<br>• Adolescent health protective laws	• Adolescent health programmes<br>• Adolescents' participation in programming and planning		
Systems performance and interventions	• Health service availability and access<br>• Health service quality<br>• Immunization	• Health education<br>• Health service utilization and barriers<br>• School health	• Social protection	• Community health<br>• Financial protection and health expenditure<br>• Health check-ups

	• System for monitoring and surveillance of adolescent health			• Training and education in adolescent health for professionals
Subjective well-being		• Autonomy<br>• Social connectedness		• Affect, feeling, and emotion<br>• Life satisfaction<br>• Meaning and achievement<br>• Spirituality
Health outcomes and conditions	• All-cause mortality<br>• Adolescent fertility<br>• Anxiety disorders<br>• Cause-specific mortality<br>• Depressive disorders<br>• Disability<br>• Gender-based violence<br>• HIV/AIDS<br>• Interpersonal violence<br>• Road injury<br>• Self-harm<br>• Sexual violence<br>• STIs other than HIV/AIDS	• Asthma<br>• Collective violence and legal intervention<br>• Diabetes<br>• Drowning<br>• Maternal conditions	• Iron-deficiency<br>• Vitamin A deficiency	• Allergies<br>• Autism and Asperger syndrome<br>• Back or neck pain<br>• Brain and nervous system cancers<br>• Cardiovascular diseases<br>• Childhood behavioural disorders<br>• Cirrhosis of the liver<br>• Congenital anomalies<br>• Diarrhoeal diseases<br>• Ear diseases and disorders<br>• Eye diseases and disorders<br>• Female genital mutilation and cutting<br>• Leukaemia<br>• Lower respiratory infections<br>• Malaria<br>• Meningitis<br>• Migraine<br>• Multi-morbidity<br>• Oral conditions<br>• Perinatal conditions<br>• Sickle cell disorders and trait<br>• Skin diseases<br>• Stress and pressure<br>• Tuberculosis<br>• Worms

DRAFT
